# Sulfatase-cleavable linkers for antibody-drug conjugates†

**DOI:** 10.1039/c9sc06410a

**Published:** 2020-01-27

**Authors:** Jonathan D. Bargh, Stephen J. Walsh, Albert Isidro-Llobet, Soleilmane Omarjee, Jason S. Carroll, David R. Spring

**Affiliations:** Department of Chemistry, University of Cambridge Lensfield Road Cambridge CB2 1EW UK spring@ch.cam.ac.uk; Cancer Research UK Cambridge Institute, University of Cambridge Robinson Way Cambridge CB2 0RE UK; GSK Gunnels Wood Road Stevenage SG1 2NY UK

## Abstract

Antibody-drug conjugates (ADCs) are a class of targeted drug delivery agents combining the cell-selectivity of monoclonal antibodies (mAbs) and the cytotoxicity of small molecules. These two components are joined by a covalent linker, whose nature is critical to the efficacy and safety of the ADC. Enzyme-cleavable dipeptidic linkers have emerged as a particularly effective ADC linker type due to their ability to selectively release the payload in the lysosomes of target cells. However, these linkers have a number of drawbacks, including instability in rodent plasma and their inherently high hydrophobicity. Here we show that arylsulfate-containing ADC linkers are cleaved by lysosomal sulfatase enzymes to tracelessly release their payload, while circumventing the instability problems associated with dipeptide-linkers. When incorporated with trastuzumab and the highly potent monomethyl auristatin E (MMAE) payload, the arylsulfate-containing **ADC 2** and **ADC 3** were more cytotoxic than the non-cleavable **ADC 4** against HER2-positive cells, while maintaining selectivity over HER2-negative cells. We propose that the stability, solubility and synthetic tractability of our arylsulfate linkers make them an attractive new motif for cleavable ADC linkers, with clear benefits over the widely used dipeptidic linkers.

## Abstract 2

Arylsulfate-containing linkers are cleaved by lysosomal sulfatases to release payloads from ADCs at targeted cancer cells.

## Graphical abstract

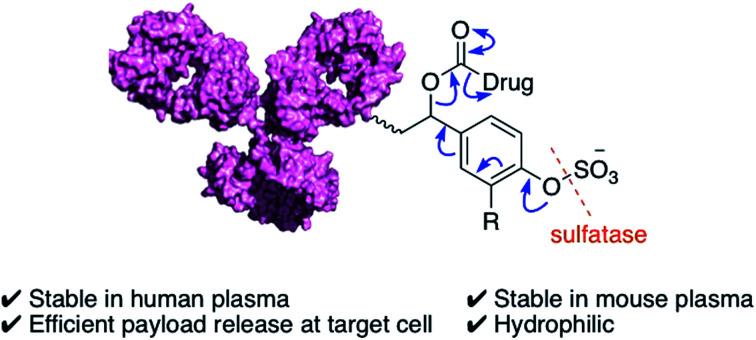

## Introduction

Antibody-drug conjugates (ADCs) are now established as an important class of therapeutics for the treatment of cancer. There are currently seven FDA-approved ADCs, with at least 60 more in clinical development.^1^ The success of ADCs arises from the combination of the exquisite cell-selectivity of monoclonal antibodies and the cytotoxicity of small molecule chemotherapies. However, for the mAb and drug to exert their maximum therapeutic potential, the covalent linker between the two groups must exhibit the following properties: (1) high plasma stability; the long circulatory lifetimes (*t*_1/2_ > 1 week) of ADCs places stringent stability requirements on the linker to avoid off-target payload release. (2) High aqueous solubility to aid bioconjugation of lipophilic payloads and avoid antibody aggregation. (3) Release of a potent cytotoxin from the antibody in the target cell.^2^

The majority of ADCs employ antibodies that target internalising antigens, overexpressed on the surface of certain cancer cells. Upon antibody-antigen binding at the target cell, the ADC is endocytosed and trafficked to the highly hydrolytic lysosomal compartments, where the antibody is broken down into its constituent amino acids.^3^ In the case of a non-cleavable linker, the active intracellular cytotoxin is released as the linker-payload with the terminal amino acid from the mAb still attached.^4^ Conversely, a cleavable linker is designed to release the unmodified drug at this point, or upon subsequent entry to the cytosol.

Cleavable linkers are generally preferred to non-cleavable linkers in ADC research for a number of reasons.^5^ First, traceless drug release allows the unmodified payload to perform its intracellular function without an unwanted linker appendage, thereby maximising cytotoxicity. Second, the unmodified payload can be uncharged, allowing it to diffuse into ‘bystander’ tumour cells that may not be expressing the target antigen. This ‘bystander effect’ provides an important mechanism for eradicating tumours with heterogeneous antigen expression.^6^

**Fig. 1:**
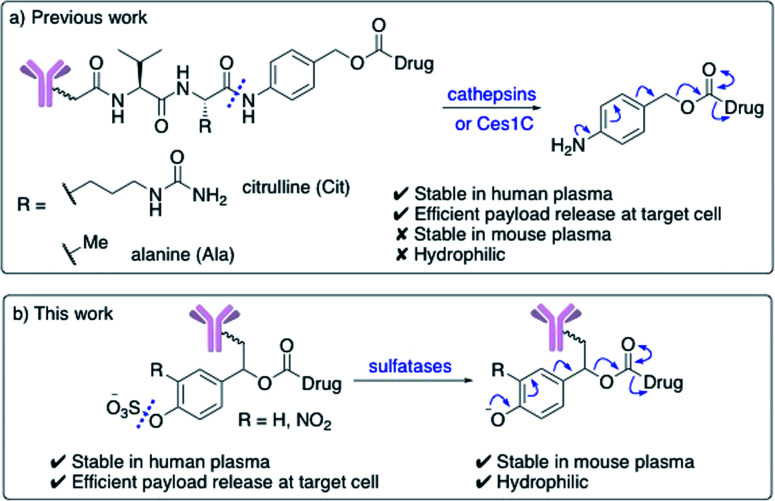
(a) Previously developed dipeptide linkers, cleaved by lysosomal cathepsins and mouse plasma enzyme Ces1C; (b) arylsulfate linkers for use in ADCs are cleaved by lysosomal sulfatases.

Enzyme-cleavable linkers are the most widely used group of ADC linkers. Other cleavable linkers, such as reducible disulfides or acid-sensitive motifs have been developed but their stability in human plasma is generally inferior.^7,8^ The benefits of enzyme-cleavable linkers include their ability to selectively induce drug release at target cells rather than in circulation. Thus far, the only clinically explored enzyme-cleavable linkers are peptides, sensitive to cleavage by lysosomal cathepsin proteases.^9–13^ Linkers containing Val–Cit or Val–Ala sequences are most widely employed, due to their high stability in human plasma and efficient drug release within the lysosomes of target cells (Fig. 1a). A self-immolative *para*-aminobenzoyl carbamate (PABC) spacer is also required, to ensure the cathepsin-mediated cleavage is unimpeded by the payload.

Unfortunately, dipeptidic linkers feature a number of major drawbacks. First, they are unstable in rodent blood, due to their susceptibility to the Ces1C hydrolase enzyme in rodent plasma.^7,14,15^ This hydrolysis causes premature payload release *in vivo* and limits the accuracy of preclinical efficacy and safety studies. Second, these linkers are hydrophobic, thereby hindering bioconjugation of lipophilic payloads and causing high levels of antibody aggregation upon conjugation.^16,17^ Despite these shortcomings, Val–Cit and Val–Ala dipeptides continue to be widely used in ADC research, routinely alongside monomethyl auristatin E (MMAE) or pyrrolobenzodiazepine- (PBD) dimer payloads respectively.

Other non-peptidic linkers have also been developed for ADCs. Linkers sensitive to the lysosomal β-glucuronidase and β-galactosidase enzymes have been described, which appear to address the problems associated with linker-payload hydrophobicity and rodent-plasma instability.^11,18^ However, their stereochemical complexity and possible overreliance on one specific lysosomal enzyme may explain their lack of development in clinically tested ADCs. Additionally, pyrophosphatase-cleavable motifs have also been described, for use with alcohol-linked glucocorticoid payloads.^19,20^

Novel linkers employing different modes of action are required to expand the ADC toolbox, given the therapeutic importance of the linker and the shortcomings of current cleavable linker technology.^5^ Herein, we describe the development of novel sulfatase-cleavable linkers for ADCs (Fig. 1b). Initial linker studies, facilitated by their simple synthesis, reveal the highly soluble arylsulfate linkers are stable in both human and mouse plasma. In addition, a series of arylsulfate-ADCs employing a trastuzumab antibody and MMAE as a payload were prepared and their cytotoxicity assessed against HER2-positive and HER2-negative cells.

## Results and discussion

### Design of linkers

Effective enzyme-cleavable ADC linkers must be highly stable in circulatory conditions but labile upon entry to the lysosomes of target cells, efficiently releasing their payloads. Sulfatases therefore offer an opportunity for selective payload release due to their high activity within the lysosomes and low activity in human and rodent plasma.^21^ A number of different sulfatases reside in the lysosome, catalysing the hydrolysis of alkylsulfate esters to alcohols.^22^ Although selective towards their natural substrates, they also each display arylsulfatase activity. Furthermore, sulfatases are overexpressed in a number of different cancer types, thereby offering the possibility of additional selectivity for arylsulfate-containing ADCs towards tumours.^23^ It was anticipated that the charged sulfates would be amenable to bioconjugation in aqueous media and their synthesis simplified by previously reported protecting group strategies.^24,25^ Accordingly, arylsulfate ADC linkers would potentially display significant advantages over dipeptide-based linkers.

**Fig. 2:**
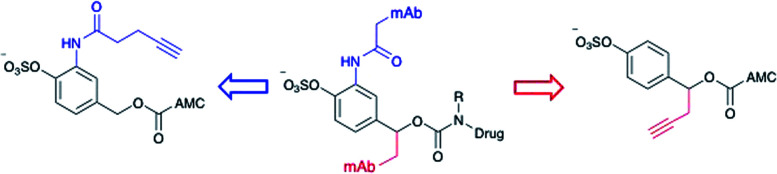
Design of linker-AMC model compounds. It was envisaged that the linker-payloads could be joined to the antibody through an *ortho*-amide (blue route) or a benzyl-alkyl (red route).

**Fig. 3:**
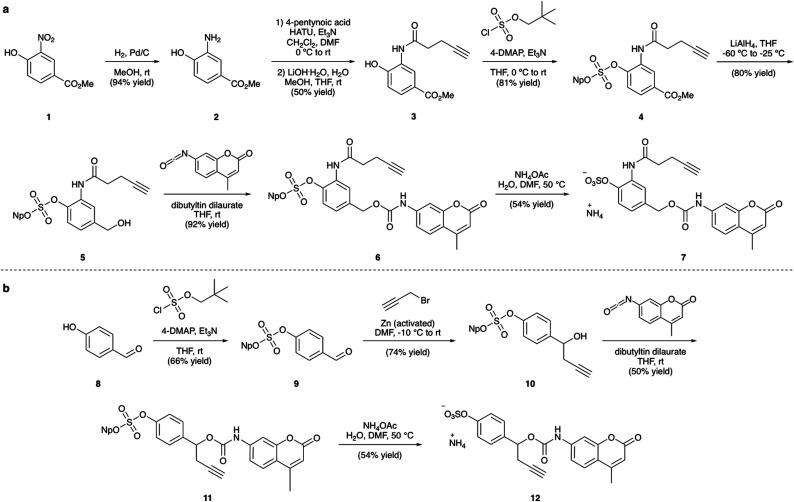
(a) Synthesis of linker-AMC **7** and (b) synthesis of linker-AMC **12**. Np = neopentyl.

Arylsulfate linker motifs were designed such that, upon hydrolysis, a 4-alkoxybenzyl carbamate would be revealed, primed for spontaneous 1,6-elimination of an amine-linked payload (Fig. 1b). To link to the antibody, we were first inspired by β-glucuronidase-cleavable linkers, which are linked to the mAb from an amide *ortho*- to the enzyme cleavable group (Fig. 2, blue route).^18^ It was also postulated that arylsulfates linked to the antibody by branching from the benzyl position would be less hindered at the cleavable sulfate, potentially improving sensitivity towards sulfatases (Fig. 2, red route). For our preliminary stability and release studies, we employed the fluorescent 7-amino-4-methyl coumarin (AMC) group as a model payload. AMC is widely used within enzymatic probes, as fluorescence is only observed upon release of the amino-coumarin from an amide or carbamate precursor.^26^ Therefore, through the use of fluorimetry, payload release at physiologically relevant concentrations could be measured in enzyme solutions and plasma. Model linkers **7** and **12** incorporate the two antibody-attachment designs, with AMC as their payload (Fig. 3).

### Synthesis of linker-coumarins

The synthesis of the arylsulfate linkers required a sulfate protecting group strategy to limit the number of steps requiring purification of charged molecules. The neopentyl moiety was chosen as an appropriate protecting group due to its stability, facile installation and functional group compatibility.^25^ Although harsh deprotection conditions are typically required, a procedure described by Simpson *et al.* employing aqueous NH_4_OAc appeared to offer a mild alternative.^27^ Synthesis of linker-AMC **7** was achieved in six steps from nitroarene **1** (Fig. 3a). First, **1** was hydrogenated to yield aniline **2**, which was then modified *via* HATU-mediated amide coupling to yield alkyne **3**. Next, installation of the neopentyl-protected sulfate was achieved by reaction with neopentyl sulfurochloridate. The resulting methyl ester **4** was then selectively reduced with LiAlH_4_ before reaction with AMC-isocyanate in the presence of a dibutyltin dilaurate catalyst afforded carbamate **6**. Gratifyingly, deprotection of the neopentyl sulfate with aqueous NH_4_OAc occurred smoothly to afford linker-AMC **7** in good overall yield. Linker-AMC **12** was synthesised *via* an analogous synthetic route (Fig. 3b). First, the neopentyl sulfate was introduced as before, with subsequent Barbier reaction with propargyl bromide and zinc powder affording alcohol **10**. Finally, reaction with AMC-isocyanate was followed by neopentyl deprotection to generate linker-AMC **12** with moderate to good yields throughout.

### Stability and release studies

**Fig. 4:**
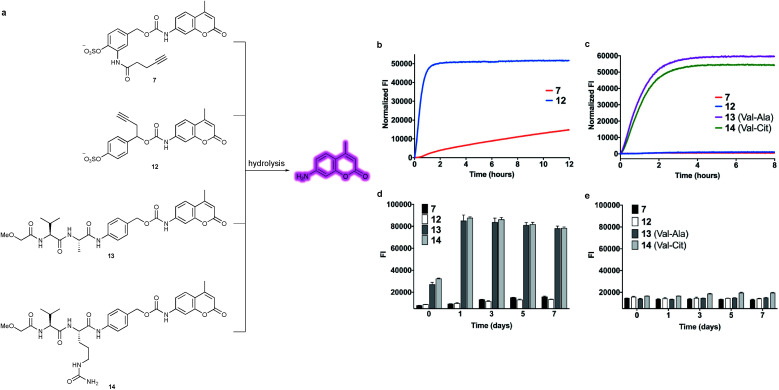
(a) Structures of model linker-AMC constructs including arylsulfates **7** and **12** and Val–Ala **13** and Val–Cit **14**. Upon hydrolysis and subsequent 1,6-elimination, the AMC payload is released and fluoresces. (b) Comparison of enzymolysis rates of **7***versus***12** when incubated with sulfatase from *Helix pomatia*. (c) Stability comparison of arylsulfates **7** and **12***versus* dipeptides **13** and **14** in mouse plasma over 8 hours. (d) Stability comparison in mouse plasma over 7 days. The *t* = 0 reading was taken after adding plasma to all the samples, by which time **13** and **14** were already partially hydrolysed. (e) Stability comparison in human plasma over 7 days.

With constructs **7** and **12** in hand, the lability of the linkers under conditions representative of the lysosomal compartments and blood plasma was evaluated (Fig. 4a). First, to approximate the lysosomal environment, the susceptibility of the arylsulfate linkers **7** and **12** to sulfatase cleavage was determined using sulfatase from *Helix pomatia* (EC 3.1.6.1). This enzyme has been used previously to approximate sulfate hydrolysis, given its general sulfatase activity.^28^ The arylsulfates were incubated with the sulfatase and the fluorescence of the released AMC payload was measured over 8 h (Fig. 4b). The resulting increase in fluorescence confirmed that both linkers are substrates for sulfatases and the subsequent 1,6-elimination occurs as expected. Although both linkers were hydrolysed by the sulfatase, the enzymolysis rate of benzyl-linked **12** (*t*_1/2_ = 24 min) was dramatically superior to that of *ortho*-amide linked **7** (*t*_1/2_ > 12 h) under the same conditions. These results suggest an ADC linker based on **12** would release its payload much more efficiently in the lysosome than **7**, increasing the cytotoxicity of the ADC.

To investigate the enzymatic nature of the hydrolysis, sulfates **7** and **12** were incubated with the sulfatase in the presence of phenyl sulfamate, a known sulfatase inhibitor (Fig. S3†).^29^ Pleasingly, no fluorescence was observed under these conditions, confirming that enzymolysis is required for release. Furthermore, incubation of **12** at pH 7.4 and pH 9 demonstrated the acidic optimum pH of the sulfatase hydrolysis, with the cleavage rate decreasing with increasing pH (Fig. S4†). Finally, to affirm its susceptibility towards human lysosomal sulfatases, **12** was incubated with recombinant human lysosomal arylsulfatase A (ARSA) and arylsulfatase B (ARSB), upon which, fluorophore release was observed in both cases (Fig. S5†).

The plasma stability of arylsulfate-AMC constructs **7** and **12** was then investigated, first by incubation in mouse plasma (Fig. 4c). Under these conditions, the sulfate linkers **7** and **12** were highly stable, with no significant increase in fluorescence. Contrastingly, dipeptides **13** and **14** were rapidly hydrolysed in mouse plasma, with half-lives of less than one hour. The exceptional stability of **7** and **12** was further confirmed by incubation in both human and mouse plasma over seven days, with minimal hydrolysis observed (Fig. 4d and e). Moreover, sulfates **7** and **12** were shown to be stable in the presence of an intracellular nucleophile (glutathione) (Fig. S6†).

### Design of linker-MMAE constructs

**Fig. 5:**
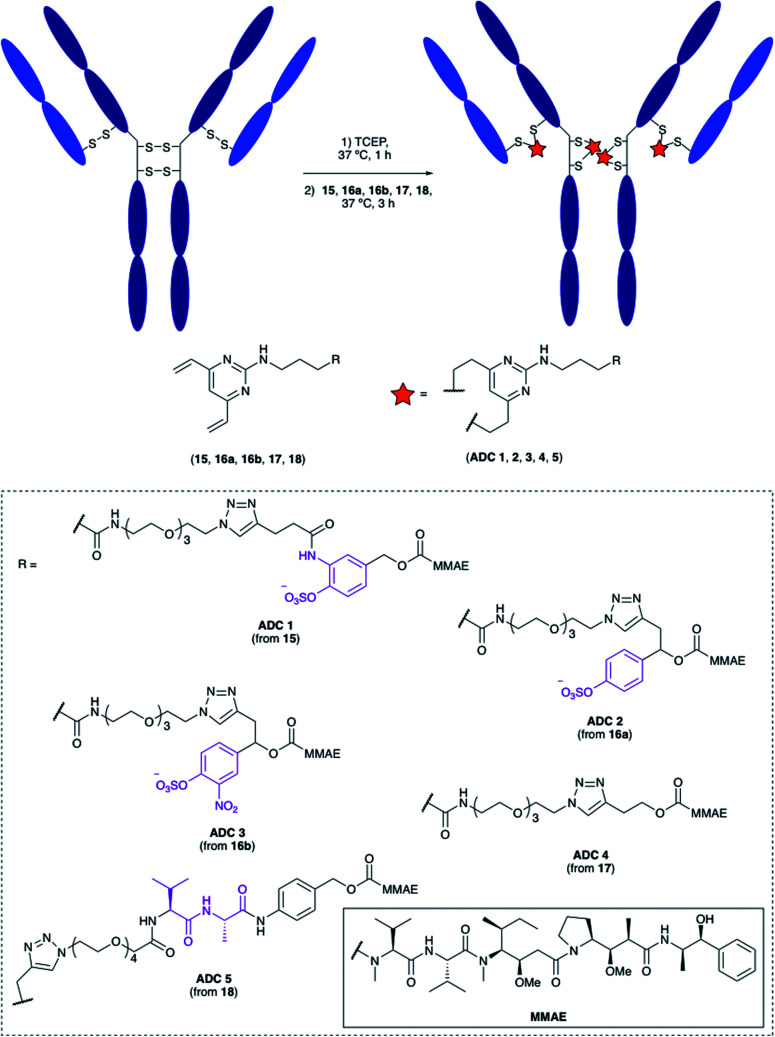
Bioconjugation of linker-payloads **15**, **16a**, **16b**, **17** and **18** to trastuzumab to afford **ADCs 1–5**.

Having confirmed the favourable properties of arylsulfates **7** and **12**, the linkers were elaborated to include an antibody attachment group and a cytotoxic payload, for biological evaluation of the proceeding ADCs. The divinylpyrimidine (DVP) group has been reported by our group as an effective method for creating stable, homogeneous ADCs with an average drug-antibody ratio (DAR) of four.^30^ We therefore included this functional handle, as well as the cytotoxic MMAE payload for the full linker-payloads. Linker-payloads **15** and **16a** are derived from the model compounds **7** and **12** respectively, and were expected to exhibit similar plasma stability and sensitivity towards sulfatases. Linker-payload **16b** is an analogue of **16a** with an added electron-withdrawing NO_2_ group on the arylsulfate, included because of the increased activity of sulfatases towards electron-poor arylsulfates.^31^ Non-cleavable analogue **17** and Val–Ala-containing linker payload **18** were also synthesised so that their biological properties could be compared to the arylsulfates *in vitro* (structures in Fig. 5, full synthetic details described in the ESI†).

### Bioconjugation

The linker-payloads were conjugated to trastuzumab, an FDA-approved IgG mAb which targets the HER2 receptor, an internalising antigen that is overexpressed in around 20% of breast cancers.^32^ Our earlier studies confirmed the excellent retention of antibody binding affinity and internalisation of DVP-derived trastuzumab ADCs, as well as the high plasma stability of the cysteine-DVP linkage.^30^ First, the four interchain disulfides were reduced by TCEP for 1 h to reveal eight reactive cysteine residues (Fig. 5). Then linker-payloads **15**, **16a**, **16b**, **17** and **18** were incubated for 3 h to react with the cysteine residues, rebridging them to form five ADCs (**ADCs 1–5**). Efficient conversion (>95%) to the desired conjugates was confirmed by LC-MS and SDS-PAGE analysis (Fig. S1 and S2†).

### *In vitro* cytotoxicity

**Fig. 6:**
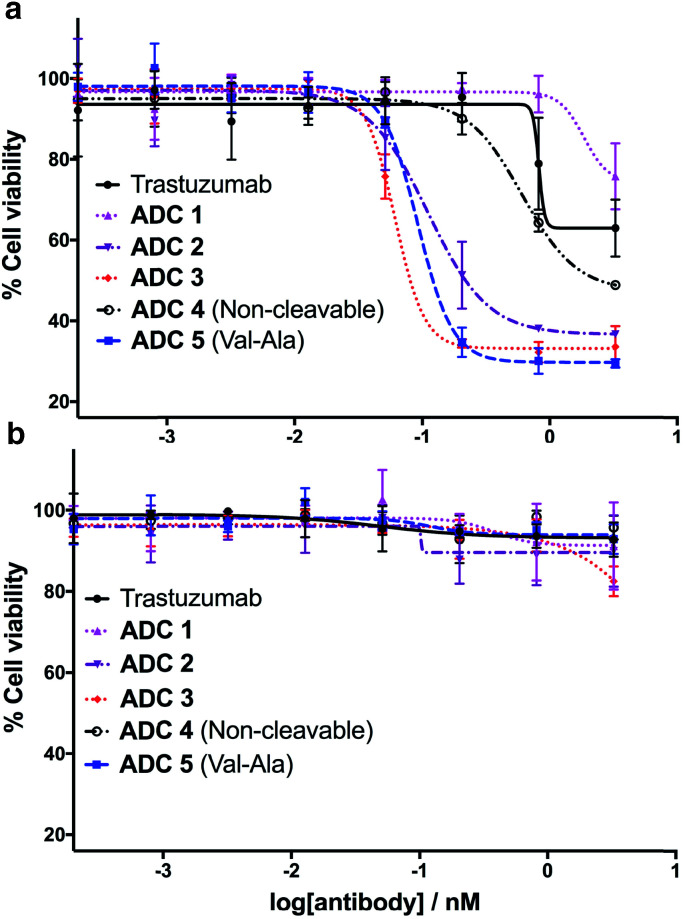
*In vitro* biological evaluation of **ADCs 1–5** in (a) HER2-positive BT474 cells and (b) HER2-negative MCF7 cells. Viability data shows the mean of three independent experiments and error bars represent standard error of the mean. For BT474 cells, IC_50_ values are as follows: **ADC 1** = N/A; **ADC 2** = 111 pM; **ADC 3** = 61 pM; **ADC 4** = 609 pM; **ADC 5** = 92 pM.

Having successfully synthesised the cleavable and non-cleavable ADCs (**ADCs 1–5**), their cytotoxicity against HER2-positive (BT474) and HER2-negative (MCF7) cells was evaluated (Fig. 6). As expected, the cathepsin-cleavable **ADC 5** demonstrated increased cytotoxicity (IC_50_ = 92 pM) towards the HER2-positive BT474 cells compared with the non-cleavable **ADC 4** (IC_50_ = 609 pM). This six-fold increase in potency can be explained by the traceless release of MMAE, compared with the modified MMAE-metabolite released from non-cleavable **ADC 4**, whose tubulin-binding activity is expected to be lower. Gratifyingly, arylsulfate-containing **ADC 2** and **ADC 3** compared similarly with the dipeptidic **ADC 5**; both were also 5–10 times more cytotoxic than non-cleavable **ADC 4** (Fig. 6a). The similar performance of these arylsulfate- and dipeptide-ADCs suggests that the arylsulfate linkers are also being cleaved in the cells following ADC internalisation. Contrastingly, **ADC 1** was non-toxic across both HER2-positive cell lines, demonstrating that *ortho*-amide containing arylsulfate linkers are unsuitable for ADCs. The complete lack of cytotoxicity indicates that the *in vitro* sulfatase cleavage of **ADC 1** is occurring at an insufficient rate and the maintained presence of the anionic sulfate group adjacent to the auristatin payload inhibits its tubulin-binding ability.

All five ADCs were non-toxic towards the HER2-negative MCF7 cells up to 3 nM, validating the stability of the linkers in media and the retained functionality of the mAb to bind and internalise with the HER2 antigen (Fig. 6b). Furthermore, trastuzumab's lack of cytotoxicity against HER2-positive BT474 cells confirms that the MMAE payload is critical to the cell-killing ability of the ADCs. The results of our *in vitro* evaluations were confirmed by testing the ADCs in additional HER2-positive (SKBR3) and HER2-negative (T47D) cell lines, where similar trends were observed (Fig. S7 and Table S1†).

## Conclusions

In conclusion, we have demonstrated the suitability of arylsulfates as cleavable linkers for ADCs. Model studies of the linkers with a fluorometric AMC payload validate their susceptibility towards sulfatase enzymes to tracelessly release amine-linked payloads, as well as their vastly superior mouse plasma stability compared to dipeptidic linkers. Upon conjugation to trastuzumab with an MMAE payload, all five ADCs retained selectivity towards HER2-positive cells. The arylsulfate-containing **ADC 2** and **ADC 3** were significantly more potent against HER2-positive cells than the non-cleavable **ADC 4**, suggesting the highly potent MMAE payload is being released inside the cells. The dramatic difference in cytotoxicity between **ADC 1** and **ADCs 2** and **3** reflects the difference in enzymolysis rates of **7** and **12**, suggesting that rapid payload release rates are crucial to the cell-killing capacity of the arylsulfate-ADCs. An appropriate choice of aryl-substitution is therefore vital to the cytotoxicity of sulfatase-cleavable linkers.

We envision that the arylsulfate linkers should be applicable to other lipophilic payloads, given their solubility and synthetic viability. Furthermore, the ubiquity of lysosomal sulfatases should enable pairing of the arylsulfate linkers with alternative antibodies for the treatment of a range of cancer types.

## Conflicts of interest

There are no conflicts to declare.

## Supplementary Material

SC-011-C9SC06410A-s001
