# Supplementary material for: Sulfatase-cleavable linkers for antibody-drug conjugates
Source: Chem Sci. 2020 Jan 27;11(9):2375–80. doi: 10.1039/c9sc06410a (PMC8157321; doi:10.1039/c9sc06410a)

Electronic Supplementary Material (ESI) for Chemical Science.
This journal is © The Royal Society of Chemistry 2020

Sulfatase-Cleavable Linkers for Antibody-Drug Conjugates

[1]Jonathan D. Bargh, [1,2]Stephen J. Walsh, [3]Albert Isidro-Llobet, [2]Soleilmane Omarjee, [2]Jason S. Carroll, [1]David R. Spring*

[1]Department of Chemistry, University of Cambridge, Lensfield Rd, Cambridge, CB2 1EW, UK.
[2]Cancer Research UK Cambridge Institute, University of Cambridge, Robinson Way, Cambridge, CB2 0RE, UK.
[3]GSK, Gunnels Wood Road, Stevenage SG1 2NY, UK.

Supplementary Information

Table of Contents

GENERAL EXPERIMENTAL DETAILS **3**

CHEMICAL SYNTHESIS **5**

BIOCONJUGATION **27**

LC-MS ANALYSIS 28

SDS-PAGE ANALYSIS 30

SULFATASE HYDROLYSIS **31**

SULFATASE FROM *HELIX POMATIA* WITH 7 AND 12 31

SULFATASE FROM *HELIX POMATIA* AND PHENYL SULFAMATE WITH 7 AND 12 31

SULFATASE FROM *HELIX POMATIA* WITH 12 AT PH 5, 7.4 AND 9 31

ARYLSULFATASE A (ARSA) AND ARYLSULFATASE B (ARSB) WITH 12 32

STABILITY STUDIES **33**

MOUSE PLASMA STABILITY OVER 8 HOURS 33

HUMAN/MOUSE PLASMA STABILITY OVER 7 DAYS 33

GLUTATHIONE STABILITY 33

CYTOTOXICITY OF ADCS 1-5 IN HER2-POSITIVE SKRB3 AND HER2-NEGATIVE T47D CELLS **34**

IC_{50} VALUES OF ADCS 1-5 IN HER2+ CELLS **34**

CELLS LINES **34**

CELL VIABILITY **34**

BIBLIOGRAPHY **36**

NMR SPECTRA AND HPLC TRACES **37**

General Experimental Details

All solvents and reagents were used as received unless otherwise stated. Ethyl acetate, methanol, dichloromethane, acetonitrile and toluene were distilled from calcium hydride. Diethyl ether was distilled from a mixture of lithium aluminium hydride and calcium hydride. Petroleum ether (PE) refers to the fraction between 40–60 °C upon distillation. Tetrahydrofuran (THF) was dried using Na wire and distilled from a mixture of lithium aluminium hydride and calcium hydride with triphenylmethane as indicator.

Non-aqueous reactions were conducted under a stream of dry nitrogen using oven-dried glassware. Temperatures of 0 °C were maintained using an ice-water bath. Room temperature (rt) refers to ambient temperature.

Yields refer to spectroscopically and chromatographically pure compounds unless otherwise stated. Reactions were monitored by thin layer chromatography (TLC) or liquid chromatography mass spectroscopy (LC-MS). TLC was performed using glass plates pre-coated with Merck silica gel 60 F254 and visualised by quenching of UV fluorescence (λ_{max} = 254 nm) or by staining with potassium permanganate. Retention factors (Rf) are quoted to 0.01.

Flash column chromatography was carried out using slurry-packed Merck 9385 Kieselgel 60 SiO_2 (230-400 mesh) or Combiflash Rf200 automated chromatography system with Redisep® normal-phase silica flash columns (35−70 µm) or Redisep® reverse-phase C18-silica flash columns (20-40 µm).

Analytical high performance liquid chromatography (HPLC) was performed on Agilent 1260 Infinity machine, using a Supelcosil™ ABZ+PLUS column (150 mm × 4.6 4 mm, 3 µm) with a linear gradient system (solvent A: 0.05% (v/v) TFA in H_2O; solvent B: 0.05% (v/v) TFA in MeCN) over 20 min at a flow rate of 1 mL/min, and UV detection (λ_{max} = 220 – 254 nm).

Infrared (IR) spectra were recorded neat on a Perkin-Elmer Spectrum One spectrometer with internal referencing. Selected absorption maxima (ν_{max}) are reported in wavenumbers (cm^{-1}).

Proton and carbon nuclear magnetic resonance (NMR) were recorded using an internal deuterium lock on Bruker DPX-400 (400 MHz, 101 MHz), Bruker Avance 400 QNP (400 MHz, 101 MHz), Bruker Avance 500 Cryo Ultrashield (500 MHz, 126 MHz) and 600 MHz Bruker Avance 600 BBI (600 MHz). Tetramethylsilane was used as an internal standard. In proton NMR, chemical shifts (δ_H) are reported in parts per million (ppm), to the nearest 0.01 ppm and are referenced to the residual non-deuterated solvent peak ($CDCl_3$: 7.26, DMSO-d6: 2.50, CD_3OD: 3.31, D_2O: 4.79). Coupling constants (*J*) are reported in Hertz (Hz) to the nearest 0.1 Hz. Data are reported as follows: chemical shift, multiplicity (s = singlet; d = doublet; t = triplet; q = quartet; qn = quintet; sep = septet; m = multiplet; br = broad or as a combination of these, e.g. dd, dt etc.), integration and coupling constant(s). In carbon NMR, chemical shifts (δ_C) are quoted in ppm, to the nearest 0.1 ppm, and are referenced to the residual non-deuterated solvent peak ($CDCl_3$: 77.16, DMSO-d6, 39.52, CD_3OD: 49.00).

High resolution mass spectrometry (HRMS) measurements were recorded with a Micromass Q-TOF mass spectrometer or a Waters LCT Premier Time of Flight mass spectrometer. Mass values are reported within the error limits of ±5 ppm mass units. ESI refers to the electrospray ionisation technique.

Protein LC–MS was performed on a Xevo G2-S TOF mass spectrometer coupled to an Acquity UPLC system using an Acquity UPLC BEH300 C4 column (1.7 µm, 2.1 × 50 5 mm). H_2O with 0.1% formic

acid (solvent A) and 95% MeCN and 5% water with 0.1% formic acid (solvent B), were used as the mobile phase at a flow rate of 0.2 mL/min. The gradient was programmed as follows: 95% A for 0.93 min, then a gradient to 100% B over 4.28 min, then 100% B for 1.04 minutes, then a gradient to 95% A over 1.04 min. The electrospray source was operated with a capillary voltage of 2.0 kV and a cone voltage of 40 V. Nitrogen was used as the desolvation gas at a total flow of 850 L/h. Total mass spectra were reconstructed from the ion series using the MaxEnt algorithm preinstalled on MassLynx software (v4.1 from Waters) according to the manufacturer’s instructions. Trastuzumab samples were deglycosylated with PNGase F (New England Biolabs) prior to LC-MS analysis.

Fluorescence was measured with a Pherastar FS plate reader using a 350/460 optic module.

Chemical Synthesis

Scheme S 1: Synthesis of linker-AMC 7.[a]

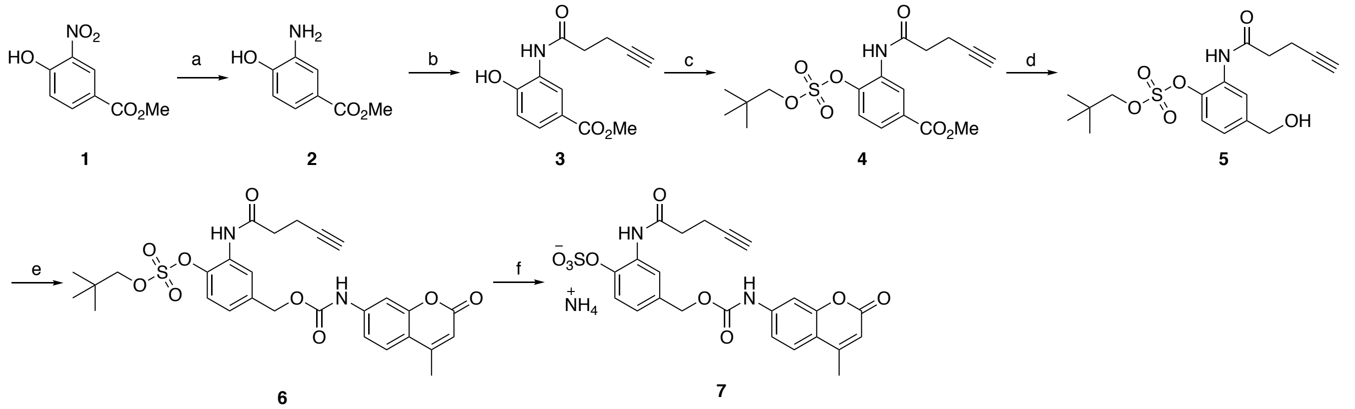

[a]Reagents and conditions: (a) 10% Pd/C, H_2, MeOH, rt, 1 h, 94%; (b) 4-pentynoic acid, HATU, Et_3N, CH_2Cl_2/DMF, 0 °C to rt, 30 min. Then $LiOH \cdot H_2O$, H_2O, MeOH, THF, rt, 2 h, 50%; (c) Neopentyl sulfochloridate, 4-DMAP, Et_3N, THF, 0 °C for 30 min then rt for 2 h, 81%; (d) $LiAlH_4$, THF, -60 °C for 16 h then -25 °C for 1 h, 80%; (e) AMC, triphosgene, toluene, reflux, 90 min then **5**, dibutyltin dilaurate, THF, rt, 15 h, 92%; (f) 5 M NH_4OAc (aq), DMF, 50 °C, 2 days, 54%.
Neopentyl sulfochloridate was synthesised and characterised in accordance with literature.[1]

Methyl 3-amino-4-hydroxybenzoate (2)

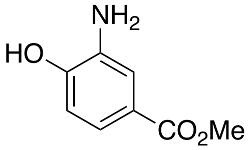

To a solution of methyl 4-hydroxy-3-nitrobenzoate (5.00 g, 25.4 mmol) in MeOH (130 mL) was added 10% Pd/C (2.70 g, 2.54 mmol) before stirring vigorously under H_2 atmosphere (balloon) at rt for 1 h. The reaction mixture was filtered through Celite®, washing with MeOH and the filtrate was concentrated *in vacuo* to yield methyl 3-amino-4-hydroxybenzoate **2** (3.99 g, 23.9 mmol, 94%) as a yellow solid. **Rf** 0.49 (100% EtOAc); **^{1}H NMR** (400 MHz, CD_3OD): δ 7.39 (d, 1H, *J* = 2.1 Hz), 7.30 (dd, 1H, *J* = 8.2, 2.1 Hz), 6.72 (d, 1H, *J* = 8.2 Hz), 3.82 (s, 3H); **^{13}C NMR** (101 MHz, CD_3OD): δ 169.2, 151.3, 136.6, 122.6, 122.4, 117.8, 114.7, 52.2. Data in accordance with literature.[2]

Methyl 4-hydroxy-3-(pent-4-ynamido)benzoate (3)

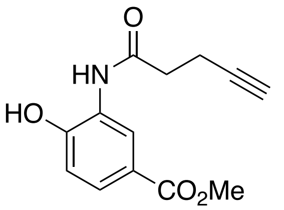

To a stirred solution of methyl 3-amino-4-hydroxybenzoate **2** (400 mg, 2.39 mmol), 4-pentynoic acid (258 mg, 2.63 mmol) and HATU (1.00 g, 2.63 mmol) in DMF (10 mL) and CH_2Cl_2 (10 mL) was added triethylamine (0.667 mL, 4.79 mmol) at 0 °C. After 5 min the solution was warmed to rt and stirred for 30 min. The reaction mixture was concentrated *in vacuo,* diluted with EtOAc (40 mL) and washed with 1 M HCl (aq) (20 mL). The organic fraction was dried ($MgSO_4$), concentrated *in vacuo* and purified by flash column chromatography (25-40% EtOAc in PE). The residue was dissolved in

THF (1 mL), MeOH (1 mL) and H_2O (1 mL) and lithium hydroxide monohydrate (110 mg, 2.63 mmol) was added before stirring at rt for 2 h. The mixture was diluted with EtOAc (20 mL) and washed with 1 M HCl (aq) (10 mL) and H_2O (10 mL), before being dried ($MgSO_4$) and concentrated *in vacuo* to yield methyl 4-hydroxy-3-(pent-4-ynamido)benzoate **3** (305 mg, 1.23 mmol, 51%) as a white solid. **Rf** 0.25 (50% EtOAc in PE); $\mathbf{\nu_{max}}$ (neat/cm^{1}) 3285 (br), 1709 (s), 1673 (m), 1594 (m), 1549 (s), 1447 (s); **^{1}H NMR** (400 MHz, DMSO-d_6) δ 10.79 (s, 1H), 9.33 (s, 1H), 8.53 (d, 1H, *J* = 2.0 Hz), 7.58 (dd, 1H, *J* = 8.4, 2.2 Hz), 6.94 (d, 1H *J* = 8.4 Hz), 2.79 (t, 1H, *J* = 2.6 Hz), 2.63 (t, 2H, *J* = 7.2 Hz), 2.44 (td, 2H, *J* = 2.6, 7.2 Hz); **^{13}C NMR** (101 MHz, DMSO-d_6): δ 170.0, 166.0, 152.1, 126.22, 126.17, 123.2, 120.1, 115.0, 83.7, 71.4, 51.7, 34.7, 14.1; **HRMS** (ESI) *m/z* found $[M+H]^+$, 248.0921 $C_{13}H_{14}NO_4^+$ required 248.0917.

Methyl 4-(((4-nitrophenoxy)sulfonyl)oxy)-3-(pent-4-ynamido)benzoate (4)

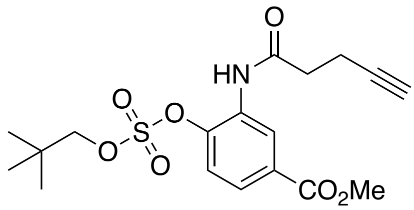

Neopentyl sulfurochloridate (0.386 mL, 2.43 mmol) was added to a solution of methyl 4-hydroxy-3-(pent-4-ynamido)benzoate **3** (300 mg, 1.21 mmol), 4-DMAP (148 mg, 1.21 mmol) and triethylamine (0.338 mL, 2.43 mmol) in THF (20 mL) at 0 °C. After 30 min, the reaction mixture was warmed to rt and stirred for 2 h. The reaction mixture was concentrated *in vacuo*, diluted with EtOAc (30 mL) and washed with 1 M HCl (aq) (30 mL). The organic fraction was dried ($MgSO_4$), concentrated *in vacuo* and purified by flash column chromatography (15-20% EtOAc in PE) to yield methyl 4-(((4-nitrophenoxy)sulfonyl)oxy)-3-(pent-4-ynamido)benzoate **4** (392 mg, 0.986 mmol, 81%) as a colourless oil. **Rf** 0.61 (50% EtOAc in PE); $\mathbf{\nu_{max}}$ (neat/cm^{1}) 2981 (w), 1715 (s), 1651 (m), 1601 (w), 1509 (m), 1491 (w); **^{1}H NMR** (600 MHz, DMSO-d_6) δ 9.98 (s, 1H), 8.49 (d, 1H, *J* = 1.5 Hz), 7.84 (dd, 1H, *J* = 8.6, 2.2, Hz), 7.62 (d, 1H, *J* = 8.6 Hz), 4.23 (s, 2H), 3.87 (s, 3H), 2.81 (t, 1H, *J* = 2.6 Hz), 2.63 (t, 2H, *J* = 7.3 Hz), 2.46 (td, 2H, *J* = 10.9, 2.5 Hz), 0.92 (s, 9H); **^{13}C NMR** (101 MHz, DMSO-d_6) δ 170.0, 165.1, 144.3, 130.4, 128.6, 126.3, 126.0, 121.4, 84.1, 83.4, 71.5, 52.5, 34.7, 31.6, 25.4, 13.9; **HRMS** (ESI) *m/z* found $[M+H]^+$ 398.1276, $C_{18}H_{24}NO_7S^+$ required 398.1268.

4-(hydroxymethyl)-2-(pent-4-ynamido)phenyl neopentyl sulfate (5)

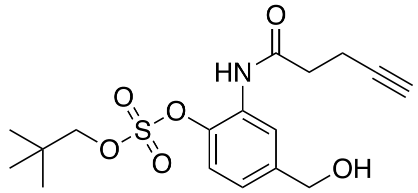

A solution of methyl 4-(((4-nitrophenoxy)sulfonyl)oxy)-3-(pent-4-ynamido)benzoate **4** (200 mg, 0.503 mmol) in THF (2.5 mL) was added to a suspension of $LiAlH_4$ (57.3 mg, 1.51 mmol) in THF (2.5 mL) at -60 °C and stirred for 16 h. The reaction mixture was warmed to -25 °C and stirred for 1 h before diluting with wet ether (5 mL) and adding H_2O (0.1 mL) then 15 wt% NaOH (0.1 mL) at 0 °C. Additional H_2O (0.2 mL) was then added before warming to rt and stirring for 15 min. $MgSO_4$ was then added before stirring at rt for 15 min and filtering through Celite®. The filtrate was concentrated *in vacuo* and purified by flash column chromatography (50% EtOAc in PE) to yield 4-(hydroxymethyl)-2-(pent-4-ynamido)phenyl neopentyl sulfate **5** (149 mg, 0.403 mmol, 80%) as a colourless oil. **Rf** 0.24 (50% EtOAc in PE); $\mathbf{\nu_{max}}$ (neat/cm^{1}) 3297 (w), 2964 (w), 1676 (m), 1605 (m), 1537 (m), 1477 (m), 1388 (s); **^{1}H NMR** (600 MHz, $CDCl_3$) δ 8.33 (s, 1H), 7.87 (s, 1H), 7.33 (d, 1H, *J* = 8.4 Hz), 7.19 (dd, 1H, *J* = 8.5, 1.8 Hz), 4.70 (s, 2H), 4.11 (s, 2H), 2.66 (m, 2H), 2.62 (m, 2H), 2.10 (t,

1H, *J* = 2.5 Hz), 1.02 (s, 9H); **^{13}C NMR** (101 MHz, $CDCl_3$) δ 169.7, 141.3, 138.7, 130.4, 123.1, 121.6, 121.4, 84.7, 82.5, 70.2, 64.6, 36.6, 32.2, 26.0, 14.8; **HRMS** (ESI) *m/z* found $[M+H]^+$ 370.1329, $C_{17}H_{24}NO_6S^+$ required 370.1319.

4-((((4-methyl-2-oxo-2*H*-chromen-7-yl)carbamoyl)oxy)methyl)-2-(pent-4-ynamido)phenyl (4-nitrophenyl) sulfate (6)

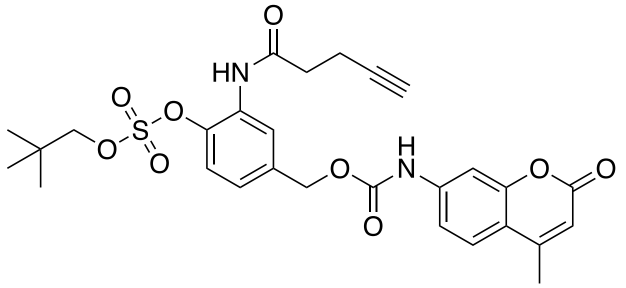

7-Amino-4-methylcoumarin (23.6 mg, 0.135 mmol) and triphosgene (20.0 mg, 0.0674 mmol) were suspended in toluene (2.6 mL) and refluxed for 90 min. The reaction mixture was cooled and evaporated under a stream of nitrogen before a solution of 4-(hydroxymethyl)-2-(pent-4-ynamido)phenyl neopentyl sulfate **5** (50.0 mg, 0.135 mmol) in THF (2.6 mL) was added. Dibutyltin dilaurate (8.0 µL, 13.5 µmol) was added to the resulting suspension and stirred for 15 h before being quenched with H_2O (0.5 mL) and evaporated under a stream of nitrogen. The crude residue was washed with H_2O (3 x 10 mL) and dried *in vacuo* to yield **6** (71.0 mg, 0.124 mmol, 92%) as a beige solid. **Rf** 0.48 (50% EtOAc in PE); **$ν_{max}$** (neat/cm^{1}) 3258 (w), 2955 (w), 1700 (s), 1688 (s), 1618 (m), 1585 (m), 1535 (m); **^{1}H NMR** (400 MHz, DMSO-*d_6*) δ 10.33 (s, 1H), 9.85 (s, 1H), 7.90 (s, 1H), 7.70 (d, 1H, *J* = 8.7 Hz), 7.55 (d, 1H, *J* = 1.8 Hz), 7.48 (d, 1H, *J* = 8.5 Hz), 7.41 (dd, 1H, *J* = 8.7, 2.0 Hz), 7.34 (dd, 1H, *J* = 8.5, 1.7 Hz), 6.24 (d, 1H, *J* = 1.1 Hz), 5.20 (s, 2H), 4.21 (s, 2H), 2.81 (t, 1H, *J* = 2.5 Hz), 2.59 (t, 1H, *J* = 7.2 Hz), 2.43 (td, 2H, *J* = 7.3, 2.1 Hz), 2.39 (s, 3H), 0.93 (s, 9H); **^{13}C NMR** (101 MHz, DMSO-*d_6*) δ 169.7, 160.0, 153.8, 153.2, 153.0, 142.6, 141.3, 135.9, 130.4, 126.1, 125.3, 125.2, 121.5, 114.5, 114.3, 112.0, 104.5, 83.7, 83.5, 71.6, 65.4, 34.6, 31.6, 25.4, 18.0, 14.0; **HRMS** (ESI) *m/z* found $[M+H]^+$ 571.1749, $C_{28}H_{31}N_2O_9S^+$ required 571.1750.

Ammonium 4-((((4-methyl-2-oxo-2*H*-chromen-7-yl)carbamoyl)oxy)methyl)-2-(pent-4-ynamido)phenyl sulfate (7)

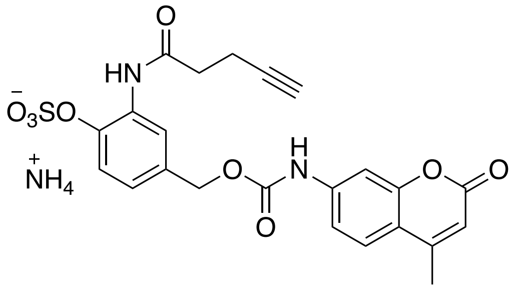

Coumarin **6** (65.5 mg, 127 µmol) was dissolved in DMF (0.8 mL) and treated with 5 M NH_4OAc (aq) (0.8 mL) before stirring at 50 °C for 2 days. The cooled reaction mixture was purified by reverse phase flash column chromatography (10-20% solvent B in solvent A. Solvent A: 0.1 M NH_4OH (aq). Solvent B: MeCN) and lyophilised to yield sulfate **7** (35.5 mg, 68.6 µmol, 54%) as a white solid. **$ν_{max}$** (neat/cm^{1}) 3267 (w), 3077 (w), 1724 (m), 1672 (m), 1578 (s), 1533 (s); **^{1}H NMR** (600 MHz, DMSO-*d_6*) δ 10.28 (s, 1H), 9.29 (s, 1H), 8.14 (s, 1H), 7.69 (d, 1H, *J* = 8.6 Hz), 7.55 (s, 1H), 7.42 (d, 1H, *J* = 9.1 Hz), 7.23 (d, 1H, *J* = 7.9 Hz), 7.11 (m, 5H), 6.23 (s, 1H), 5.12 (s, 2H), 2.79 (s, 1H), 2.53 (m, exp 2H), 2.46 (m, 2H), 2.39 (s, 3H); **^{13}C NMR** (101 MHz, DMSO-*d_6*) δ 168.9, 160.1, 153.8, 153.2, 153.1, 142.84, 142.77, 131.9, 131.2, 126.1, 124.0, 123.0, 121.7, 114.4, 114.3, 111.9, 104.4, 83.4, 71.6, 66.2, 35.5, 18.0, 14.0; **HRMS** (ESI) *m/z* found $[M-H^+]^-$ 499.0807, $C_{23}H_{19}N_2O_9S^-$ required 499.0817.

Scheme S 2: Synthesis of linker-AMC 12.[a]

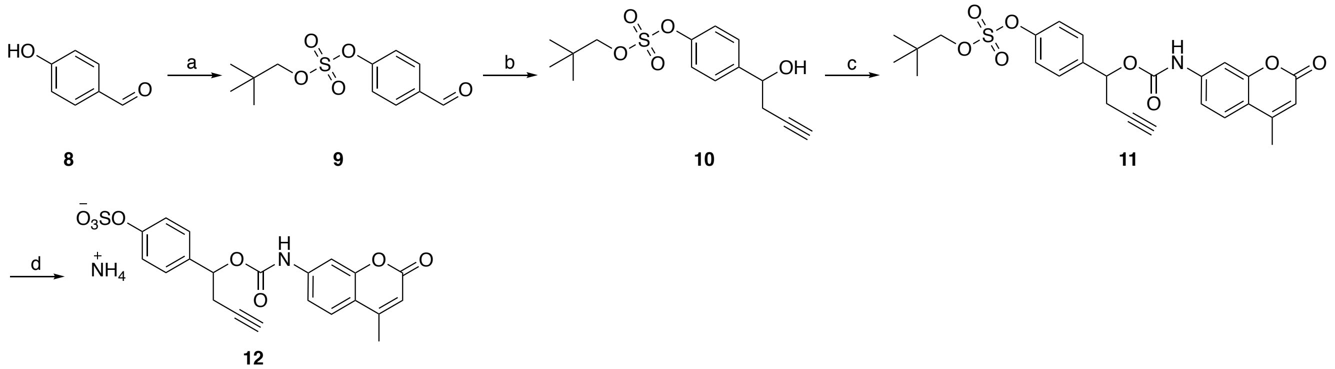

[a]Reagents and conditions: (a) Neopentyl sulfochloridate, 4-DMAP, Et_3N, THF, rt, 17 h, 66%; (b) Activated zinc, propargyl bromide, DMF, -10 °C to rt over 15 h, 74%; (c) AMC, triphosgene, toluene, reflux, 2 h then **10**, dibutyltin dilaurate, THF, rt, 3 h, 50%; (d) 5 M NH_4OAc (aq), DMF, 50 °C, 3 days, 81%.

4-formylphenyl neopentyl sulfate (9)

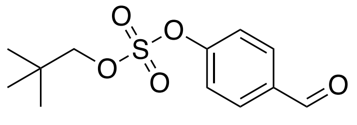

Neopentyl sulfurochloridate (1.56 mL, 9.80 mmol) was added dropwise to a solution of 4-hydroxybenzaldehyde (1.00 g, 8.19 mmol), 4-DMAP (1.00 g, 8.18 mmol) and triethylamine (2.28 mL, 16.4 mmol) in THF (16 mL) at rt. After 17 h the reaction mixture was concentrated *in vacuo*, diluted with EtOAc (30 mL) and washed with 1 M HCl (aq) (30 mL). The organic fraction was dried ($MgSO_4$), concentrated *in vacuo* and purified by flash column chromatography (10% EtOAc in PE) to yield 4-formylphenyl neopentyl sulfate **9** (1.76 g, 6.46 mmol, 66%) as a colourless oil. **Rf** 0.40 (25% EtOAc in PE); ν_{max} (neat/cm^1) 2964 (w), 2435 (w), 1683 (m), 1651 (m), 1602 (m), 1584 (m), 1516 (w), 1448 (w); **^{1}H NMR** (500 MHz, DMSO-d_6): δ 10.02 (s, 1H), 8.06 (s, 2H), 7.65 (s, 2H), 4.27 (s, 2H), 0.93 (s, 9H); **^{13}C NMR** (126 MHz, DMSO-d_6): δ 191.9, 153.5, 135.1, 131.7, 122.0, 83.9, 31.6, 25.4; **HRMS** (ESI) *m/z* found $[M+H]^+$ $C_{12}H_{17}O_5S$, 273.0804$^+$ required 273.0797$^+$.

4-(1-hydroxybut-3-yn-1-yl)phenyl neopentyl sulfate (10)

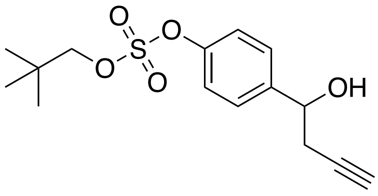

Zinc powder was activated by stirring with 1 M HCl (aq), washing with H_2O, EtOH, and ether before rigorous drying.

To a solution of 4-formylphenyl neopentyl sulfate **9** (1.00 g, 3.67 mmol) and propargyl bromide (80 wt% in toluene) (0.614 mL, 5.51 mmol) in DMF (15 mL) was added the activated zinc powder (360 mg, 5.51 mmol) at -10 °C. The reaction mixture was allowed to warm to rt over 15 h before quenching with sat. NH_4Cl (aq) (30 mL) and extracting with EtOAc (3 x 30 mL). The combined organic fractions were dried ($MgSO_4$), concentrated *in vacuo* and purified by flash column chromatography (10-15% EtOAc in PE) to yield 4-(1-hydroxybut-3-yn-1-yl)phenyl neopentyl sulfate **10** (849 mg, 2.72 mmol), 74%) as a yellow oil. **Rf** 0.28 (15% EtOAc in PE); ν_{max} (neat/cm^1) 3300 (br), 2967 (w), 1604 (w), 1504 (w), 1479 (w), 1389 (m), 1370 (m), 1205 (s); **^{1}H NMR** (400 MHz, $CDCl_3$): δ

7.45 (m, 2H), 7.30 (m, 2H), 4.90 (m, 1H), 4.08 (s, 2H), 2.63 (m, 2H), 2.42 (br d, 1H, *J* = 3.4 Hz), 2.09 (t, 1H, *J* = 2.6 Hz), 1.00 (s, 9H); **^{13}C NMR** (101 MHz, $CDCl_3$) δ 149.9, 141.7, 127.5, 121.2, 83.6, 80.2, 71.65, 71.61, 32.1, 29.7, 26.1; **HRMS** (ESI) *m/z* found $[M+H]^+$ 335.0933 $C_{15}H_{20}O_5SNa^+$, required 335.0929.

4-(1-(((4-methyl-2-oxo-2*H*-chromen-7-yl)carbamoyl)oxy)but-3-yn-1-yl)phenyl neopentyl sulfate (11)

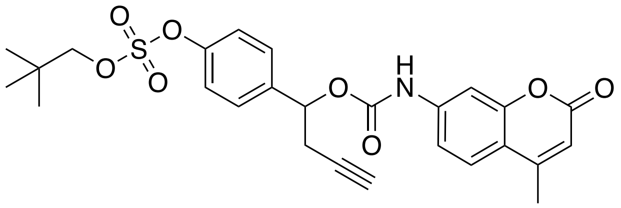

7-Amino-4-methylcoumarin (59.0 mg, 337 μmol) and triphosgene (50.0 mg, 168 μmol) were suspended in toluene (12 mL) and refluxed for 2 h. The reaction mixture was cooled and evaporated under a stream of nitrogen before a solution of alcohol **10** (115 mg, 368 μmol) in THF (12 mL) was added. Dibutyltin dilaurate (22 μL, 37 μmol) was added to the resulting suspension and stirred at rt for 3 h before being quenched with H_2O (1 mL) and concentrated *in vacuo*. The crude residue was washed with H_2O (10 mL), MeOH (4 x 10 mL) and MeCN (5 mL) to yield coumarin **11** (86.4 mg, 168 μmol, 50%) as a white solid. **Rf** 0.63 (50% EtOAc in PE); **ν_{max}** (neat/cm^1) 3291 (w), 2923 (m), 1729 (s), 1687 (s), 1618 (m), 1588 (m), 1535 (w); **^{1}H NMR** (400 MHz, DMSO-*d$_6$*) δ 10.42 (s, 1H), 7.69 (d, 1H, *J* = 8.7 Hz), 7.61 (d, 2H, *J* = 8.8 Hz), 7.53 (d, 1H, *J* = 1.8 Hz), 7.44 (m, 3H), 6.24 (s, 1H), 5.88 (t, 1H, *J* = 6.2 Hz), 4.23 (s, 2H), 2.92 (m, 1H), 2.88 (m, 2H), 2.38 (s, 3H), 0.93 (s, 9H); **^{13}C NMR** (101 MHz, DMSO-*d$_6$*) δ 160.0, 153.8, 153.2, 152.3, 149.3, 142.5, 138.8, 128.3, 126.1, 121.3, 114.6, 114.4, 112.1, 104.6, 83.5, 79.9, 73.8, 73.1, 31.6, 25.5, 18.0; **HRMS** (ESI) *m/z* found $[M+H]^+$ 514.1547 $C_{26}H_{28}NO_8S^+$, required 514.1530.

Ammonium 4-(1-(((4-methyl-2-oxo-2*H*-chromen-7-yl)carbamoyl)oxy)but-3-yn-1-yl)phenyl sulfate (12)

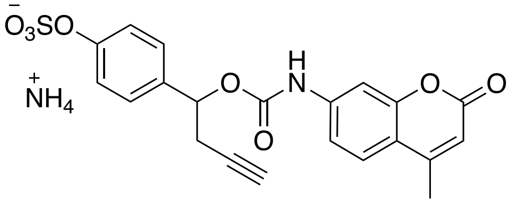

Coumarin **11** (85.0 mg, 165 μmol) was dissolved in DMF (1.5 mL) and treated with 5 M NH_4OAc (aq) (1 mL) before stirring at 50 °C for 3 days. After cooling, the reaction mixture was purified by reverse phase flash column chromatography (20% solvent B in solvent A. Solvent A: 50 mM NH_4OAc (aq). Solvent B: MeCN) and lyophilised to yield sulfate **12** (61.3 mg, 133 μmol, 81%) as a white solid. **Rf** ; **ν_{max}** (neat/cm^1) 3266 (w), 1726 (m), 1688 (s), 1616 (m), 1583 (w), 1530 (w), 1507 (w), 1207 (s); **^{1}H NMR** (500 MHz, DMSO-*d$_6$*) δ 10.34 (br, 1H), 7.68 (d, 1H, *J* = 8.7 Hz), 7.52 (d, 1H, *J* = 2.0 Hz), 7.44 (dd, 1H, *J* = 8.7, 2.1 Hz), 7.37 (d, 2H, *J* = 8.6 Hz), 7.18 (d, 2H, *J* = 8.6 Hz), 6.23 (d, 1H, *J* = 1.1 Hz), 5.79 (t, 1H, *J* = 6.5 Hz), 2.89 (t, 1H, *J* = 2.5 Hz), 2.84 (t, 2H, *J* = 3.1 Hz), 2.38 (m, 3H); **^{13}C NMR** (126 MHz, DMSO-*d$_6$*) δ 160.1, 153.8, 153.5, 153.2, 152.5, 142.6, 133.6, 127.1, 126.1, 120.3, 114.5, 114.4, 112.0, 104.6, 80.3, 73.7, 73.4, 25.6, 18.0; **HRMS** (ESI) *m/z* found $[M-H^+]^-$ 442.0583 $C_{21}H_{16}NO_8S^-$, required 442.0602.

Scheme S 3 : Synthesis of linker-payload 15.[a]

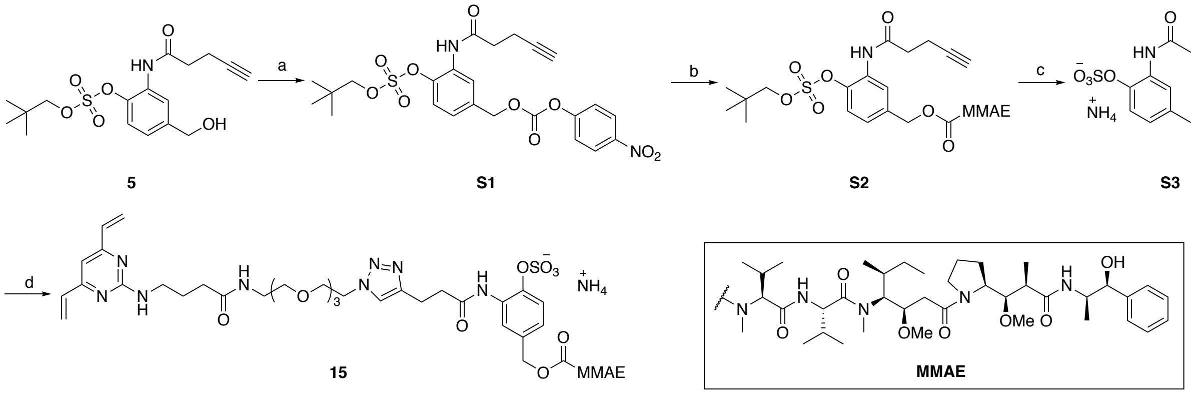

[a]Reagents and conditions: (a) 4-nitrophenyl chloroformate, pyridine, CH_2Cl_2, rt, 2 h, 90%; (b) MMAE, HOBt, pyridine, DIPEA, DMF, rt, 4 h, 89%; (c) 5 M NH_4OAc (aq), DMF, 50 °C, 2 days, 85%; (d) **21**, $CuSO_4 \cdot 5H_2O$, THPTA, sodium ascorbate, $H_2O/^{t}BuOH$, rt, 30 min, 69%.

Neopentyl (4-((((4-nitrophenoxy)carbonyl)oxy)methyl)-2-(pent-4-ynamido)phenyl) sulfate (S1)

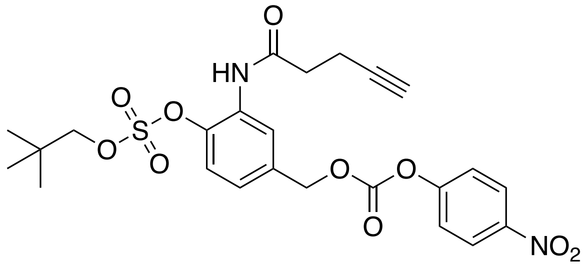

Pyridine (21.8 µL, 271 µmol) was added dropwise to a solution of 4-(hydroxymethyl)-2-(pent-4-ynamido)phenyl neopentyl sulfate **5** (20.0 mg, 54.1 µmol) and 4-nitrophenylchloroformate (32.1 mg, 108.3 µmol) in CH_2Cl_2 at rt and stirred for 2 h. The reaction mixture was diluted with EtOAc (20 mL) and washed with 1 M HCl (aq) (20 mL) then H_2O (20 mL). The organic fraction was dried ($MgSO_4$), concentrated *in vacuo* and purified by flash column chromatography (10-30% EtOAc in PE) to yield neopentyl (4-((((4-nitrophenoxy)carbonyl)oxy)methyl)-2-(pent-4-ynamido)phenyl) sulfate **S1** (26.0 mg, 48.6 µmol, 90%) as a colourless oil. **Rf** 0.32 (20% EtOAc in PE); $\mathbf{\nu_{max}}$ (neat/cm^{1}) 2963 (w), 1765 (m), 1695 (w), 1524 (m), 1559 (s), 1203 (s); ^{1}**H NMR** (600 MHz, $CDCl_3$) δ 8.51 (s, 1H), 8.28 (d, 2H, *J* = 8.9 Hz), 7.92 (br s, 1H), 7.40 (d, 2H, *J* = 9.1 Hz), 7.38 (d, 1H, *J* = 8.5 Hz), 7.22 (dd, 1H, *J* = 8.4, 1.9 Hz), 5.28 (s, 2H), 4.13 (s, 2H), 2.68 (m, 2H), 2.63 (m, 2H), 2.12 (t, 1H, *J* = 2.5 Hz), 1.03 (s, 9H); ^{13}**C NMR** (101 MHz, $CDCl_3$) δ 169.7, 155.6, 152.5, 150.3, 144.4, 134.5, 130.9, 125.5, 124.5, 122.9, 122.0, 121.8, 84.9, 82.4, 70.4, 70.0, 36.7, 32.2, 26.0 14.7; **HRMS** (ESI) *m/z* found $[M+H]^+$ 535.1379, $C_{24}H_{27}N_2O_{10}S^+$ required 535.1381.

Neopentyl arylsulfate-2-amide-MMAE (**S2**)

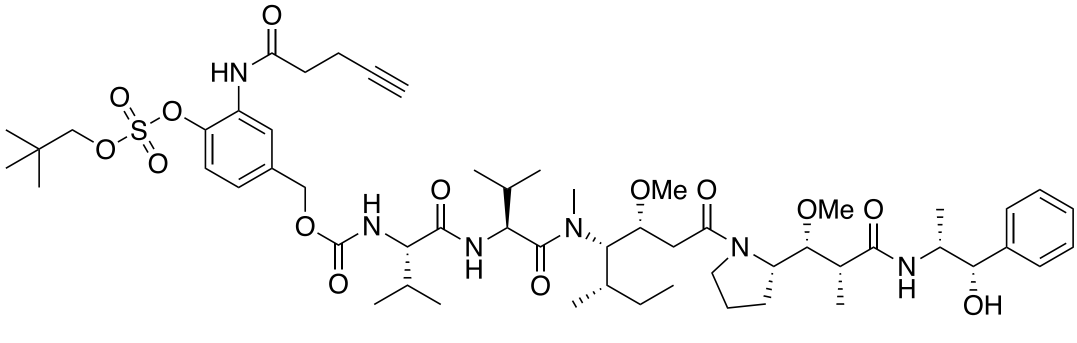

HOBt (80%) (2.76 mg, 16.3 µmol) was added to a solution of MMAE (23.5 mg, 32.7 µmol), neopentyl (4-((((4-nitrophenoxy)carbonyl)oxy)methyl)-2-(pent-4-ynamido)phenyl) sulfate **S1** (21.0

mg, 39.3 µmol), pyridine (92.3 µL, 1.15 mmol) and DIPEA (5.82 µL, 39.3 µmol) in DMF (0.3 mL) at rt and stirred for 4 h. The reaction mixture was diluted with EtOAc (20 mL) and washed with 1 M HCl (aq) (10 mL) and 1 M Na_2CO_3 (aq) (2 x 10 mL). The organic fraction was dried ($MgSO_4$), concentrated *in vacuo* and purified by flash column chromatography (0-5% MeOH in CH_2Cl_2) to yield carbamate **S2** (32.5 mg, 29.2 µmol, 89%) as a pale yellow oil. **Rf** 0.30 (5% MeOH in CH_2Cl_2); **HRMS** (ESI) *m/z* found $[M+H]^+$ 1113.6158, $C_{57}H_{89}N_6O_{14}S^+$ required 1113.6152; **HPLC** (5-95% MeCN/H_2O over 20 min) retention time 13.976 min.

Ammonium arylsulfate-2-amide-MMAE (**S3**)

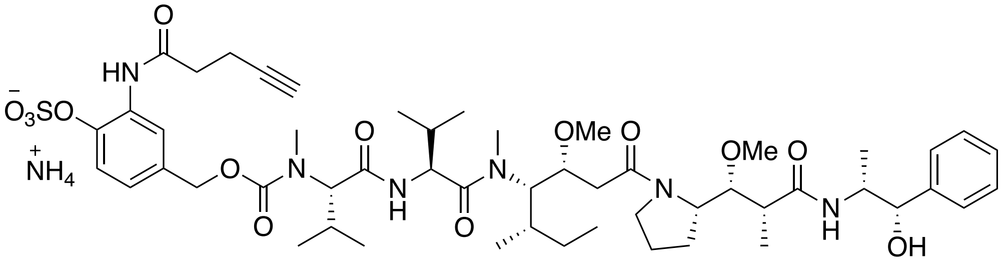

Carbamate **S2** (32.2 mg, 28.7 µmol) was dissolved in DMF (0.7 mL) and treated with 5 M NH_4OAc (aq) (0.5 mL) before stirring at 50 °C for 2 days. The cooled reaction mixture was purified by reverse phase flash column chromatography (20-50% solvent B in solvent A. Solvent A: 0.1 M NH_4OH (aq). Solvent B: MeCN) and lyophilised to yield sulfate **S3** (26.0 mg, 24.5 µmol, 85%) as a white solid. **HRMS** (ESI) *m/z* found $[M-H^+]^-$ 1041.5200, $C_{52}H_{77}N_6O_{14}S^-$ required 1041.5218; **HPLC** (5-95% MeCN/H_2O over 20 min) retention time 11.701 min.

Ammonium DVP-PEG$_3$-arylsulfate-2-amide-MMAE (**15**)

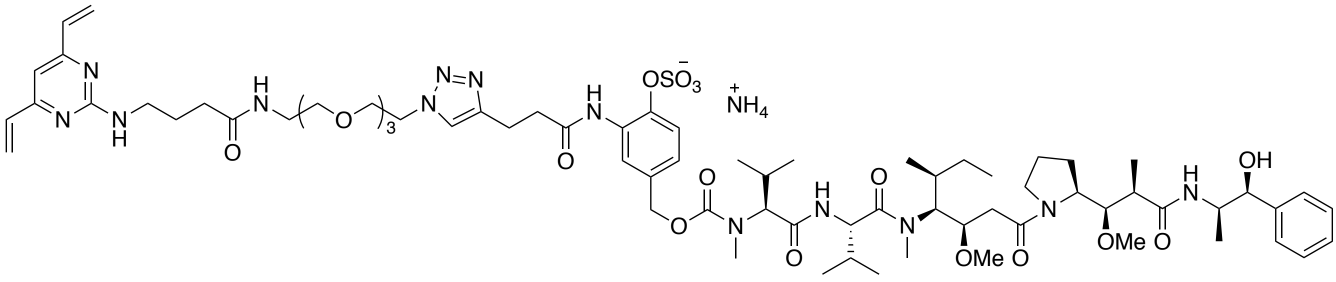

To a degassed solution of sulfate **S3** (10.0 mg, 9.43 µmol) and DVP **S16** (113 µL of 0.1 M solution in DMSO, 11.3 µmol) in ^{t}BuOH (0.25 mL) was added a degassed solution of $CuSO_4{\cdot}5H_2O$ (1.18 mg, 4.72 µmol), THPTA (4.10 mg, 9.43 µmol) and sodium ascorbate (3.74 mg, 18.9 µmol) in $H_2O/^tBuOH$ (0.5 mL, 1:1) and the reaction mixture was stirred at rt for 30 min. The reaction mixture was purified by reverse phase flash column chromatography (20-45% solvent B in solvent A. Solvent A: 0.1 M NH_4OH (aq). Solvent B: MeCN) and lyophilised to yield linker-drug **15** (9.60 mg, 6.49 µmol, 69%) as a pale yellow solid. **HRMS** (ESI) *m/z* found $[M-H^+]$ 1474.7609, $C_{72}H_{108}N_{13}O_{18}S^-$ required 1474.7656; **HPLC** (5-95% MeCN/H_2O over 20 min) retention time 10.088 min.

Scheme S 4 : Synthesis of linker-payload 16a.[a]

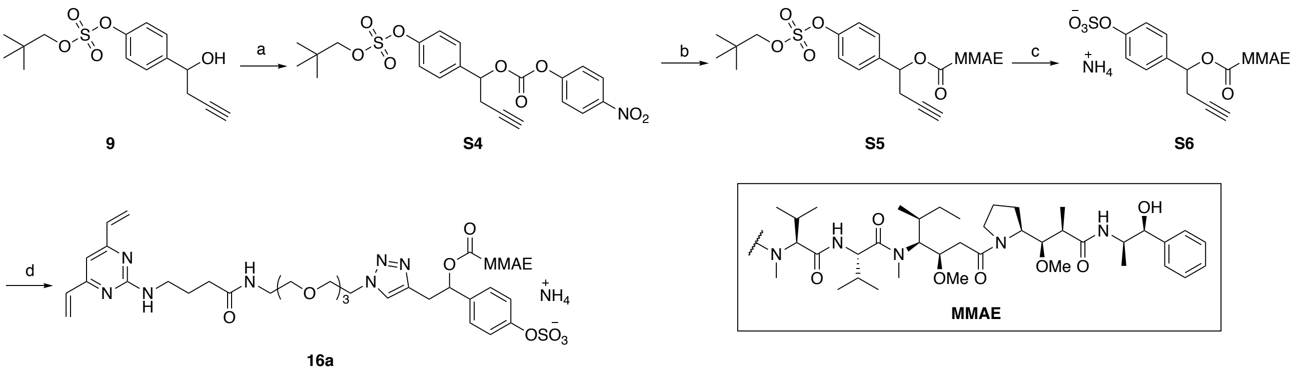

[a]Reagents and conditions: a) 4-nitrophenyl chloroformate, Et_3N, THF, rt, 24 h, 62%; (b) MMAE, HOBt, pyridine, DIPEA, DMF, rt, 48 h, 82%; (c) 5 M NH_4OAc (aq), DMF, 50 °C, 4 days, 80%; (d) **21**, $CuSO_4 \cdot 5H_2O$, THPTA, sodium ascorbate, $H_2O/^tBuOH$, rt, 45 min, 77%.

Neopentyl (4-(1-(((4-nitrophenoxy)carbonyl)oxy)but-3-yn-1-yl)phenyl) sulfate (S4)

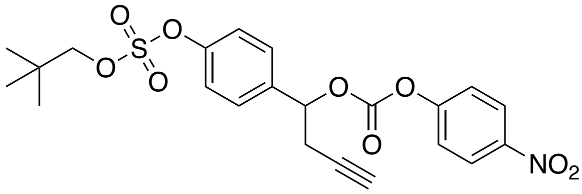

Triethylamine (408 µL, 2.93 mmol) was added dropwise to a solution of alcohol **9** (610 mg, 1.95 mmol) and 4-nitrophenylchloroformate (591 mg, 2.93 mmol) in THF (7 mL) at rt. The reaction mixture was stirred for 24 h before being diluted with EtOAc (30 mL) and washed with 1 M Na_2CO_3 (aq) (3 x 30 mL) and sat. NH_4Cl (aq) (30 mL). The organic fraction was dried ($MgSO_4$), concentrated *in vacuo* and purified by flash column chromatography (15-20% EtOAc in PE) to yield carbonate **S4** (573 mg, 1.20 mmol, 62%) as a colourless oil. **Rf** 0.45 (25% EtOAc in PE); $\mathbf{\nu_{max}}$ (neat/cm^{1}) 3297 (w), 2962 (w), 1766 (m), 1595 (w), 1526 (s), 1506 (w), 1347 (w); **^{1}H NMR** (400 MHz, $CDCl_3$): δ 8.27 (m, 2H), 7.52 (m, 2H), 7.36 (m, 4H), 5.84 (t, 1H, *J* = 6.6 Hz), 4.11 (s, 2H), 2.93 (m, 1H), 2.84 (m, 1H), 2.07 (t, 1H, *J* = 2.6 Hz), 1.01 (s, 9H); **^{13}C NMR** (101 MHz, $CDCl_3$): δ 155.4, 151.7, 150.7, 145.6, 136.7, 128.5, 125.5, 121.8, 121.5, 83.8, 78.2, 78.1, 72.0, 32.1, 26.6, 26.1; **HRMS** (ESI) *m/z* found $[M+Na]^+$ 500.0985 $C_{22}H_{23}NO_9SNa^+$, required 500.0991.

Neopentyl arylsulfate-4-alkyl-MMAE (S5)

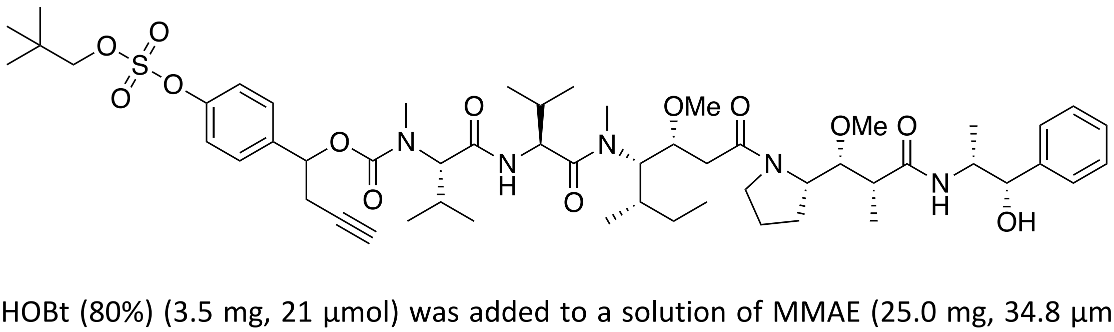

HOBt (80%) (3.5 mg, 21 µmol) was added to a solution of MMAE (25.0 mg, 34.8 µmol), neopentyl (4-(1-(((4-nitrophenoxy)carbonyl)oxy)but-3-yn-1-yl)phenyl) sulfate (**S4**) (30.3 mg, 63.4 µmol), pyridine (118 µL, 1.46 mmol) and DIPEA (7.3 µL, 41 µL) in DMF (0.2 mL) at rt and stirred for 48 h. The reaction mixture was diluted with EtOAc (20 mL) and washed with 1 M HCl (aq) (5 mL) and 1 M Na_2CO_3 (aq) (2 x 5 mL). The organic fraction was dried ($MgSO_4$), concentrated *in vacuo* and purified by flash column chromatography (0-3% MeOH in $CHCl_3$) to yield carbamate **S5** (30.1 mg, 28.4 µmol, 82%) as a yellow oil. **Rf** 0.39 (5% MeOH in $CHCl_3$); **HPLC** (5-95% $MeCN/H_2O$ over 20 min) retention time 14.471 min; **HRMS** (ESI) *m/z* found $[M+H]^+$ 1056.5976 $C_{55}H_{86}N_5O_{13}S^+$, required 1056.5937.

Ammonium arylsulfate-4-alkyl-MMAE (S6)

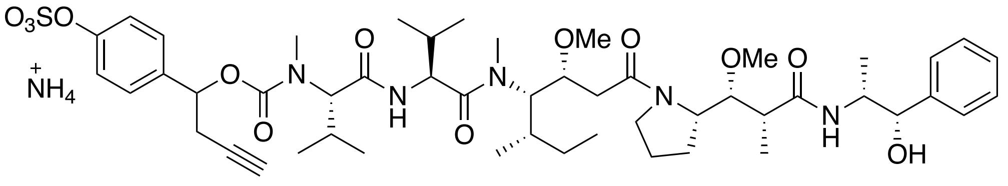

Carbamate **S5** (15.7 mg, 14.9 µmol) was dissolved in DMF (0.3 mL) and treated with 5 M NH_4OAc (aq) (0.6 mL) before stirring at 50 °C for 4 days. After cooling to rt, the reaction mixture was purified by reverse phase flash column chromatography (20-40% solvent B in solvent A. Solvent A: 0.1 M NH_4OH (aq). Solvent B: MeCN) and lyophilised to yield sulfate **S6** (11.9 mg, 11.9 µmol, 80%) as a white solid. **HRMS** (ESI) *m/z* found $[M-H^+]^-$ 984.4988 $C_{50}H_{74}N_5O_{13}S^-$, required 984.5009; **HPLC** (5-95% MeCN/H_2O over 20 min) retention time 11.457 min.

Ammonium DVP-PEG$_3$-arylsulfate-4-alkyl-MMAE (16a)

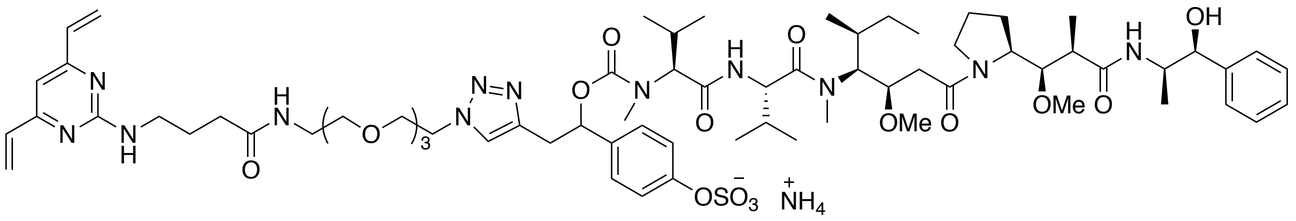

To a degassed solution of sulfate **S6** (3.00 mg, 3.00 µmol) and DVP **S16** (36.8 µL of 0.1 M solution in DMSO, 3.68 µmol) in ^{t}BuOH (0.1 mL) was added a degassed solution of $CuSO_4{\cdot}5H_2O$ (0.38 mg, 1.50 µmol), THPTA (1.30 mg, 3.00 µmol) and sodium ascorbate (1.30 mg, 6.10 µmol) in $H_2O/^tBuOH$ (0.3 mL, 1:1) and the reaction mixture was stirred at rt for 45 min. The reaction mixture was purified by reverse phase flash column chromatography (20-40% solvent B in solvent A. Solvent A: 0.1 M NH_4OH (aq). Solvent B: MeCN) and lyophilised to yield linker-drug **16a** (3.30 mg, 2.30 µmol, 77%) as a pale yellow solid. **HRMS** (ESI) *m/z* found $[M-H^+]^-$ 1417.7427 $C_{70}H_{105}N_{12}O_{17}S^-$, required 1417.7447; **HPLC** (5-95% MeCN/H_2O over 20 min) retention time 10.091 min.

Scheme S 5: Synthesis of linker-payload 16b.[a]

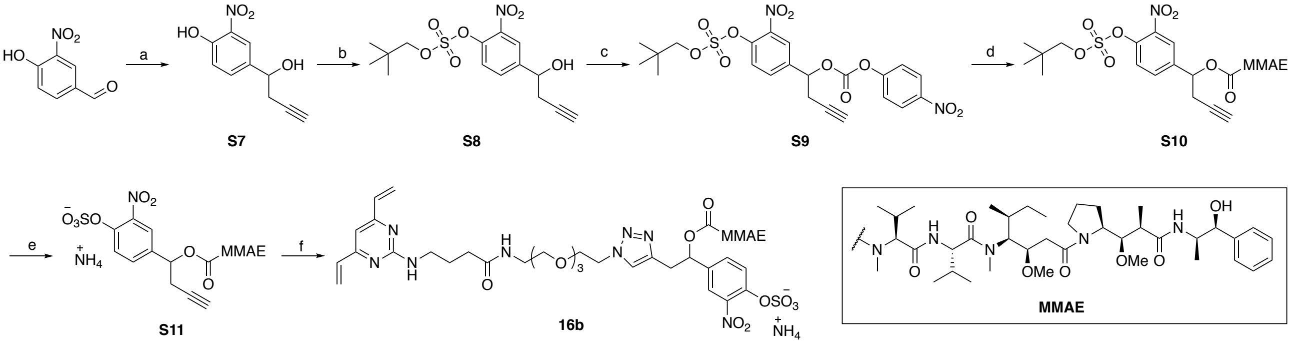

[a]Reagents and conditions: (a) propargyl bromide, magnesium, $ZnBr_2$, ether/THF, -78 °C for 15 h then 0 °C for 2 h, 75%; (b) Neopentyl sulfochloridate, 4-DMAP, Et_3N, THF, rt, 20 h, 64%; (c) 4-nitrophenyl chloroformate, pyridine, CH_2Cl_2, rt, 3 h, 89%; (d) MMAE, HOBt, pyridine, DIPEA, DMF, rt, 48 h, 68%; (e) 5 M NH_4OAc (aq), DMF, 50 °C, 2 days, 63%; (f) **S16**, $CuSO_4 \cdot 5H_2O$, THPTA, sodium ascorbate, $H_2O/^tBuOH$, rt, 2 h, 76%.

4-(1-hydroxybut-3-yn-1-yl)-2-nitrophenol (S7)

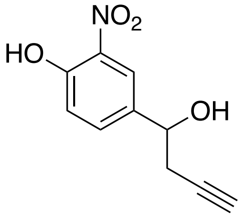

Propargyl bromide (2.00 mL, 26.4 mmol) in ether (16 mL) was added dropwise to a suspension of Mg (1.30 g, 53.5 mmol) and $ZnBr_2$ (240 mg, 1.07 mmol) in ether (20 mL). Upon exothermic reaction, the reaction mixture was cooled to 0 °C for the remainder of the addition. The supernatant was calculated to be 0.15 M by titration using 1,10 phenanthroline as indicator. The Grignard supernatant was added to a solution of 4-hydroxy-3-nitrobenzaldehyde (398 mg, 2.38 mmol) in THF (20 mL) at -78 °C and stirred for 15 h. The reaction mixture was then warmed to 0 °C for 2 h before being diluted with wet ether (20 mL) and 1 M HCl (aq) (20 mL). Upon warming to rt, the layers were separated, and the aqueous fraction was further extracted with EtOAc (2 x 30 mL). The combined organic fractions were dried ($MgSO_4$), concentrated *in vacuo* and purified by flash column chromatography (15-25% EtOAc in PE) to yield 4-(1-hydroxybut-3-yn-1-yl)-2-nitrophenol **S6** (368 mg, 1.78 mmol, 75%) as a yellow oil. **Rf** 0.49 (50% EtOAc in PE); **^{1}H NMR** (400 MHz, $CDCl_3$) δ 10.57 (s, 1H), 8.16 (s, 1H), 7.64 (d, 1H, J = 8.7 Hz), 7.17 (d, 1H J = 8.7 Hz), 4.89 (app q, 1H, J = 5.3 Hz), 2.65 (m, 2H), 2.46 (d, 1H, J = 3.7 Hz), 2.10 (t, 1H, J = 2.4 Hz); **^{13}C NMR** (101 MHz, $CDCl_3$) δ 154.8, 135.3, 135.0, 133.4, 122.4, 120.3, 79.7, 72.1, 70.9, 29.5.
Data in accordance with literature.[3]

4-(1-hydroxybut-3-yn-1-yl)-2-nitrophenyl neopentyl sulfate (S8)

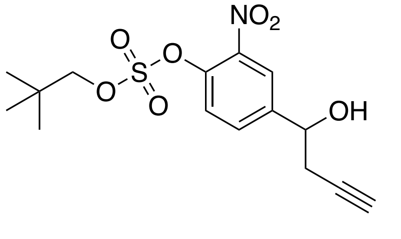

Neopentyl sulfurochloridate (0.558 mL, 3.51 mmol) was added dropwise to a solution of 4-(1-hydroxybut-3-yn-1-yl)-2-nitrophenol **S7** (364 mg, 1.76 mmol), 4-DMAP (215 mg, 1.76 mmol) and

triethylamine (0.493 mL, 3.51 mmol) in THF (18 mL) at rt. After 20 h the reaction mixture was concentrated *in vacuo*, diluted with EtOAc (40 mL) and washed with 1 M HCl (aq) (3 x 20 mL) and sat. $NaHCO_3$ (aq) (20 mL). The organic fraction was dried ($MgSO_4$), concentrated *in vacuo* and purified by flash column chromatography (10-25% EtOAc in PE + 0.5% AcOH) to yield 4-(1-hydroxybut-3-yn-1-yl)-2-nitrophenyl neopentyl sulfate **S8** (403 mg, 1.13 mmol, 64%) as a yellow oil. **Rf** 0.20 (25% EtOAc in PE + 0.5% AcOH); $\mathbf{\nu_{max}}$ (neat/cm^{1}) 3152 (br), 2901 (w), 1625 (w), 1510 (w), 1354 (m), 1300 (m); **^{1}H NMR** (400 MHz, $CDCl_3$) δ 8.10 (d, 1H, *J* = 2.1 Hz), 7.72 (dd, 1H, *J* = 8.6, 2.1 Hz), 7.62 (d, 1H, *J* = 8.6 Hz), 4.97 (t, 1H, *J* = 6.2 Hz), 4.22 (s, 2H), 2.67 (m, 2H), 2.13 (t, 1H, *J* = 2.6 Hz), 1.03 (s, 9H); **^{13}C NMR** (101 MHz, $CDCl_3$) δ 142.9, 142.0, 141.5, 131.9, 124.0, 123.7, 85.2, 79.1, 72.5, 70.7, 32.1, 29.6, 26.0; **HRMS** (ESI) *m/z* found $[M-H^+]^-$ 356.0811 $C_{15}H_{18}NO_7S^-$, required 356.0809.

Neopentyl (2-nitro-4-(1-(((4-nitrophenoxy)carbonyl)oxy)but-3-yn-1-yl)phenyl) sulfate (S9)

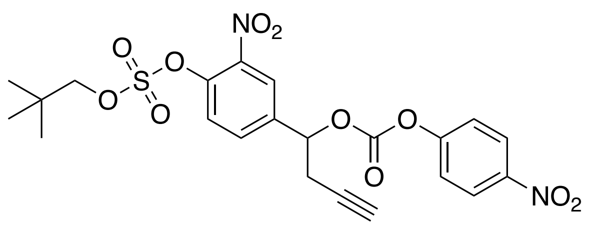

Pyridine (15.5 µL, 192 µmol) was added to a solution of alcohol **S8** (30.0 mg, 96.0 µmol) and 4-nitrophenylchloroformate (23.2 mg, 115 µmol) in CH_2Cl_2 (1 mL) at rt. The reaction mixture was stirred for 3 h before being diluted with EtOAc (20 mL) and washed with 1 M Na_2CO_3 (aq) (20 mL) and 1 M HCl (aq) (20 mL). The organic fraction was dried ($MgSO_4$), concentrated *in vacuo* and purified by flash column chromatography (15-20% EtOAc in PE) to yield carbonate **S9** (40.8 mg, 85.4 µmol, 89%) as a pale yellow oil. **Rf** 0.65 (50% EtOAc in PE); $\mathbf{\nu_{max}}$ (neat/cm^{1}) 2906 (w), 1771 (s), 1555 (w), 1501 (m), 1488 (m); **^{1}H NMR** (400 MHz, $CDCl_3$) δ 8.29 (d, 2H, *J* = 9.2 Hz), 8.15 (d, 1H, *J* = 2.2 Hz), 7.78 (dd, 1H, *J* = 8.6, 2.2 Hz), 7.70 (d, 1H, *J* = 8.6 Hz), 7.39 (d, 2H, *J* = 9.2 Hz), 5.88 (t, 1H, *J* = 6.5 Hz), 4.25 (s, 2H), 2.93 (m, 2H), 2.12 (t, 1H, *J* = 2.6 Hz), 1.04 (s, 9H); **^{13}C NMR** (101 MHz, $CDCl_3$) δ 155.2, 151.6, 145.8, 142.5, 137.7, 132.8, 132.7, 125.5, 124.5, 124.4, 121.8, 85.5, 77.4, 76.8, 73.0, 32.1, 26.4, 26.0; **HRMS** (ESI) *m/z* found $[M+Na]^+$ 545.0865 $C_{22}H_{22}N_2O_{11}SNa^+$, required 545.0842.

Neopentyl-nitroarylsulfate-MMAE (S10)

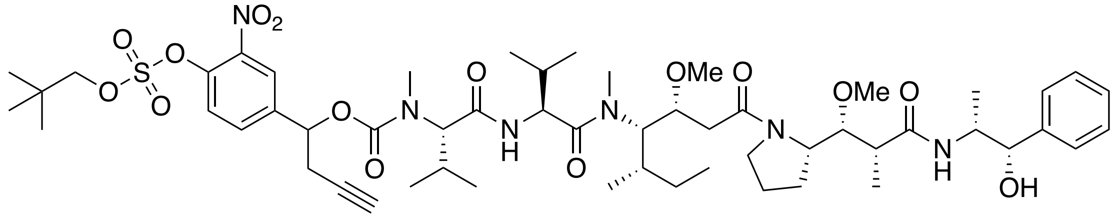

Carbonate **S9** (10.0 mg, 19.1 µmol), pyridine (54.0 µL, 670 µmol) and MMAE (20.6 mg, 28.7 µmol) were dissolved in DMF (0.2 mL) before the addition of DIPEA (3.30 µL, 19.0 µL) and HOBt (80%) (3.40 mg, 20.0 µmol) at rt. After 48 h, the reaction mixture was diluted with EtOAc (20 mL) and washed with 1 M HCl (aq) (5 mL) and 1 M Na_2CO_3 (aq) (3 x 5 mL). The organic fraction was dried ($MgSO_4$), concentrated *in vacuo* and purified by flash column chromatography (0-5% MeOH in CH_2Cl_2) to yield carbamate **S10** (14.4 mg, 13.1 µmol, 68%) as a yellow solid. **Rf** 0.26 (3% MeOH in CH_2Cl_2); **HRMS** (ESI) *m/z* found $[M+H]^+$ 1101.5776 $C_{55}H_{85}N_6O_{15}S^+$, required 1101.5794; **HPLC** (5-95% MeCN/H_2O over 20 min) retention time 14.695 min.

Ammonium nitroarylsulfate-MMAE (S11)

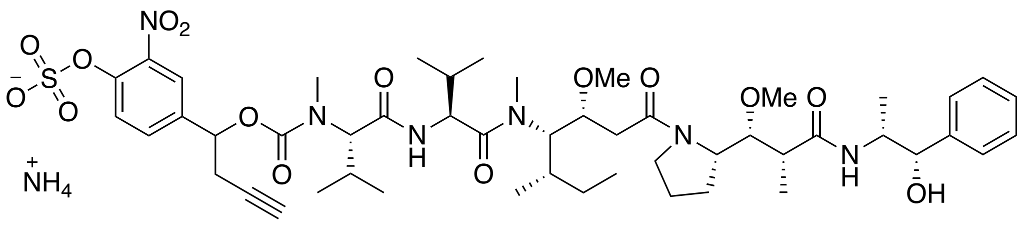

Carbamate **S10** (5.90 mg, 5.40 µmol) was dissolved in DMF (0.2 mL) and treated with 5 M NH_4OAc (aq) (0.2 mL) before stirring at 50 °C for 2 days. The cooled reaction mixture was purified by reverse phase flash column chromatography (30-70% solvent B in solvent A. Solvent A: 0.1 M NH_4OH (aq). Solvent B: MeCN) and lyophilised to yield sulfate **S11** (3.70 mg, 3.40 µmol, 63%) as a white solid. **HRMS** (ESI) *m/z* found $[M-H^+]^-$ 1029.4824 $C_{50}H_{73}N_6O_{15}S^-$, required 1029.4860; **HPLC** (5-95% $MeCN/H_2O$ over 20 min) retention time 12.925 min.

Ammonium DVP-PEG$_3$-nitroarylsulfate-MMAE (16b)

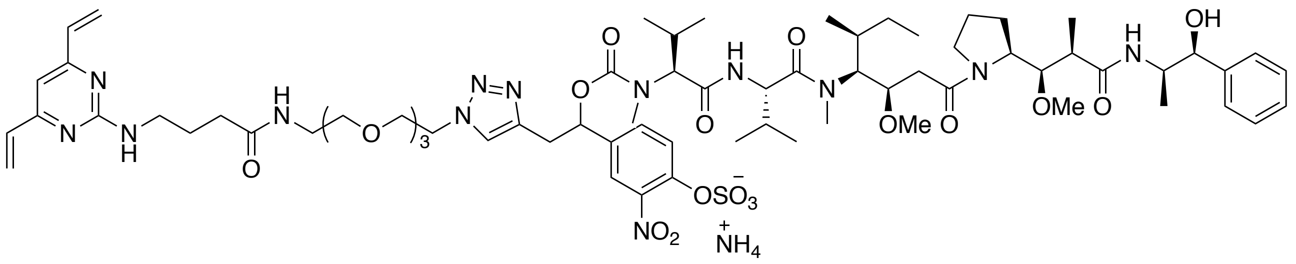

To a degassed solution of sulfate **S11** (3.00 mg, 2.90 µmol) and DVP **S16** (38.2 µL of 0.1 M solution in DMSO, 3.82 µmol) in ^{t}BuOH (0.1 mL) was added a degassed solution of $CuSO_4 \cdot 5H_2O$ (0.600 mg, 2.30 µmol), THPTA (1.70 mg, 3.80 µmol) and sodium ascorbate (1.90 mg, 9.50 µmol) in $H_2O/^tBuOH$ (0.3 mL, 1:1) and the reaction mixture was stirred at rt for 2 h. The reaction mixture was purified by reverse phase flash column chromatography (30-50% solvent B in solvent A. Solvent A: 0.1 M NH_4OH (aq). Solvent B: MeCN) and lyophilised to yield linker-drug **16b** (3.30 mg, 2.20 µmol, 76%) as a yellow solid. **HRMS** (ESI) *m/z* found $[M-H^+]$ 1462.7298 $C_{70}H_{104}N_{13}O_{19}S^-$, required 1462.7263; **HPLC** (5-95% $MeCN/H_2O$ over 20 min) retention time 10.932 min.

Scheme S 6 : Synthesis of linker-payload 17.[a]

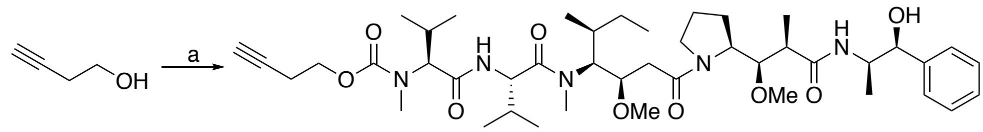

S12

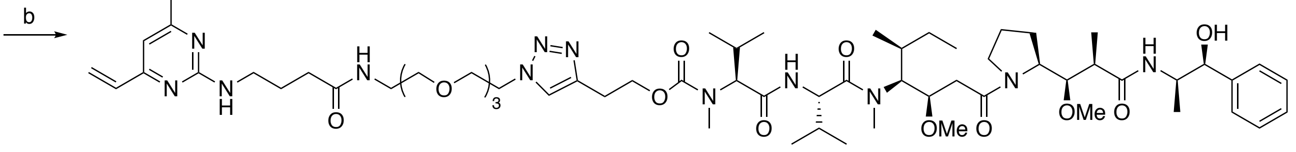

17

[a]Reagents and conditions: (a) 4-nitrophenylchloroformate, pyridine, CH_2Cl_2, rt, 2 h then MMAE, HOBt, DIPEA, DMF, rt, 24 h, 40%; (b) **S16**, $CuSO_4{\cdot}5H_2O$, THPTA, sodium ascorbate, $H_2O/^tBuOH$, rt, 18 h, 22%.

Alkyne-MMAE (S12)

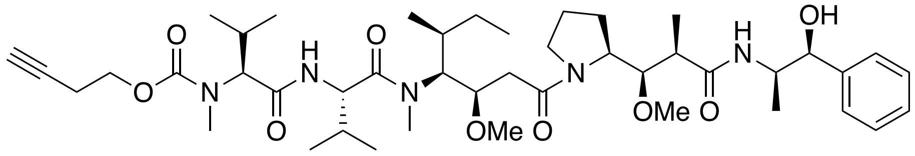

Pyridine (7.50 µL, 93.0 µmol) was added to a solution of 3-butyn-1-ol (3.70 µL, 48.0 µmol) and 4-nitrophenylchloroformate (3.70 mg, 19.0 µmol) in CH_2Cl_2 (0.2 mL) and stirred at rt for 2 h. MMAE (20.0 mg, 27.9 µmol), HOBt (70%) (1.8 mg, 9.5 µmol), DIPEA (2.4 µL, 19 µmol) and DMF (0.2 mL) were added to the reaction mixture and stirred at rt for 24 h before being diluted with EtOAc (20 mL) and washed with 1 M HCl (aq) (15 mL) and 1 M Na_2CO_3 (aq) (2 x 10 mL). The organic fraction was dried ($MgSO_4$), concentrated *in vacuo* and purified by flash column chromatography (0-3% MeOH in CH_2Cl_2) to yield alkyne **S12** (6.00 mg, 7.40 µmol, 40%) as a pale yellow oil. **Rf** 0.43 (5% MeOH in CH_2Cl_2); **HRMS** (ESI) *m/z* found $[M+H]^+$ 814.5308 $C_{44}H_{72}N_5O_9^+$, required 814.5330; **HPLC** (5-95% MeCN/H_2O over 20 min) retention time 13.320 min.

DVP-PEG$_3$-MMAE (17)

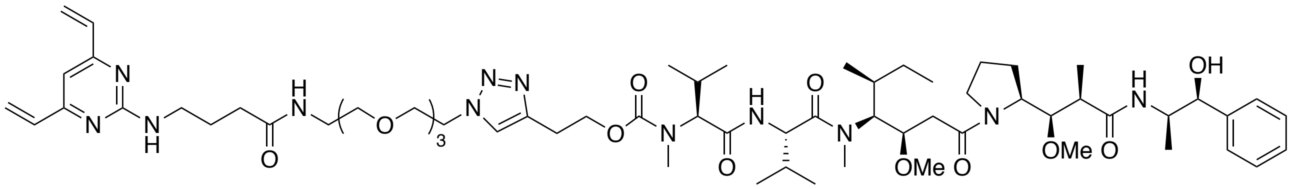

To a degassed solution of alkyne **S12** (5.00 mg, 6.10 µmol) and DVP **S16** (73.7 µL of 0.1 M solution in DMSO, 7.37 µmol) in ^tBuOH (0.1 mL) was added a degassed solution of $CuSO_4{\cdot}5H_2O$ (0.800 mg, 3.10 µmol), THPTA (2.70 mg, 6.10 µmol) and sodium ascorbate (2.40 mg, 12.0 µmol) in $H_2O/^tBuOH$ (0.3 mL, 1:1) and the reaction mixture was stirred at rt for 18 h. The reaction mixture was purified by reverse phase flash column chromatography (50-70% solvent B in solvent A. Solvent A: 0.1 M NH_4OH (aq). Solvent B: MeCN) and lyophilised to yield linker-drug **17** (1.70 mg, 1.40 µmol, 22%) as a pale yellow solid. **HRMS** (ESI) *m/z* found $[M+H]^+$ 1247.7722 $C_{64}H_{103}N_{12}O_{13}^+$, required 1247.7762; **HPLC** (5-95% MeCN/H_2O over 20 min) retention time 9.405 min.

Scheme S 7: Synthesis of DVP-azide (S15).[a]

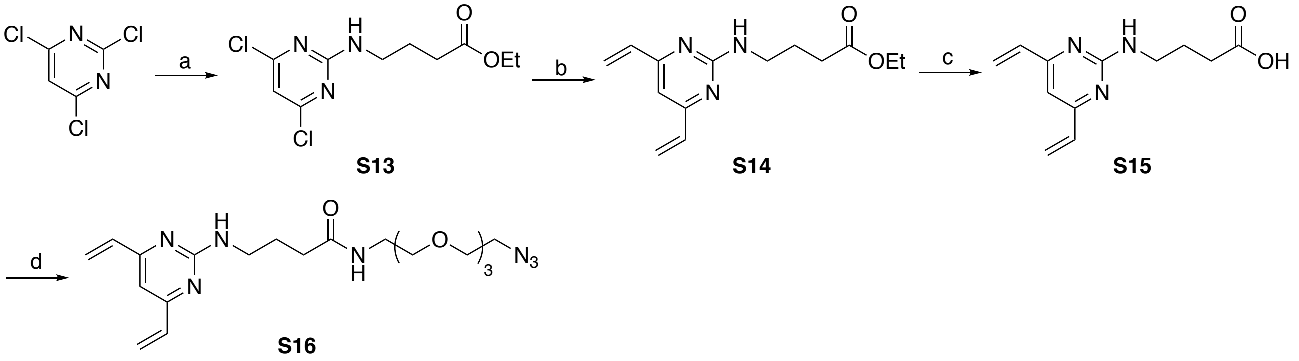

[a]Reagents and conditions: (a) ethyl 4-aminobutyrate hydrochloride, Et_3N, acetone, 0 °C for 5 min then rt for 90 min, 28%; (b) potassium vinyltrifluoroborate, Pd(dppf)Cl_2·CH_2Cl_2, potassium carbonate, THF/H_2O, 90 °C, 4 h, 100%; (c) Lithium hydroxide monohydrate, THF/MeOH/H_2O, rt, 2 days, 80%; (d) HATU, 11-azido-3,6,9-trioxaundecan-1-amine, HOBt, DIPEA, rt, 2 h, 70%.

Ethyl 4-((4,6-dichloropyrimidin-2-yl)amino)butanoate (S13)

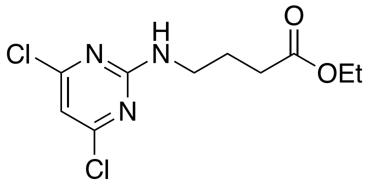

Triethylamine (4.57 mL, 32.8 mmol) was added dropwise to a solution of 2,4,6-trichloropyrimidine (2.00 g, 10.9 mmol) and ethyl 4-aminobutyrate hydrochloride (2.19 g, 13.1 mmol) in acetone (12 mL) at 0 °C. After 5 min, the reaction mixture was warmed to rt and stirred for 90 min. The reaction mixture was concentrated *in vacuo* then diluted with EtOAc (30 mL) and washed with H_2O (2 x 30 mL) and brine (30 mL). The organic fraction was dried ($MgSO_4$), concentrated *in vacuo* and purified by flash column chromatography (10% EtOAc in PE) to yield ester **S13** (840 mg, 3.02 mmol, 28%) as a clear oil. **Rf** 0.22 (10% EtOAc in PE); $\mathbf{v_{max}}$ (neat/cm^{1}) 3371 (m), 2981 (w), 2937 (w), 1735 (s), 1583 (s), 1563 (s), 1518 (s), 1450 (m), 1375 (m); ^{1}**H NMR** (400 MHz, $CDCl_3$) δ 6.59 (s, 1H), 5.56 (br s, 1H), 4.14 (q, 2H, *J* = 7.1 Hz), 3.48 (q, 2H, *J* = 6.6 Hz), 2.39 (t, 2H, *J* = 7.2 Hz), 1.93 (t, 2H, *J* = 7.0 Hz), 1.25 (t, 3H, *J* = 7.1 Hz); ^{13}**C NMR** (101 MHz, $CDCl_3$) δ 173.3, 161.8, 109.2, 60.8, 41.1, 31.7, 24.7, 14.4; **HRMS** (ESI) *m/z* found $[M+H]^+$ 278.0450, $C_{10}H_{14}N_3O_2Cl_2^+$ required 278.0458.

Ethyl 4-((4,6-divinylpyrimidin-2-yl)amino)butanoate (S14)

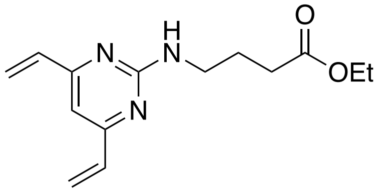

Ethyl 4-((4,6-dichloropyrimidin-2-yl)amino)butanoate **S13** (401 mg, 1.44 mmol), potassium vinyltrifluoroborate (580 mg, 4.33 mmol), Pd(dppf)Cl_2·CH_2Cl_2 (118 mg, 0.144 mmol) and potassium carbonate (1.20 g, 8.66 mmol) were suspended in THF/H_2O (10:1, 4.8 mL) and heated to 90 °C for 4 h in a sealed tube. The reaction mixture was filtered through Celite® and concentrated *in vacuo*. The crude residue was purified by flash column chromatography (25% EtOAc in PE) to yield DVP **S14** (377 mg, 1.44 mmol, 100%) as a pale yellow oil. **Rf** 0.35 (25% EtOAc in PE); $\mathbf{v_{max}}$ (neat/cm^{1}) 3394 (w), 2980 (w), 1729 (s), 1635 (w), 1539 (s), 1324 (m); ^{1}**H NMR** (400 MHz, $CDCl_3$) δ 6.57 (m, 2H),

6.52 (s, 1H), 6.35 (d, 2H, *J* = 17.2 Hz), 5.55 (dd, 2H *J* = 10.6, 1.5 Hz), 5.10 (t, 1H, *J* = 5.5 Hz), 4.13 (q, 2H, *J* = 7.1 Hz), 3.53 (q, 2H, *J* = 6.6 Hz), 2.41 (t, 2H, *J* = 7.4 Hz), 1.96 (quint, 2H *J* = 7.1 Hz), 1.24 (t, 3H, *J* = 7.1 Hz); **^{13}C NMR** (101 MHz, $CDCl_3$) δ 173.6, 163.9, 162.8, 136.1, 121.5, 105.9, 60.5, 40.9, 31.9, 25.3, 14.4; **HRMS** (ESI) *m/z* found $[M+H]^+$ 262.1556, $C_{14}H_{20}N_3O_2^+$ required 262.1556.

4-((4,6-divinylpyrimidin-2-yl)amino)butanoic acid (S15)

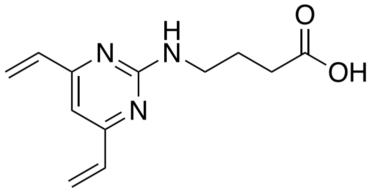

Lithium hydroxide monohydrate (119 mg, 2.85 mmol) was added to a solution of ethyl 4-((4,6-divinylpyrimidin-2-yl)amino)butanoate **S14** (620 mg, 2.37 mmol) in THF (5 mL), MeOH (2 mL) and H_2O (5 mL) and stirred at rt for 2 days. The reaction mixture was concentrated *in vacuo* before being diluted with sat. NH_4Cl (aq) (40 mL), and extracted with 10% ^{i}PrOH/EtOAc (4 x 120 mL), with 1 M HCl (aq) adjusting to pH 4 between extractions. The combined organic fractions were dried ($MgSO_4$) and concentrated *in vacuo* to yield carboxylic acid **S15** (445 mg, 1.91 mmol, 80%) as a yellow solid. **Rf** 0.39 (5% MeOH in CH_2Cl_2 + 0.5% AcOH); **ν_{max}** (neat/cm^1) 3311 (br), 2919 (w), 2150 (w), 1703 (m), 1559 (s), 1413 (m); **^{1}H NMR** (400 MHz, CD_3OD) δ 6.69 (s, 1H), 6.60 (dd, 2H, *J* = 17.4, 10.7 Hz), 6.36 (d, 2H, *J* = 17.0 Hz), 5.57 (dd, 2H, *J* = 10.7, 1.5 Hz), 3.47 (t, 2H, *J* = 6.9 Hz), 2.37 (t, 2H, *J* = 7.4 Hz), 1.91 (m, 2H); **^{13}C NMR** (101 MHz, CD_3OD) δ 177.4, 165.3, 164.0, 137.0, 122.2, 105.7, 41.5, 32.3, 26.1; **HRMS** (ESI) *m/z* found $[M+H]^+$ 234.1254, $C_{12}H_{16}N_3O_2^+$ required 234.1243.

***N*-(2-(2-(2-(2-azidoethoxy)ethoxy)ethoxy)ethyl)-4-((4,6-divinylpyrimidin-2-yl)amino)butanamide (S16)**

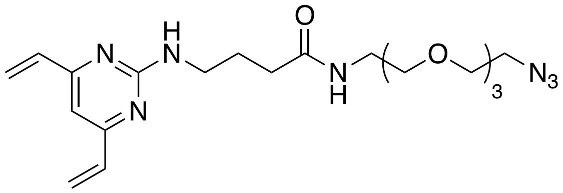

DIPEA (149 µL, 0.429 mmol) was added to a solution of 4-((4,6-divinylpyrimidin-2-yl)amino)butanoic acid **S15** (100 mg, 0.429 mmol), 11-azido-3,6,9-trioxaundecan-1-amine (90%) (113 µL, 0.515 mmol), HATU (195 mg, 0.515 mmol), HOBt (80%) (72.2 mg, 0.858 mmol) in DMF (2 mL) and stirred at rt. After 2 h, sat. $NaHCO_3$ (aq) (20 mL) was added to the reaction mixture and extracted with EtOAc (5 x 20 mL). The combined organic fractions were dried ($MgSO_4$), concentrated *in vacuo* and purified by flash column chromatography (10% acetone in EtOAc) to yield azide **S16** (131 mg, 0.302 mmol, 70%) as a yellow oil; **Rf** 0.17 (10% acetone in EtOAc); **ν_{max}** (neat/cm^1) 3341 (w), 2868 (w), 2108 (m), 1650 (w), 1544 (s) **^{1}H NMR** (400 MHz, $CDCl_3$) δ 6.57 (m, 2H), 6.53 (s, 1H), 6.34 (d, 2H, *J* = 17.3 Hz), 6.16 (br s, 1H), 5.56 (dd, 2H, *J* = 10.6, 1.4 Hz), 5.21 (t, 1H, *J* = 5.6 Hz), 3.65 (m, 8H), 3.60 (m, 2H), 3.53 (m, 4H), 3.45 (m, 2H), 3.37 (t, 2H, *J* = 5.0 Hz), 2.29 (t, 2H, *J* = 7.3 Hz), 1.97 (quint, 2H, *J* = 9.3 Hz); **NMR** (101 MHz, $CDCl_3$) δ 172.8, 163.9, 162.9, 136.1, 121.5, 105.7, 70.9, 70.8, 70.7, 70.4, 70.2, 70.0, 50.8, 40.9, 39.4, 34.0, 25.9; **HRMS** (ESI) *m/z* found $[M+H]^+$ 434.2513, $C_{20}H_{32}N_7O_4^+$ required 434.2516.

Scheme S 8: Synthesis of Val-Ala-PABC-AMC 13.[a]

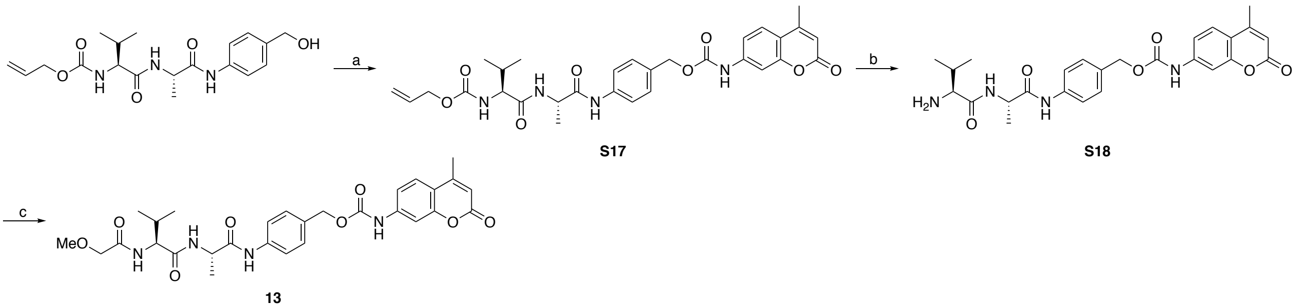

[a]Reagents and conditions: (a) AMC, triphosgene, toluene, reflux, 1 h, then dibutyltin dilaurate, THF, DMF, rt, 3 h, then 45 °C for 2 h, 56%; (b) $Pd(PPh_3)_4$, AcOH, tributyltin hydride, 0 °C, 1 h, 73%; (c) methoxyacetic acid, HATU, DIPEA, DMF, rt, 1 h, 85%.

Alloc-Val-Ala-PABC-AMC (S17)

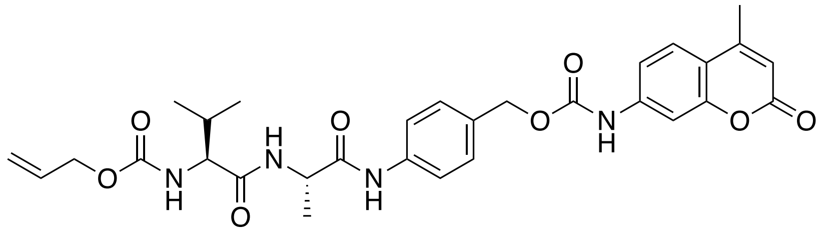

7-Amino-4-methylcoumarin (23.2 mg, 133 µmol) and triphosgene (19.7 mg, 66.3 µmol) were suspended in toluene (1.5 mL) and refluxed for 1 h. The reaction mixture was cooled and evaporated under a stream of nitrogen before a solution of Alloc-Val-Ala-PABOH (50.0 mg, 133 µmol) in THF (1.5 mL) was added. Dibutyltin dilaurate (7.90 µL, 13.3 µmol) was added to the resulting suspension and stirred at rt for 3 h before DMF (4 mL) was added. After stirring for 15 h, additional DMF (2 mL) was added and the resulting solution was stirred at 45 °C for 2 h. The cooled reaction mixture was then quenched and precipitated with H_2O (20 mL) and filtered, washing with additional H_2O (10 mL), MeOH (3 x 10 mL) and hot MeCN (20 mL). The resulting solid was dried *in vacuo* to yield coumarin **S17** (42.8 mg, 74.0 µmol, 56%) as a light beige solid. $\mathbf{v_{max}}$ (neat/cm^{1}) 3286 (w), 2957 (w), 1729 (m), 1695 (m), 1618 (m), 1578 (s), 1532 (s); **^{1}H NMR** (400 MHz, DMSO-d_6) δ 10.24 (s, 1H), 10.02 (s, 1H), 8.16 (d, 1H, *J* = 6.8 Hz), 7.68 (d, 1H, *J* = 8.7 Hz), 7.61 (d, 2H, *J* = 8.3 Hz), 7.55 (d, 1H, *J* = 1.4 Hz), 7.40 (m, 3H, *J* = 5.5 Hz), 7.24 (d, 1H, *J* = 8.7 Hz), 6.23 (s, 1H), 5.91 (m, 1H), 5.29 (d, 1H, *J* = 17.0 Hz), 5.15 (m, 3H), 4.44 (m, 3H), 3.89 (t, 1H, *J* = 7.7 Hz), 2.38 (s, 3H), 1.97 (m, 1H), 1.31 (d, 3H, *J* = 7.0 Hz), 0.88 (d, 3H, *J* = 6.7 Hz), 0.84 (d, 3H, *J* = 6.6 Hz); **^{13}C NMR** (101 MHz, DMSO-d_6) δ 171.2, 171.0, 160.1, 160.0, 156.0, 153.9, 153.8, 153.2, 142.7, 139.0, 133.6, 130.8, 129.2, 126.0, 119.0, 116.9, 114.4, 114.2, 111.9, 104.4, 66.0, 64.4, 59.9, 49.0, 30.3, 19.2, 18.1, 18.04, 17.98; **HRMS** (ESI) *m/z* found $[M+H]^+$ 579.2459 $C_{30}H_{35}N_4O_8^+$, required 579.2450.

H_2N-Val-Ala-PABC-AMC (S18)

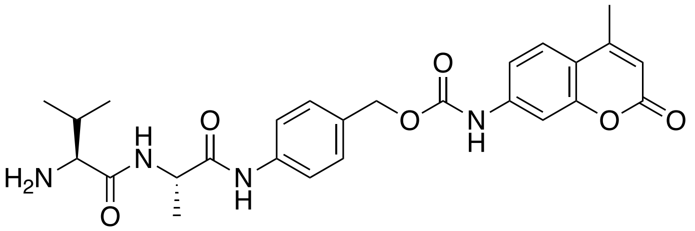

To a solution of coumarin **S17** (29.0 mg, 50.1 µmol) in THF (0.5 mL) and DMF (0.5 mL) at 0 °C was added $Pd(PPh_3)_4$ (5.80 mg, 5.00 µmol), AcOH (5.70 µL, 100 µmol) and tributyltin hydride (26.9 µL,

100 μmol). After 1 h, the reaction mixture was warmed to rt and purified by flash column chromatography (10% MeOH in CH_2Cl_2 + 0.5% triethylamine) to yield amine **S18** (18.1 mg, 36.6 μmol, 73%) as a beige solid. **Rf** 0.11 (10% MeOH in CH_2Cl_2 + 0.5% triethylamine); **^{1}H NMR** (400 MHz, DMSO-d_6) δ 10.4 (s, 1H), 10.3 (s, 1H), 8.80 (d, 1H, J = 7.0 Hz), 8.24 (d, 3H, J = 4.2 Hz), 7.67 (m, 3H), 7.56 (d, 1H), 7.40 (m, 3H), 6.23 (d, 1H, J = 1.2 Hz), 5.13 (s, 2H), 4.51 (quint, 1H, J = 7.0 Hz), 3.65 (t, 1H, J = 5.5 Hz), 2.38 (d, 3H, J = 1.1 Hz), 2.11 (m, 1H), 1.36 (d, 3H, J = 7.1 Hz), 0.96 (d, 3H, J = 1.8 Hz), 0.95 (d, 3H, J = 1.8 Hz); **^{13}C NMR** (101 MHz, DMSO-d_6) δ 170.8, 167.5, 160.0, 153.8, 153.2, 142.8, 139.0, 130.9, 129.1, 126.0, 119.1, 114.4, 114.3, 111.9, 104.4, 66.0, 57.1, 49.3, 48.6, 29.8, 18.3, 18.1, 18.0, 17.9. **HRMS** (ESI) m/z found $[M+H]^+$ 495.2249 $C_{26}H_{31}N_4O_6^+$, required 495.2238.

4-((*S*)-2-((*S*)-2-(2-methoxyacetamido)-3-methylbutanamido)propanamido)benzyl (4-methyl-2-oxo-2*H*-chromen-7-yl)carbamate (13)

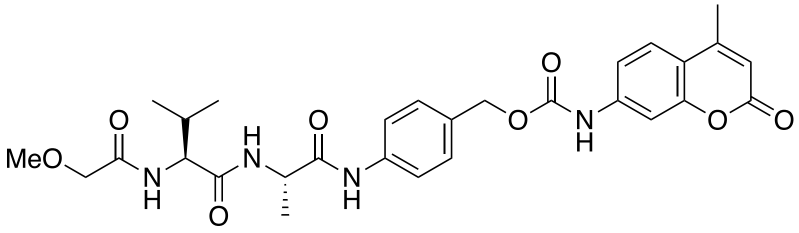

DIPEA (7.90 μL, 46.0 μmol) was added to a solution of amine **S18** (9.40 mg, 19.0 μmol), 2-methoxyacetic acid (5.20 μL, 68.0 μmol), HATU (13.0 mg, 34.1 μmol) and HOBt (80%) (2.40 mg, 23.0 μmol) in DMF (0.2 mL) at rt. After 1 h, the reaction mixture was diluted with EtOAc (20 mL) and washed with 1 M HCl (aq) (10 mL) and sat. $NaHCO_3$ (aq) (10 mL). The organic fraction was dried ($MgSO_4$), concentrated *in vacuo* and purified by flash column chromatography (2-5% MeOH in CH_2Cl_2) to yield dipeptide **13** (9.05 mg, 16.0 μmol, 85%) as a white solid. **Rf** 0.34 (5% MeOH in CH_2Cl_2); **ν_{max}** (neat/cm^1) 3282 (w), 2938 (w), 1699 (s), 1640 (s), 1532 (s); **^{1}H NMR** (400 MHz, DMSO-d_6) δ 10.25 (s, 1H), 10.05 (s, 1H), 8.40 (d, 1H, J = 6.9 Hz), 7.69 (d, 1H, J = 8.7 Hz), 7.62 (d, 2H, J = 8.5 Hz), 7.55 (d, 1H, J = 2.0 Hz), 7.46 (d, 1H, J = 9.0 Hz), 7.40 (m, 3H), 6.23 (d, 1H, J = 1.1 Hz), 5.13 (s, 2H), 4.41 (t, 1H, J = 7.0 Hz), 4.28 (dd, 1H, J = 9.0, 6.6 Hz), 3.86 (d, 2H, J = 1.9 Hz), 2.38 (d, 3H, J = 1.0 Hz), 2.00 (m, 1H), 1.31 (d, 3H, J = 7.1 Hz), 0.88 (d, 3H, J = 6.8 Hz), 0.82 (d, 3H, J = 6.8 Hz); **^{13}C NMR** (101 MHz, DMSO-d_6) δ 171.1, 170.5, 168.7, 160.0, 153.8, 153.2, 142.7, 139.0, 130.8, 129.2, 126.0, 120.1, 119.0, 114.4, 114.2, 111.9, 104.4, 71.2, 69.8, 66.0, 58.5, 56.6, 49.1, 31.0, 19.1, 18.00, 17.98, 17.9; **HRMS** (ESI) m/z found $[M+H]^+$ 567.2444 $C_{29}H_{35}N_4O_8^+$, required 567.2449.

Scheme S 9: Synthesis of Val-Cit-PABC-AMC 14.[a]

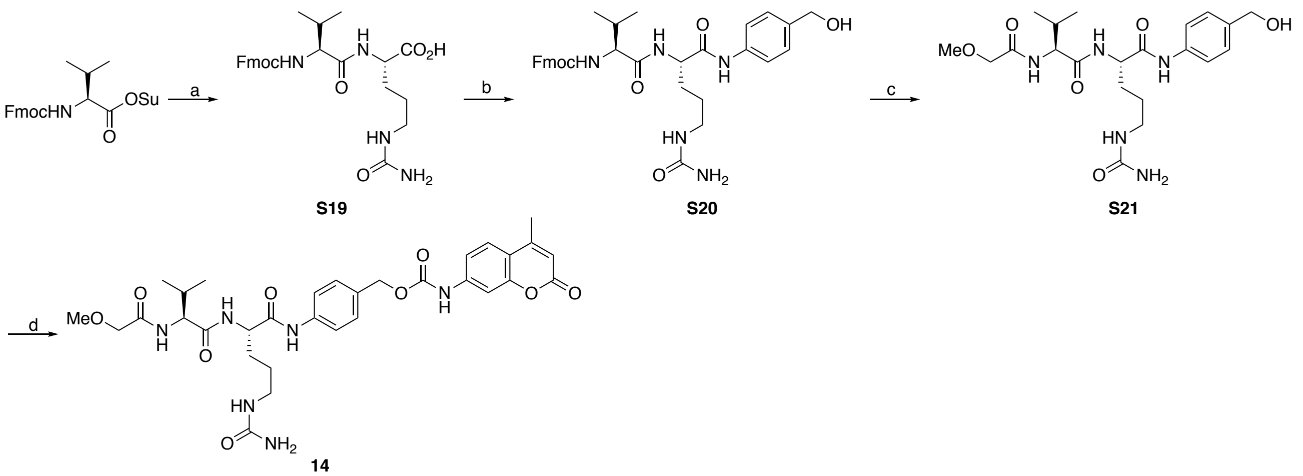

[a]Reagents and conditions: (a) citrulline, $NaHCO_3$, DME, THF, H_2O, 0 °C for 5 min then rt for 28 h, 87%; (b) 4-aminobenzyl alcohol, EEDQ, $MeOH/CH_2Cl_2$, 40 °C, 18 h, 63%; (c) Et_3N, DMF, rt, 18 h then methoxyacetyl chloride, 0 °C for 30 min then rt for 2 h, 66%; (d) AMC, triphosgene, toluene, reflux, 1 h, then dibutyltin dilaurate, DMF, rt, 48 h, 20%.

Fmoc-Val-Cit-OH (S19)

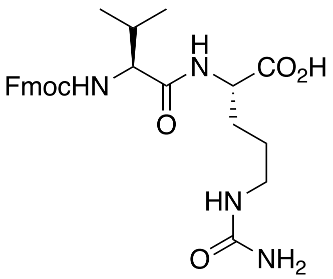

A solution of Fmoc-Val-OSu (2.00 g, 4.58 mmol) in DME (15 mL) was added to a suspension of citrulline (842 mg, 4.81 mmol) and $NaHCO_3$ (423 mg, 5.04 mmol) in H_2O (30 mL) and THF (8 mL) at 0 °C. After 5 min, the reaction mixture was warmed to rt and stirred for 28 h. The reaction mixture was then adjusted to pH 10 with sat. K_2CO_3 (aq) before being diluted with H_2O (50 mL) and extracted with EtOAc (4 x 100 mL). The organic fractions were combined and added to 30 wt% citric acid (aq) (30 mL) before the mixture was filtered. The resulting filter cake was dried *in vacuo* to yield Fmoc-Val-Cit-OH **S18** (1.96 g, 3.96 mmol, 87%) as a white solid. **^{1}H NMR** (400 MHz, DMSO-d_6) δ 8.16 (d, 1H, J = 7.3 Hz), 7.89 (d, 2H, J = 7.5 Hz), 7.75 (t, 2H, J = 7.1 Hz), 7.41 (m, 3H), 7.32 (m, 2H), 5.94 (t, 1H, J = 5.6 Hz), 5.37 (s, 2H), 4.21 (m, 4H), 3.92 (m, 1H), 3.60 (m, 2H), 2.95 (q, 2H, J = 6.3 Hz), 1.97 (m, 1H), 1.72 (m, 1H), 1.57 (m, 1H), 1.41 (m, 2H), 0.89 (d, 3H, J = 7.0 Hz), 0.86 (d, 3H, J = 6.7 Hz); **^{13}C NMR** (101 MHz, DMSO-d_6) δ 173.6, 171.5, 158.8, 156.1, 144.0, 140.7, 127.7, 127.1, 125.4, 120.1, 71.1, 65.7, 60.1, 52.4, 46.7, 30.5, 28.8, 26.6, 19.2, 18.2.
Data in accordance with literature.[4]

Fmoc-Val-Cit-PABOH (S20)

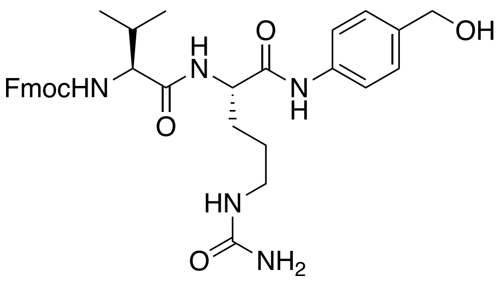

Fmoc-Val-Cit-OH **S18** (1.00 g, 2.02 mmol), 4-aminobenzyl alcohol (496 mg, 4.02 mmol) and *N*-Ethoxycarbonyl-2-ethoxy-1,2-dihydroquinoline (996 mg, 4.02 mmol) were dissolved in MeOH (3.8 mL) and CH_2Cl_2 (9.6 mL) and stirred at 40 °C. After 18 h, the cooled reaction mixture was filtered, washing with ether (2 x 20 mL) to yield Fmoc-Val-Cit-PABOH **S19** (770 mg, 1.28 mmol, 63%) as a white solid. **^{1}H NMR** (400 MHz, DMSO-d_6) δ 9.98 (s, 1H), 8.11 (d, 1H, *J* = 7.6 Hz), 7.89 (d, 2H, *J* = 7.5 Hz), 7.74 (m, 2H), 7.54 (d, 2H, *J* = 8.4 Hz), 7.42 (m, 3H), 7.32 (m, 2H), 7.23 (d, 2H, *J* = 8.4 Hz), 5.98 (t, 1H, *J* = 5.7 Hz), 5.41 (s, 2H), 5.10 (t, 1H, *J* = 5.3 Hz), 4.43 (d, 3H, *J* = 4.2 Hz), 4.27 (m, 3H), 3.93 (m, 1H), 2.98 (m, 2H), 1.98 (m, 1H), 1.69 (m, 1H), 1.59 (m, 1H), 1.41 (m, 1H), 0.88 (d, 3H, *J* = 6.8 Hz), 0.85 (d, 3H, *J* = 6.8 Hz); **^{13}C NMR** (101 MHz, DMSO-d_6) δ 171.3, 170.4, 158.9, 156.1, 143.9, 143.8, 140.7, 137.4, 127.7, 127.1, 126.9, 125.4, 120.1, 118.9, 65.7, 62.6, 60.1, 53.1, 46.7, 30.5, 29.5, 26.8, 19.2, 18.3.
Data in accordance with literature.[4]

(*S*)-*N*-(4-(hydroxymethyl)phenyl)-2-((*S*)-2-(2-methoxyacetamido)-3-methylbutanamido)-5-ureidopentanamide (S21)

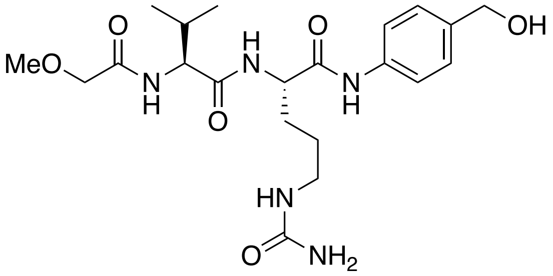

Triethylamine (463 µL, 3.32 mmol) was added to a suspension of Fmoc-Val-Cit-PABOH **S19** (100 mg, 0.166 mmol) in DMF (0.9 mL) and stirred at rt. After 18 h, excess triethylamine was removed *in vacuo* and methoxyacetyl chloride (20.4 µL, 0.216 mmol) was added to the reaction mixture, followed by triethylamine (30.1 µL, 0.216 mmol) at 0 °C. After 30 min, the reaction mixture was warmed to rt and stirred for 2 h before being quenched with H_2O (1 mL) and concentrated *in vacuo*. The resulting residue was purified by flash column chromatography (10-15% MeOH in CH_2Cl_2) to yield amide **S20** (49.5 mg, 0.110 mmol, 66%) as a white solid. **Rf** 0.19 (10% MeOH in CH_2Cl_2); **ν_{max}** (neat/cm^1) 3323 (br), 2930 (w), 1653 (s), 1607 (m), 1537 (m), 1515 (m); **^{1}H NMR** (600 MHz, DMSO-d_6) δ 10.1 (s, 1H), 8.36 (d, 1H, *J* = 7.6 Hz), 7.57 (d, 2H, *J* = 8.5 Hz), 7.51 (d, 1H, *J* = 8.9 Hz), 7.22 (d, 2H, *J* = 8.4 Hz), 6.20 (br s, 1H), 5.46 (s, 2H), 5.13 (t, 1H, *J* = 5.4 Hz), 4.41 (m, 3H), 4.30 (m, 1H), 3.87 (d, 2H, *J* = 1.2 Hz), 3.32 (s, 3H), 2.96 (m, 2H), 2.01 (m, 1H), 1.71 (m, 1H), 1.60 (m, 1H), 1.43 (m, 1H), 1.36 (m, 1H), 0.87 (d, 3H, *J* = 6.7 Hz), 0.81 (d, 3H, *J* = 6.8 Hz); **^{13}C NMR** (101 MHz, DMSO-d_6) δ 170.7, 170.4, 168.7, 159.0, 137.6, 137.4, 126.9, 118.8, 71.2, 62.6, 58.5, 56.8, 53.1, 38.4, 31.0, 29.3, 26.7, 19.2, 18.0; **HRMS** (ESI) *m/z* found $[M+H]^+$ 452.2504 $C_{21}H_{34}N_5O_6^+$, required 452.2504.

4-((*S*)-2-((*S*)-2-(2-methoxyacetamido)-3-methylbutanamido)-5-ureidopentanamido)benzyl (4-methyl-2-oxo-2*H*-chromen-7-yl)carbamate (14)

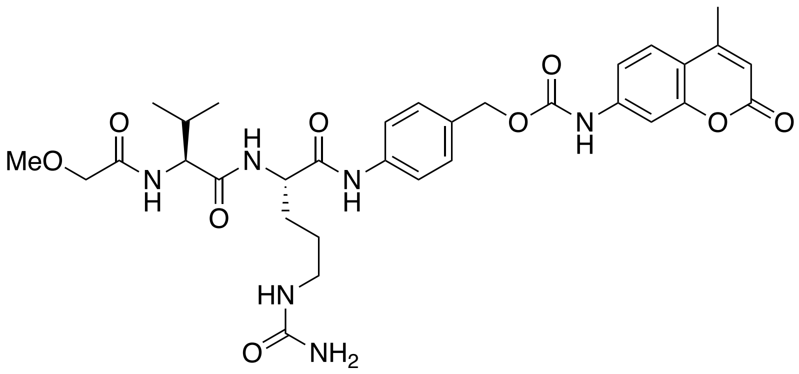

7-Amino-4-methylcoumarin (9.30 mg, 53.0 µmol) and triphosgene (7.90 mg, 27.0 µmol) were suspended in toluene (0.5 mL) and refluxed for 1 h. The reaction mixture was cooled and evaporated under a stream of nitrogen before a solution of dipeptide **S20** (20.0 mg, 44.3 µmol) in DMF (1 mL) was added. Dibutyltin dilaurate (2.60 µL, 4.40 µmol) was added to the resulting suspension and stirred at rt for 48 h before being quenched with H_2O (0.5 mL) and evaporated under a stream of nitrogen. The crude residue was purified by flash column chromatography to yield coumarin **14** (5.70 mg, 8.70 µmol, 20%) as a white solid. **Rf** 0.11 (5% MeOH in CH_2Cl_2); $\mathbf{v_{max}}$ (neat/cm^{1}) 3271 (w), 2919 (w), 1701 (m), 1635 (s), 1533 (m); **^{1}H NMR** (500 MHz, DMSO-d_6) δ 10.24 (br s, 1H), 10.10 (s, 1H), 8.34 (d, 1H, *J* = 7.5 Hz), 7.68 (d, 1H, *J* = 8.7 Hz), 7.61 (d, 2H, *J* = 8.5 Hz), 7.54 (d, 1H, *J* = 2.2 Hz), 7.47 (d, 1H, *J* = 9.0 Hz), 7.38 (m, 3H), 6.22 (d, 1H, *J* = 1.1 Hz), 6.00 (t, 1H, *J* = 5.6 Hz), 5.41 (s, 2H), 5.11 (s, 2H), 4.38 (q, 1H, *J* = 7.2 Hz), 4.29 (dd, 1H, *J* = 9.0, 6.6 Hz), 3.85 (d, 2H, *J* = 1.1 Hz), 3.00 (m, 1H), 2.93 (m, 1H), 2.37 (d, 3H, *J* = 1.0 Hz), 1.98 (m, 1H), 1.68 (m, 1H), 1.58 (m, 1H), 1.43 (m, 1H), 1.34 (m, 1H), 0.85 (d, 3H, *J* = 6.9 Hz), 0.80 (d, 3H, *J* = 6.8 Hz); **^{13}C NMR** (126 MHz, DMSO-d_6) δ 170.8, 170.6, 168.7, 160.0, 158.9, 153.8, 153.2, 142.7, 139.0, 130.8, 129.2, 126.1, 119.0, 114.4, 114.3, 111.9, 104.4, 71.2, 66.0, 58.5, 56.7, 53.2, 38.5, 31.0, 29.0, 22.1, 19.2, 18.0, 18.0; **HRMS** (ESI) *m/z* found $[M+H]^+$ 653.2916 $C_{32}H_{41}N_6O_9^+$, required 653.2930.

Scheme S 10: Synthesis of DVP-Val-Ala-MMAE 18[a]

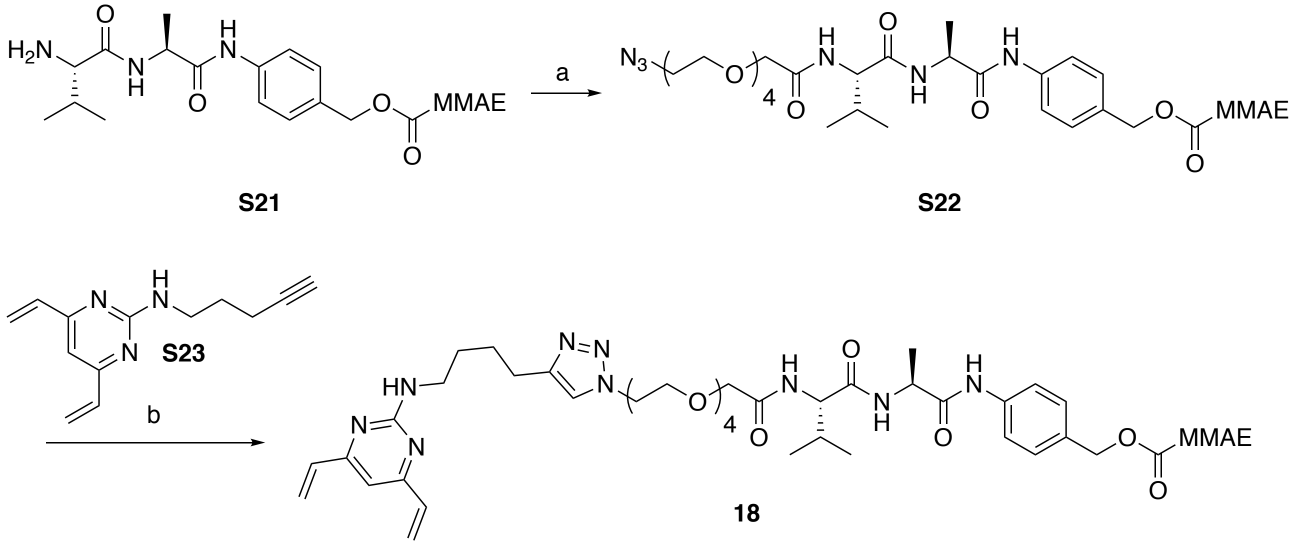

[a]Reagents and conditions: (a) N_3-PEG$_4$-COOH, HBTU, DIPEA, DMF, rt, 2 h, 70%; (b) alkyne **S23**, $CuSO_4{\cdot}5H_2O$, THPTA, sodium ascorbate, CH_2Cl_2/^tBuOH/CH_2Cl_2, rt, 13 h, 59%. Known amine **S21** was synthesised and characterised in accordance with previous literature.[5] Alkyne **S23** was synthesised and characterised in accordance with previous literature.[6]

N_3-PEG$_4$-Val-Ala-PABC-MMAE (S22)

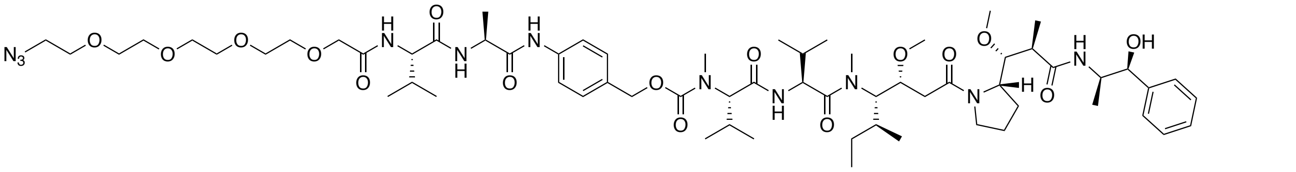

A solution of amine **S21**[5] (10.5 mg, 10.1 µmol), N_3-PEG$_4$-COOH (40.4 µL, 20.2 µmol, 0.5 M in TBME, 90%), HBTU (7.70 mg, 20.2 µmol) and DIPEA (3.50 µL, 20.2 µmol) in DMF (0.5 mL) was stirred at rt. After 2 h the solvent was removed under a stream of N_2 and the crude residue was purified by flash column chromatography (0-8% MeOH in CH_2Cl_2) to yield azide **S22** (9.10 mg, 7.02 µmol, 70%) as a white solid. **HRMS** (ESI) *m/z* found $[M+H]^+$ 1296.7771, $C_{65}H_{106}N_{11}O_{16}^+$ required 1296.7814; **HPLC** (5-95% MeCN/H_2O over 20 min) retention time 11.921 min.

DVP-PEG$_4$-Val-Ala-PABC-MMAE (18)

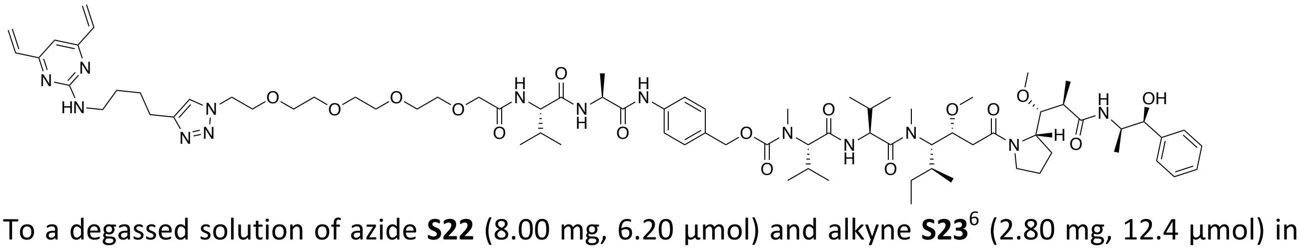

To a degassed solution of azide **S22** (8.00 mg, 6.20 µmol) and alkyne **S23**[6] (2.80 mg, 12.4 µmol) in CH_2Cl_2 (0.5 mL) was added a degassed solution of $CuSO_4{\cdot}5H_2O$ (1.90 mg, 7.40 µmol), THPTA (5.40 mg, 12.4 µmol) and sodium ascorbate (6.10 mg, 31.0 µmol) in H_2O/^tBuOH (1 mL, 1:1) and the reaction mixture stirred at rt. After 13 h, the reaction was diluted with H_2O (15 mL) and extracted with CH_2Cl_2 (5 × 15 mL). The combined organic fractions were dried ($MgSO_4$), concentrated *in vacuo* and the crude residue purified by flash column chromatography (0-8% MeOH in CH_2Cl_2) to yield linker-drug **18** (5.60 mg, 3.67 µmol, 59%) as a clear oil. **HRMS** (ESI) *m/z* found $[M+H]^+$ 1523.9174, $C_{79}H_{123}N_{14}O_{16}^+$ required 1523.9236; **HPLC** (5-95% MeCN/H_2O over 20 min) retention time 10.966 min.

Scheme S 11: Synthesis of phenyl sulfamate S24[a]

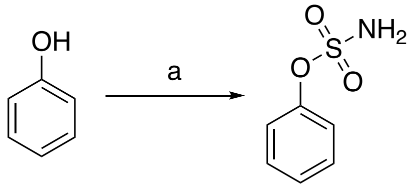

S24

[a]Reagents and conditions: (a) NaH (60%), DMF, 0 °C, 10 min then sulfamoyl chloride, 0 °C, 2 h, 58%.

Phenyl sulfamate (S24)

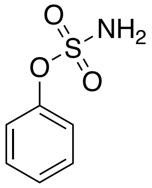

Sodium hydride (60% dispersion in mineral oil) (59.9 mg, 1.50 mmol) was added to a solution of phenol (46.8 µL, 0.500 mmol) in DMF (2 mL) at 0 °C and stirred. After 10 min, the suspension was added to a solution of sulfamoyl chloride (288 mg, 2.50 mmol) in DMF (2 mL) at 0 °C. After 2 h, the reaction was quenched with methanol, diluted with EtOAc (20 mL) and washed with H_2O (20 mL) then brine (20 mL). The organic fraction was dried (Na_2SO_4), concentrated *in vacuo* and purified by flash column chromatography (10-30% EtOAc in PE) to yield phenyl sulfamate **S24** (50.5 mg, 0.292 mmol, 58%) as a white solid. **Rf** 0.30 (30% EtOAc in PE); **^{1}H NMR** (400 MHz, $CDCl_3$) δ 7.42 (m, 2H), 7.33 (m, 3H), 5.00 (br s, 2H); **^{13}C NMR** (101 MHz, $CDCl_3$) δ 150.2, 130.1, 127.5, 122.2. Data in accordance with literature.[7]

Bioconjugation

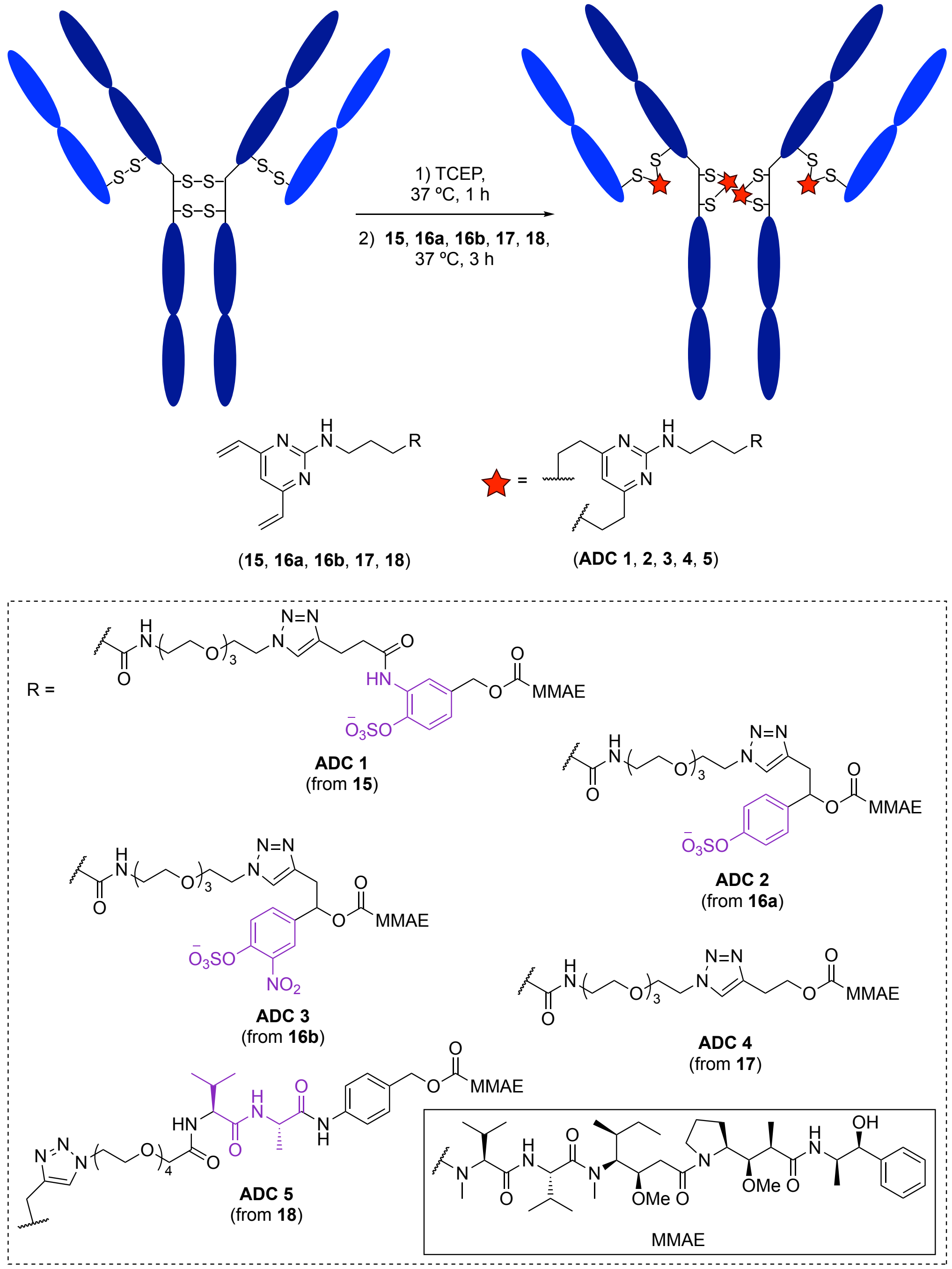

To a solution of trastuzumab (40 µL, 16.9 µM, 2.5 mg/mL) in TBS (25 mM Tris HCl pH 8, 25 mM NaCl, 0.5 mM EDTA) was added TCEP (10 eq.). The mixture was vortexed and incubated at 37 °C for 1 h with shaking at 1000 rpm. A solution of linker-payload **13-16** (20 mM in DMSO) was added with additional DMSO (final concentration of 0.637 mM, 40 eq., 10% DMSO (v/v)) and the reaction mixture incubated at 37 °C for 3 h with shaking at 1000 rpm. The excess reagents were removed by size-exclusion chromatography with a Zeba Spin desalting column (40K MWCO, 0.5 mL) and exchanged into PBS with an Amicon-Ultra centrifugal filter (10K MWCO, Merck Millipore). LC-MS and SDS-PAGE analysis demonstrated >95% conversion to the desired conjugate.

LC-MS Analysis

a)

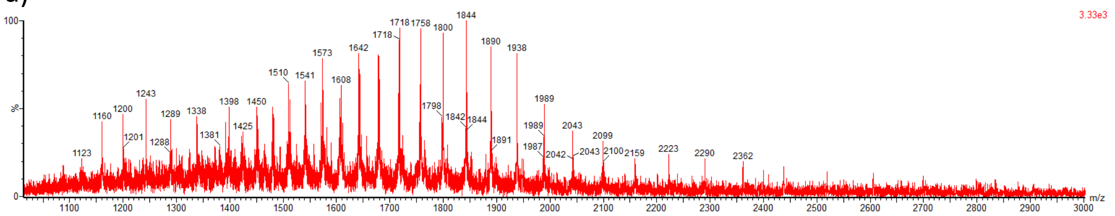

b)

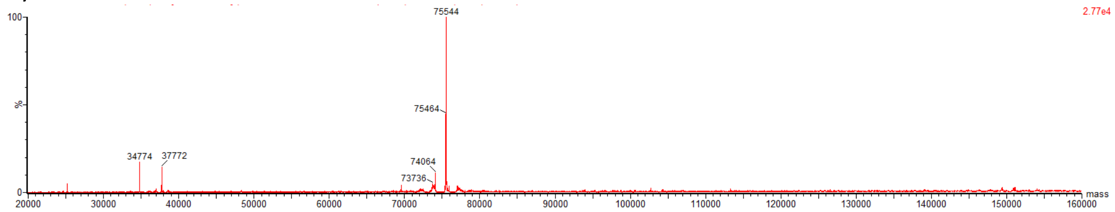

c)

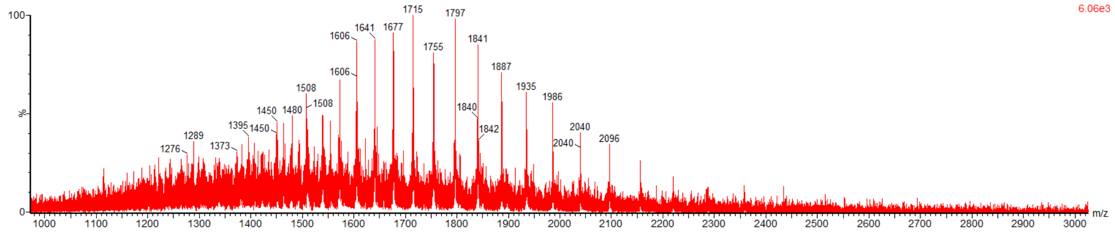

d)

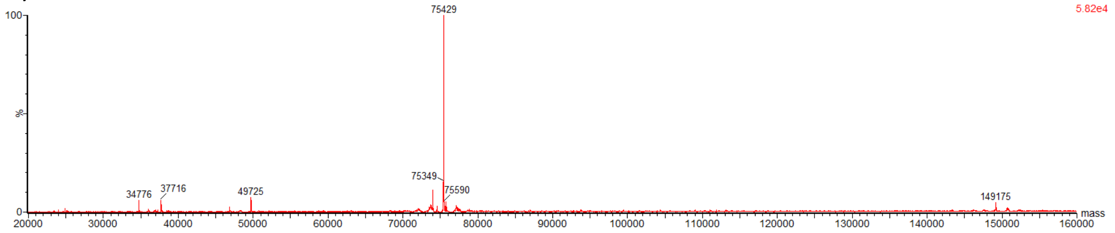

e)

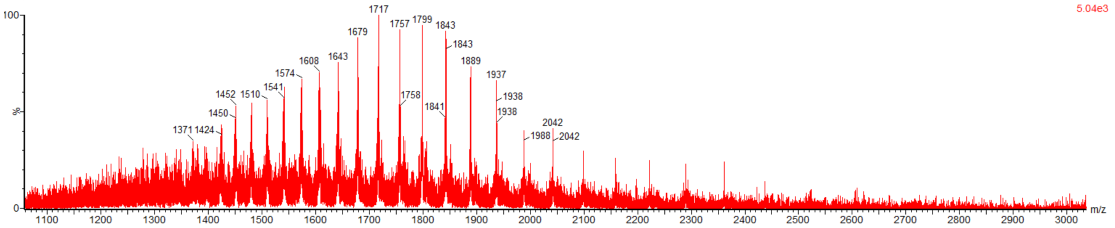

f)

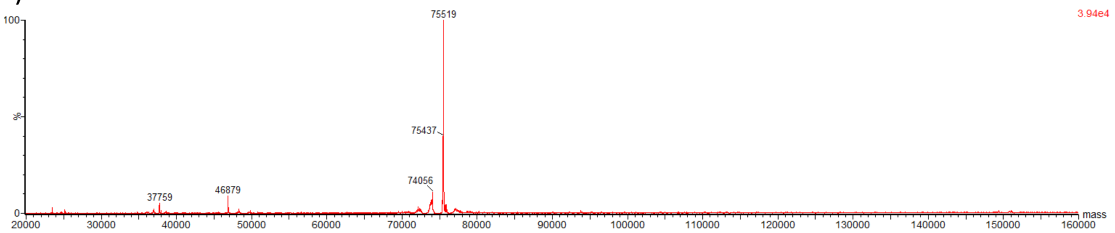

g)

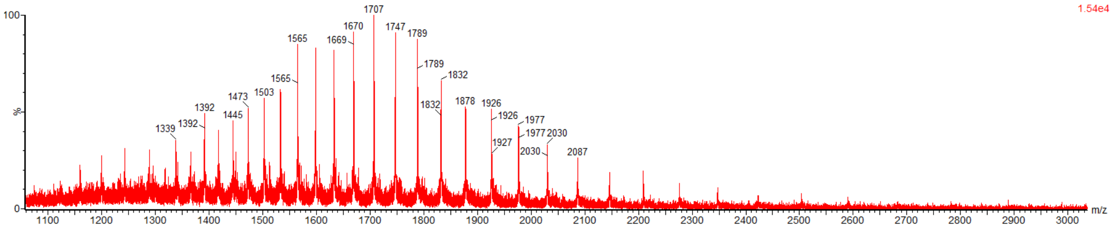

h)

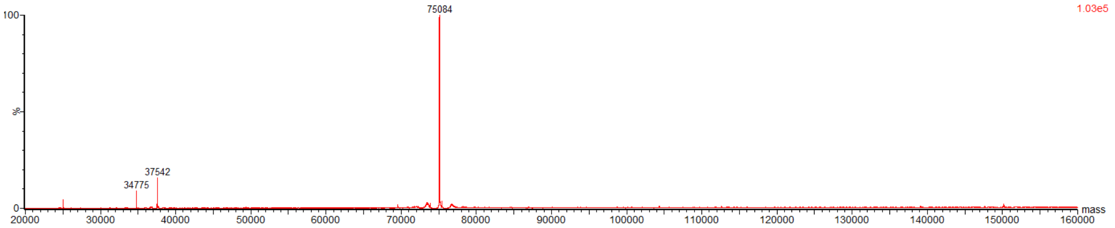

i)

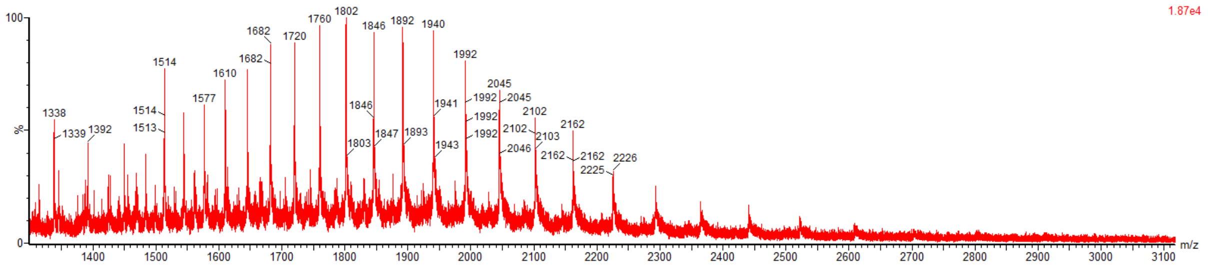

j)

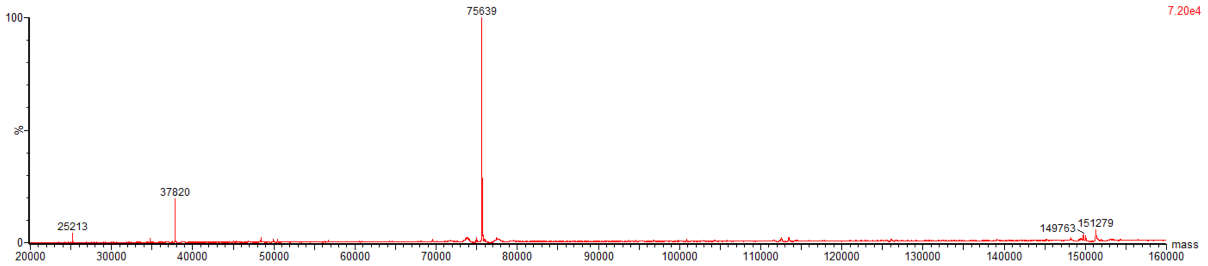

Fig. S 1: LC-MS analysis of ADCs 1-5.

(a) non-deconvoluted MS of ADC **1**; (b) deconvoluted MS of ADC **1**, expected 75,538 Da, observed 75,544 Da; (c) non-deconvoluted MS of ADC **2**; (d) deconvoluted MS of ADC **2**, expected 75,423 Da, observed 75,429 Da; (e) non-deconvoluted MS of ADC **3**; (f) deconvoluted MS of ADC **3**, expected 75,513 Da, observed 75,519 Da; (g) non-deconvoluted MS of ADC **4**; (h) deconvoluted MS of ADC **4**, expected 75,083 Da, observed 75,084 Da; (i) non-deconvoluted MS of ADC **5**; (j) deconvoluted MS of ADC **5**, expected 75,634 Da, observed 75,639 Da. Note: A minor peak ≈80 Da less than the major peak appears for ADC **1**-**3**. This peak does not appear prior to deglycosylation and is an artefact of the deglycosylation process.

SDS-PAGE Analysis

SDS-PAGE analysis with 12% acrylamide gel under reducing conditions reveals the major product of bioconjugation to be half-antibody, with a molecular weight between that of the full trastuzumab antibody and the heavy chain.

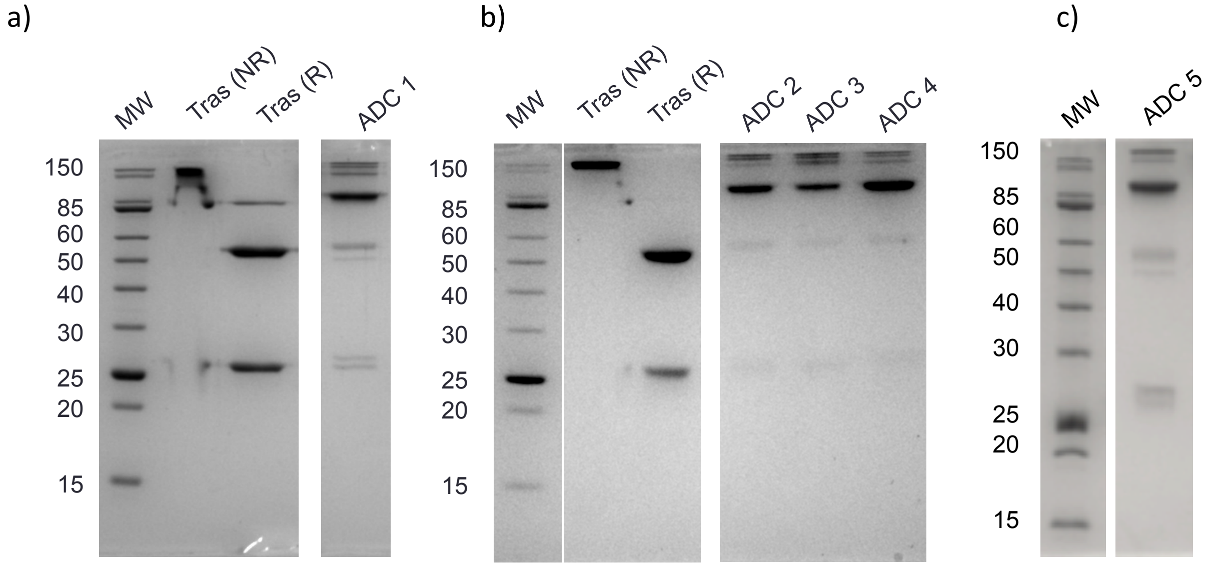

Fig. S 2: SDS-PAGE analysis of ADC 1-5 in 12% acrylamide gel.

Lane markings: Tras (NR) = trastuzumab, non-reduced, Tras (R) = trastuzumab, reduced with β-mercaptoethanol at 70 °C for 2 min. a) Analysis of ADC **1**, reduced with β-mercaptoethanol at 70 °C for 2 min, b) analysis of ADC **2**, **3**, and **4** all reduced with β-mercaptoethanol at 70 °C for 2 min and c) analysis of ADC **5** reduced with β-mercaptoethanol at 70 °C for 2 min.

Sulfatase Hydrolysis

The enzyme units refer to the enzyme's ability to hydrolyse *p*-nitrocatechol sulfate.

Sulfatase from *Helix pomatia* with 7 and 12

Sulfatase (*Helix pomatia*, EC 3.1.6.1, 12 µL, 20 U/mL in 34 mM NaCl (aq)) was added to a vortexed solution of linker-AMC (2.4 µL, 250 µM in DMSO) in NaOAc buffer (24 µL, 0.5 M, pH 5) and H_2O (81.6 µL). 110 µL of the resulting solution was added to a 384 well plate (Greiner, black, clear bottomed) and the fluorescence intensity was measured over 12 h at 37 °C, with readings taken at 2 minute intervals. An adhesive film (Bio-Rad) was used to prevent solvent evaporation. The reactions were performed in triplicate and the plotted values are the normalised mean values. Data were processed using GraphPad Prism Version 7.

Sulfatase from *Helix pomatia* and phenyl sulfamate with 7 and 12

Phenyl sulfamate (0.3 µmol), sulfatase (*Helix pomatia*, EC 3.1.6.1, 12 µL, 20 U/mL in 34 mM NaCl (aq)) and NaOAc buffer (24 µL, 0.5 M, pH 5) were added to H_2O (81.6 µL) and incubated at 37 °C for 3 h. Linker-AMC (2.4 µL, 250 µM in DMSO) was then added and 110 µL of the thoroughly mixed resulting solution was added to a 384 well plate (Greiner, black, clear bottomed) and the fluorescence intensity was measured over 12 h at 37 °C, with readings taken at 2 minute intervals. An adhesive film (Bio-Rad) was used to prevent solvent evaporation. The reactions were performed in triplicate and the plotted values are the normalised mean values. Data were processed using GraphPad Prism Version 7. The same reaction was also performed in tandem in the absence of phenyl sulfamate as a control.

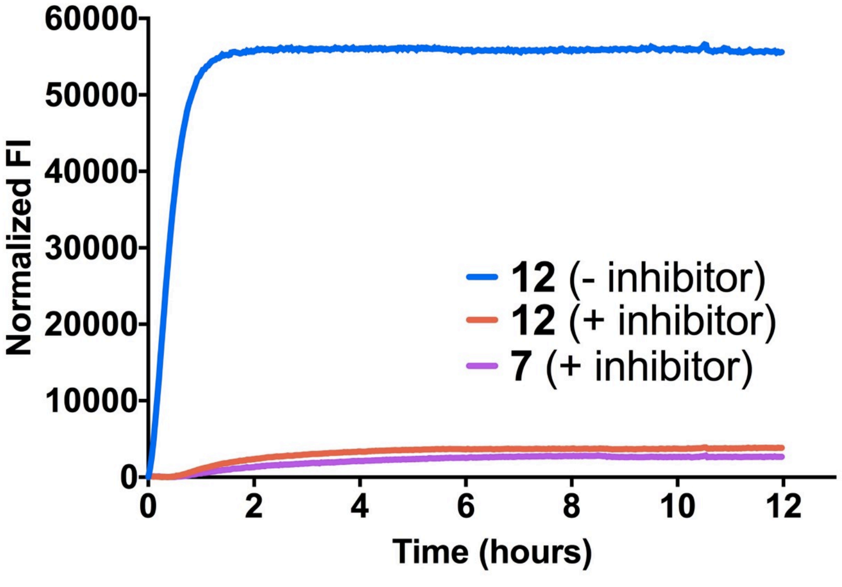

Fig. S 3: Incubation of 7 and 12 with sulfatase and 2.5 mM phenyl sulfamate inhibitor. 12 with sulfatase in the absence of phenyl sulfamate (blue line) is also included.

Sulfatase from *Helix pomatia* with 12 at pH 5, 7.4 and 9

Sulfatase (*Helix pomatia*, EC 3.1.6.1, 12 µL, 20 U/mL in 34 mM NaCl (aq)) was added to a vortexed solution of linker-AMC (2.4 µL, 250 µM in DMSO) in buffer (24 µL, 0.5 M) and H_2O (81.6 µL). The concentrated buffer solutions were NaOAc at pH 5, NaP_i at pH 7.4 and Tris at pH 9. 110 µL of the resulting solutions were added to a 384 well plate (Greiner, black, clear bottomed) and the fluorescence intensity was measured over 12 h at 37 °C, with readings taken at 2 minute intervals.

An adhesive film (Bio-Rad) was used to prevent solvent evaporation. The reactions were performed in triplicate and the plotted values are the normalised mean values. Data were processed using GraphPad Prism Version 7.

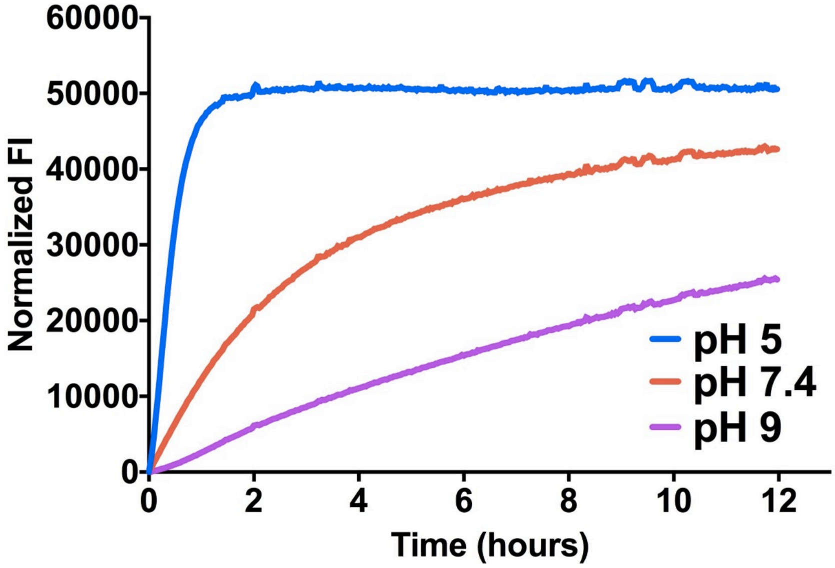

Fig. S 4: Incubation of 12 with sulfatase at pH 5, 7.4 and 9.

Arylsulfatase A (ARSA) and arylsulfatase B (ARSB) with 12

Arylsulfatase A: Sulfatase (Human recombinant arylsulfatase A, EC 3.1.6.8, 14.2 µL, 8.43 µM in 34 mM NaCl (aq)) was added to a vortexed solution of linker-AMC (2.4 µL, 250 µM in DMSO) in NaOAc buffer (24 µL, 0.5 M, pH 5) and H_2O (79.4 µL).

Arylsulfatase B: Sulfatase (Human recombinant arylsulfatase B, EC 3.1.6.12, 18.0 µL, 6.67 µM in 34 mM NaCl (aq)) was added to a vortexed solution of linker-AMC (2.4 µL, 250 µM in DMSO) in NaOAc buffer (24 µL, 0.5 M, pH 5) and H_2O (79.4 µL).

110 µL of the resulting solutions were added to a 384 well plate (Greiner, black, clear bottomed) and the fluorescence intensity was measured over 12 h at 37 °C, with readings taken at 2 minute intervals. An adhesive film (Bio-Rad) was used to prevent solvent evaporation. The plotted values are the normalised values. Data were processed using GraphPad Prism Version 7.

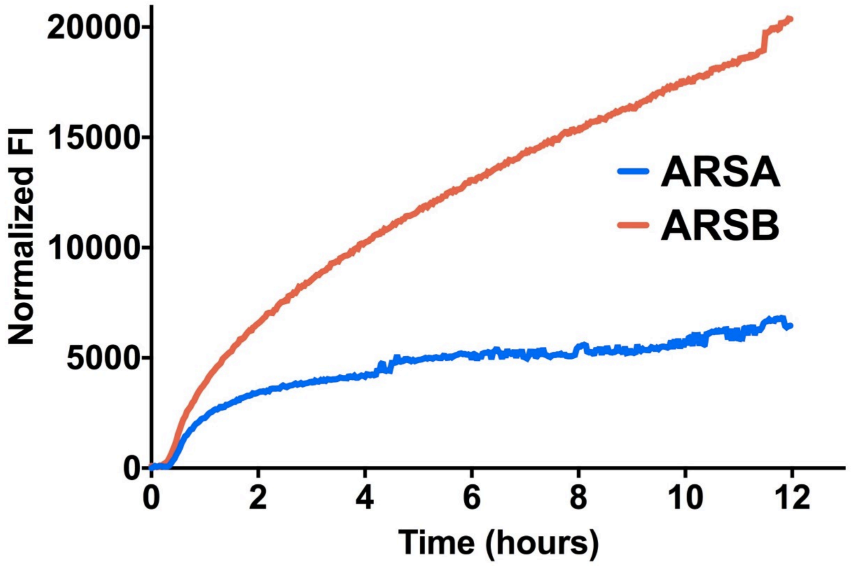

Fig. S 5: Incubation of 12 with ARSA and ARSB.

Stability studies

Plasma stability studies were conducted with a concentration of 55% plasma, to replicate its proportion in whole blood.

Mouse plasma stability over 8 hours

Mouse plasma (110 µL) was added to a vortexed solution of linker-AMC (4.0 µL, 250 µM in DMSO) in PBS (96 µL). 190 µL of the resulting solution was added to a 96 well plate (Greiner, black, clear bottomed). The fluorescence intensity was measured over 8 h at 37 °C, with readings taken at 1 minute intervals. An adhesive film (Bio-Rad) was used to prevent solvent evaporation. The reactions were performed in triplicate and the plotted values are the normalised mean values. Data were processed using GraphPad Prism Version 7.

Human/mouse plasma stability over 7 days

Human/mouse plasma (154 µL) was added to a vortexed solution of linker-AMC (5.6 µL, 250 µM in DMSO) in PBS (120.4 µL). The solution was incubated at 37 °C and 40 µL aliquots were taken at t = 0, 1, 3, 5 and 7 days. The aliquots were added to a 384 well plate (Corning, black, low volume) and the fluorescence intensity was measured. The reactions were performed in duplicate and the plotted values are the normalised mean values. Data were processed using GraphPad Prism Version 7.

Glutathione stability

Linker-AMC (2.4 µL, 250 µM in DMSO) was added to a solution of reduced glutathione (0.5 mM) in NaP$_i$ buffer (117.6 µL, 100 mM, pH 7.4). 110 µL of the resulting solution was added to a 384 well plate (Greiner, black, clear bottomed) and the fluorescence intensity was measured over 12 h at 37 °C, with readings taken at 2 minute intervals. An adhesive film (Bio-Rad) was used to prevent solvent evaporation. The reactions were performed in triplicate and the plotted values are the normalised mean values. Data were processed using GraphPad Prism Version 7.

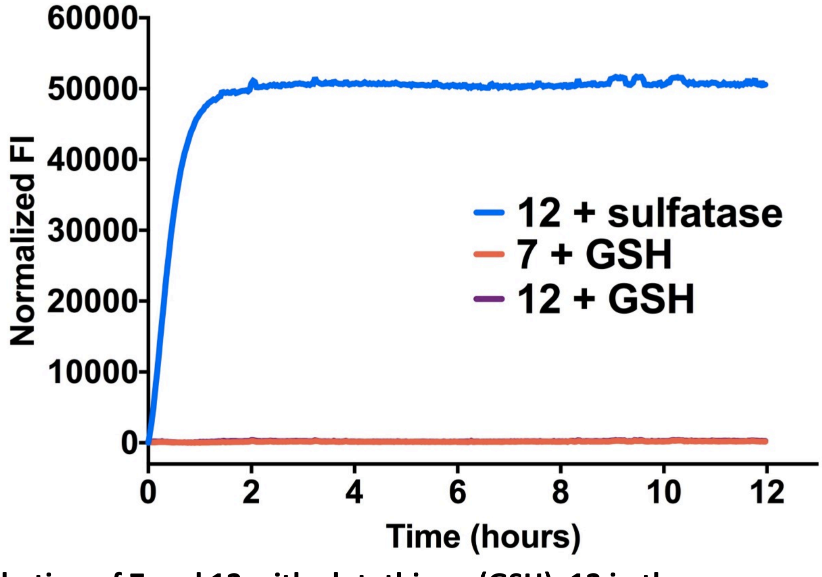

Fig. S 6: Incubation of 7 and 12 with glutathione (GSH). 12 in the presence of sulfatase (blue line) is included as a comparison.

Cytotoxicity of ADCs 1-5 in HER2-positive SKRB3 and HER2-negative T47D cells

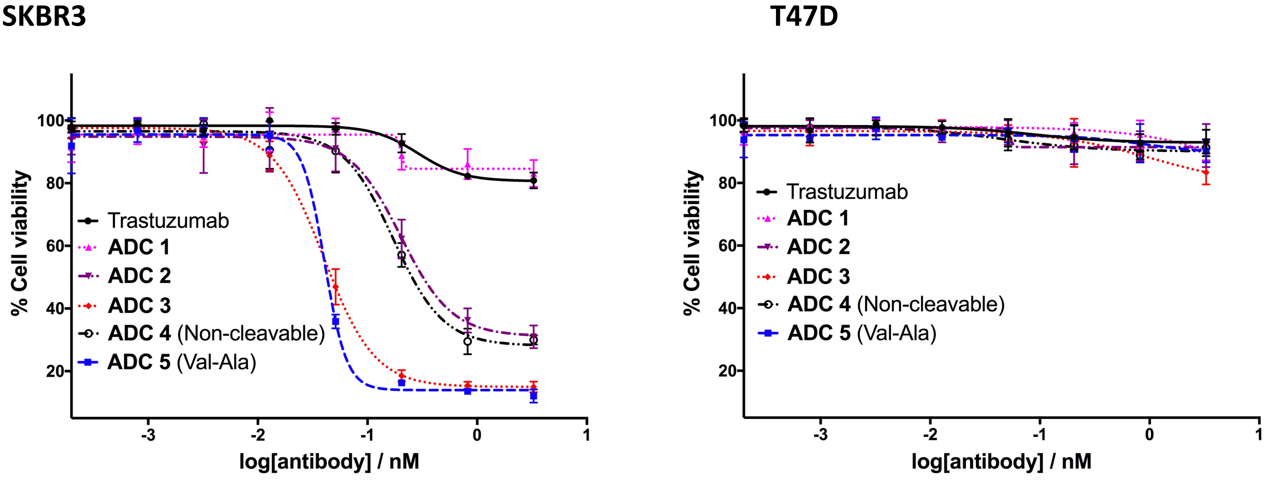

Fig. S 7: Cytotoxicity of ADCs 1-5 in SKBR3 and T47D cells

IC_{50} Values of ADCs 1-5 in HER2+ Cells

Table S 1: The calculated IC_{50} values for ADCs 2-5 in BT474 and SKBR3 cell lines.

Compound	IC_{50} (pM)	
	BT474	SKBR3
**ADC 1**	N/A	N/A
**ADC 2**	111	200
**ADC 3**	61	40
**ADC 4**	609	171
**ADC 5**	92	41

Cells Lines

HER2-positive SKBR3 and BT474 cells were obtained from the American Type Culture Collection (ATCC) and HER2-negative MCF7 and T47D cells were obtained from the European Collection of Authenticated Cell Cultures (ECACC) and ATCC, respectively. SKBR3 cells were maintained in high glucose McCoy's 5A medium, supplemented with 10% heat-inactivated foetal-bovine serum (FBS), 50 U/mL penicillin and 50 µg/mL streptomycin. MCF7 cells were maintained in Dulbecco's Modified Eagle Medium (DMEM) supplemented with 10% heat-inactivated fetal-bovine serum (FBS), 2 mM L-glutamine, 50 U/mL penicillin and 50 µg/mL streptomycin. BT474 and T47D cell lines were maintained in RPMI1640 medium supplemented with 10% heat-inactivated fetal-bovine serum (FBS), 2 mM L-glutamine, 50 U/mL penicillin and 50 µg/mL streptomycin. All cell lines were incubated at 37 °C with 5% CO_2.

Cell Viability

Cells were seeded in 96-well plates for 24 h at 37 °C with 5% CO_2. SKBR3 cells were seeded at 15,000 cells/well, BT474 cells were seeded at 20,000 cells/well, MCF7 cells were seeded at 7,500 cells/well and T47D cells were seeded at 10,000 cells/well. Serial dilutions of ADCs **1-5** and trastuzumab were added to the cells in complete growth medium and incubated at 37 °C with 5%

CO_2 for 96 h. Cell viability was measured using CellTiter-Glo viability assay (Promega) according to the manufacturer's instructions. Cell viability was plotted as a percentage of untreated cells. Each measurement was taken in triplicate and three independent repeats were performed.

Bibliography

1 L. S. Simpson and T. S. Widlanski, *J. Am. Chem. Soc.*, 2006, **128**, 1605–1610.

2 S. Harper, B. Pacini, S. Avolio, M. Di Filippo, G. Migliaccio, R. Laufer, R. De Francesco, M. Rowley and F. Narjes, *J. Med. Chem.*, 2005, **48**, 1314–1317.

3 T. Legigan, J. Clarhaut, I. Tranoy-Opalinski, A. Monvoisin, B. Renoux, M. Thomas, A. Le-Pape, S. Lerondel and S. Papot, *Angew. Chemie - Int. Ed.*, 2012, **51**, 11606–11610.

4 US 2010/0233190 A1, 2010.

5 WO/2016/064749, 2016.

6 S. J. Walsh, S. Omarjee, W. R. J. D. Galloway, T. T. L. Kwan, H. F. Sore, J. S. Parker, M. Hyvönen, J. S. Carroll and D. R. Spring, *Chem. Sci.*, 2019, **10**, 694–700.

7 J. L. Roizen, D. N. Zalatan and J. Du Bois, *Angew. Chemie - Int. Ed.*, 2013, **52**, 11343–11346.

NMR Spectra and HPLC Traces

Methyl 3-amino-4-hydroxybenzoate (2)

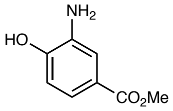

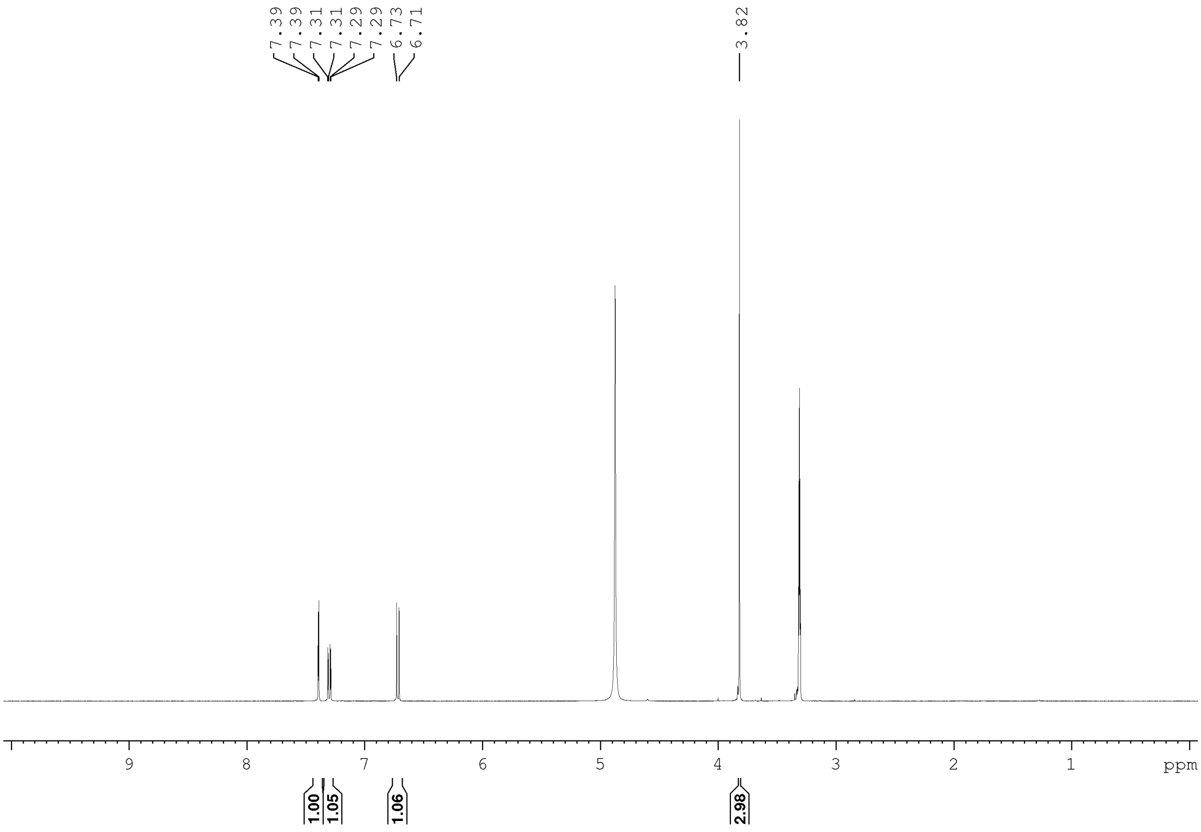

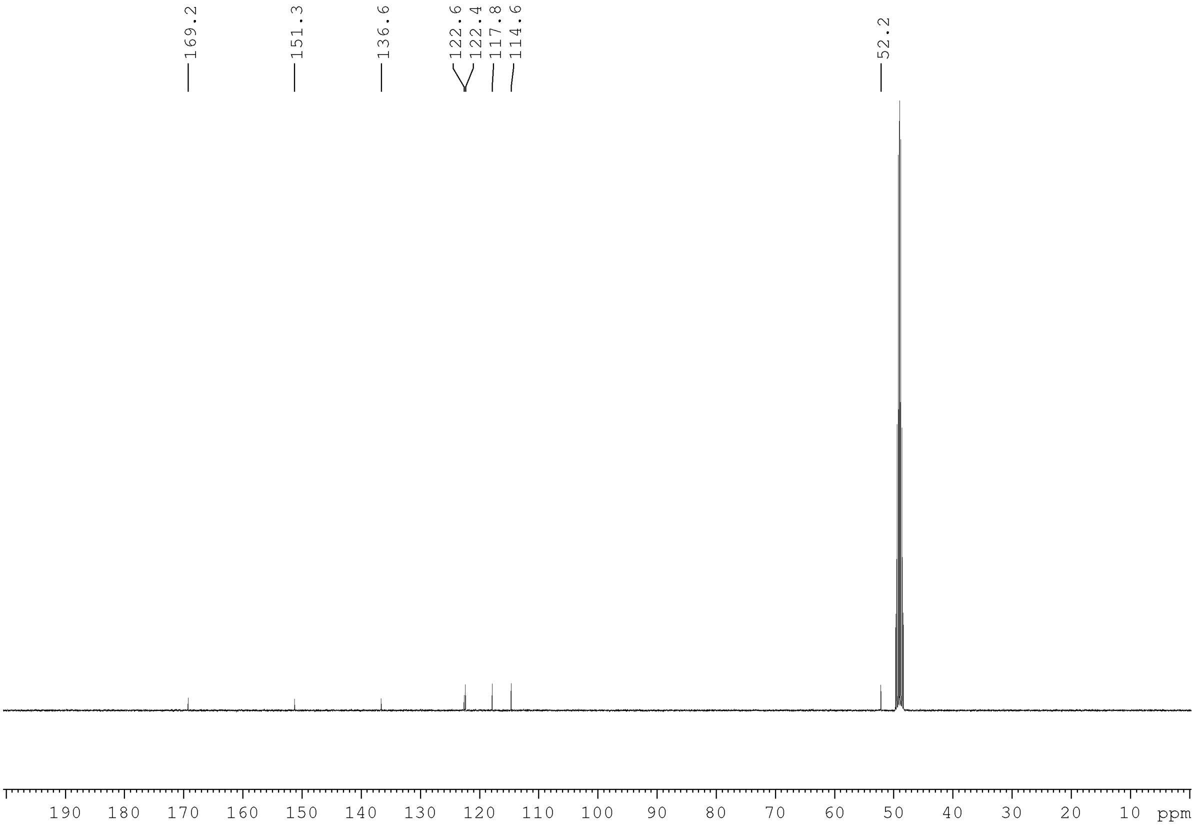

Methyl 4-hydroxy-3-(pent-4-ynamido)benzoate (3)

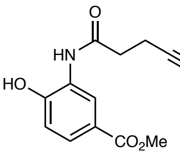

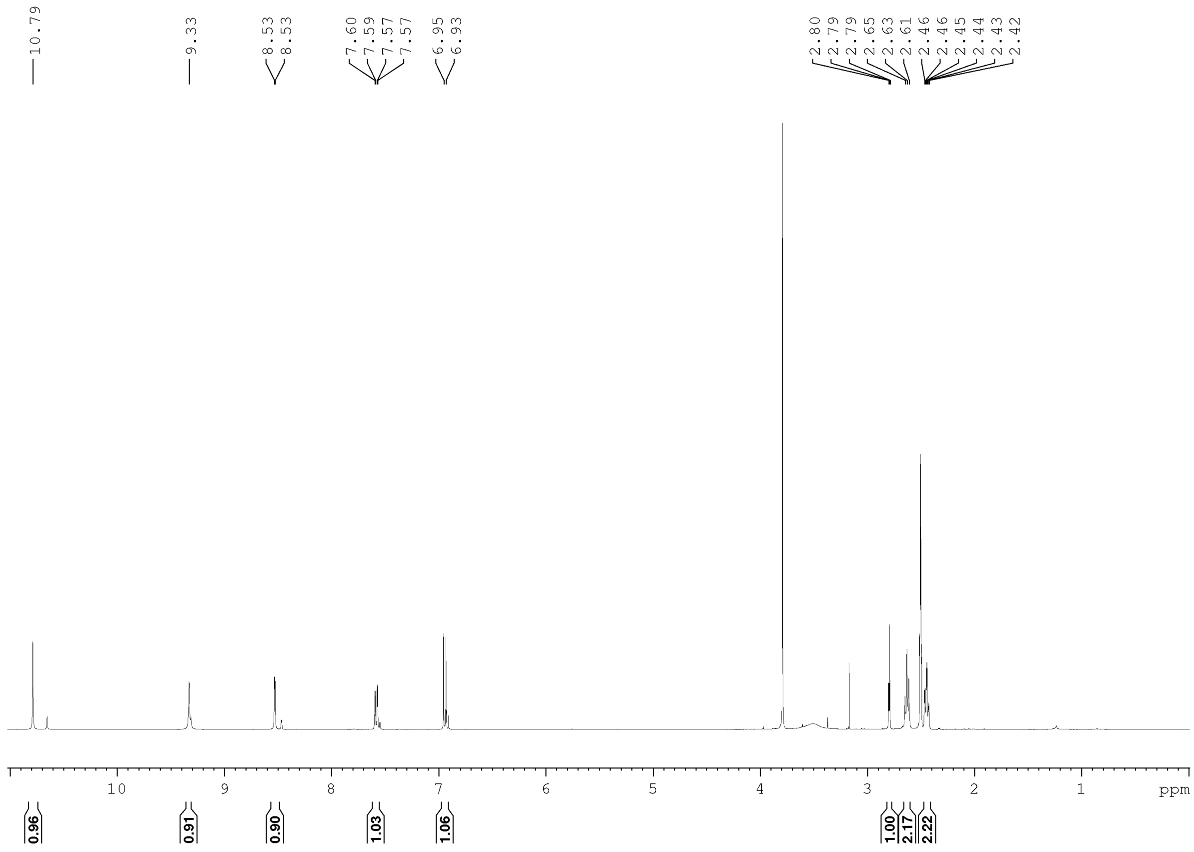

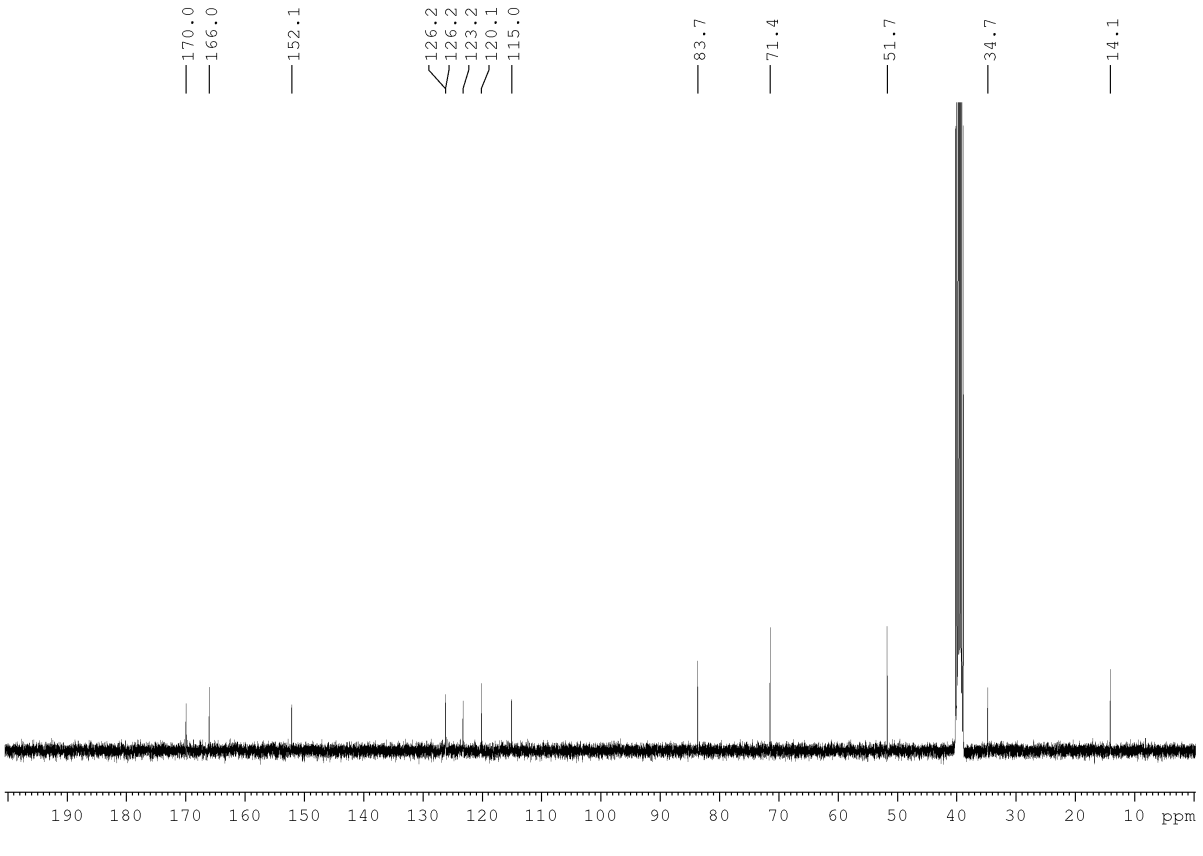

Methyl 4-(((4-nitrophenoxy)sulfonyl)oxy)-3-(pent-4-ynamido)benzoate (4)

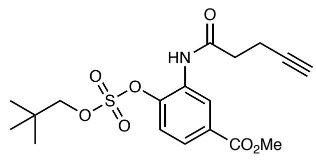

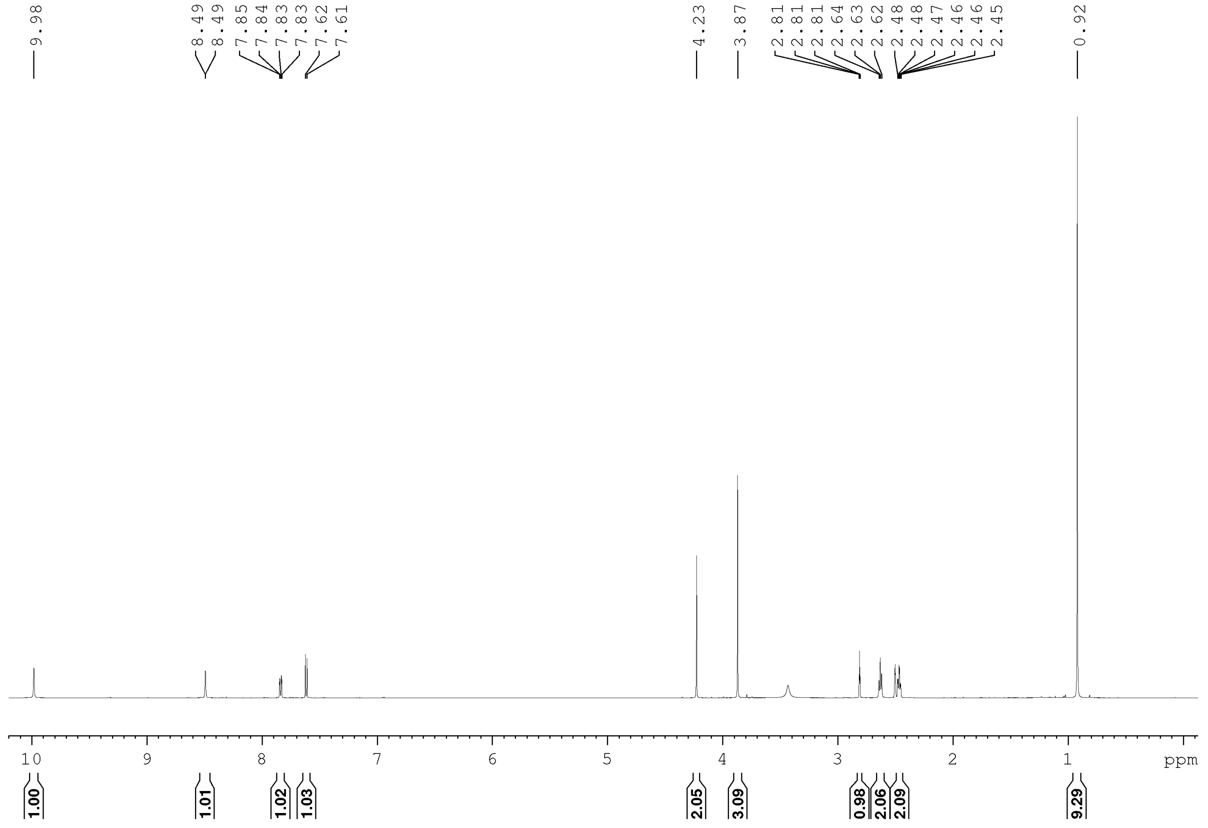

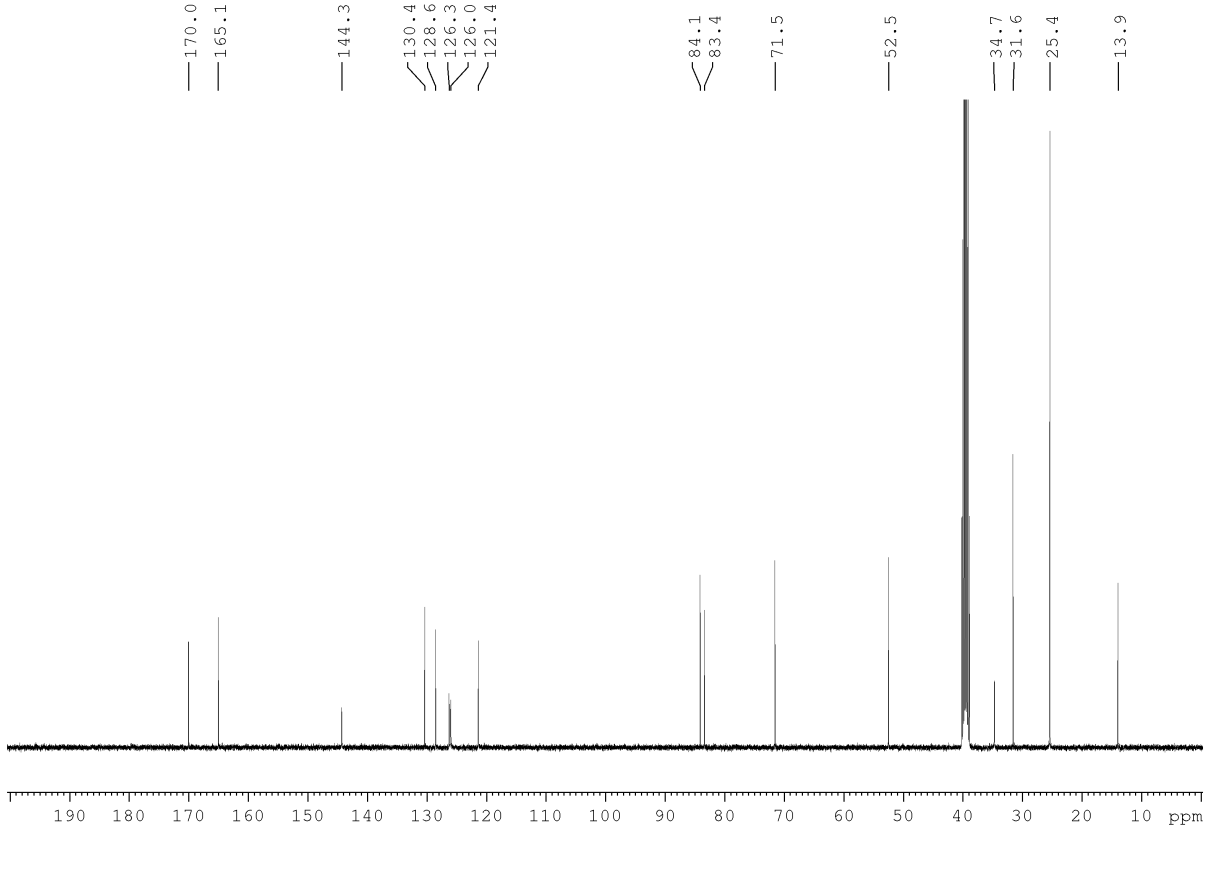

4-(hydroxymethyl)-2-(pent-4-ynamido)phenyl neopentyl sulfate (5)

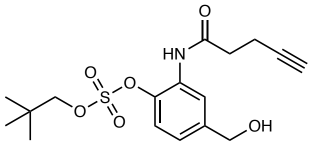

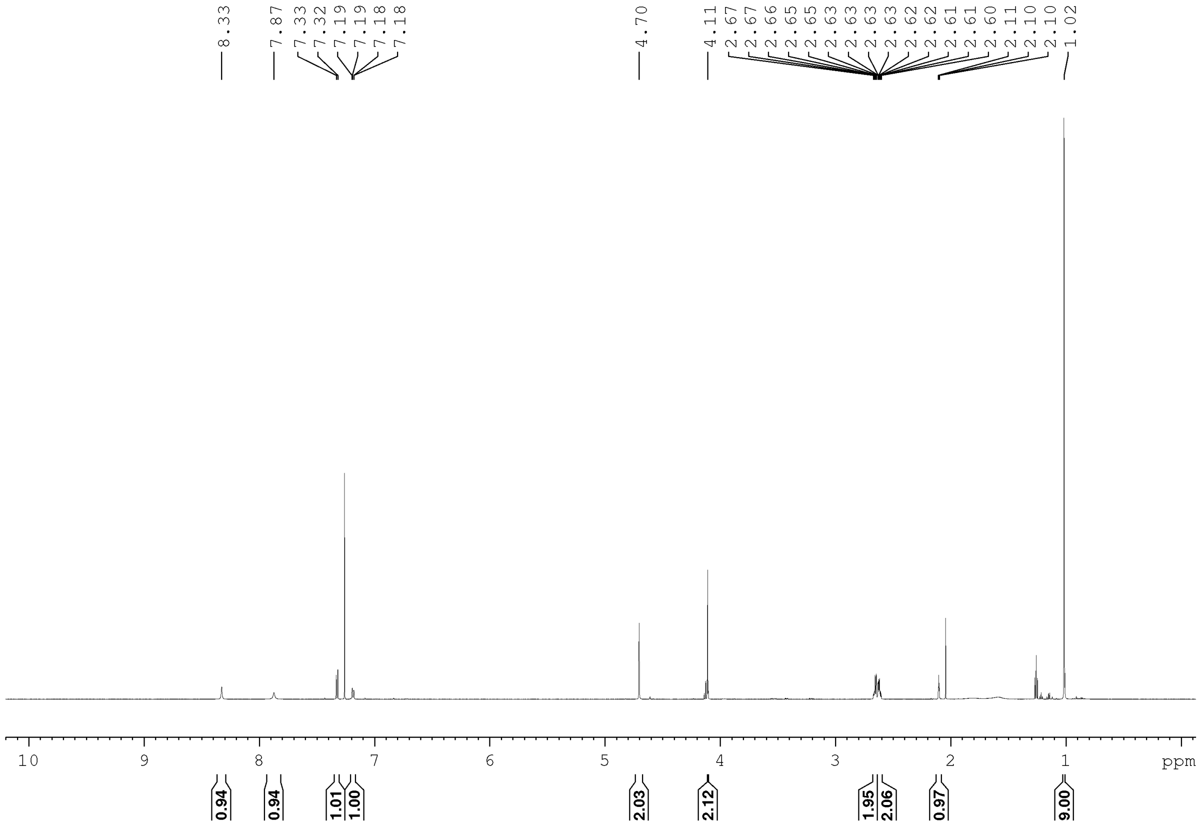

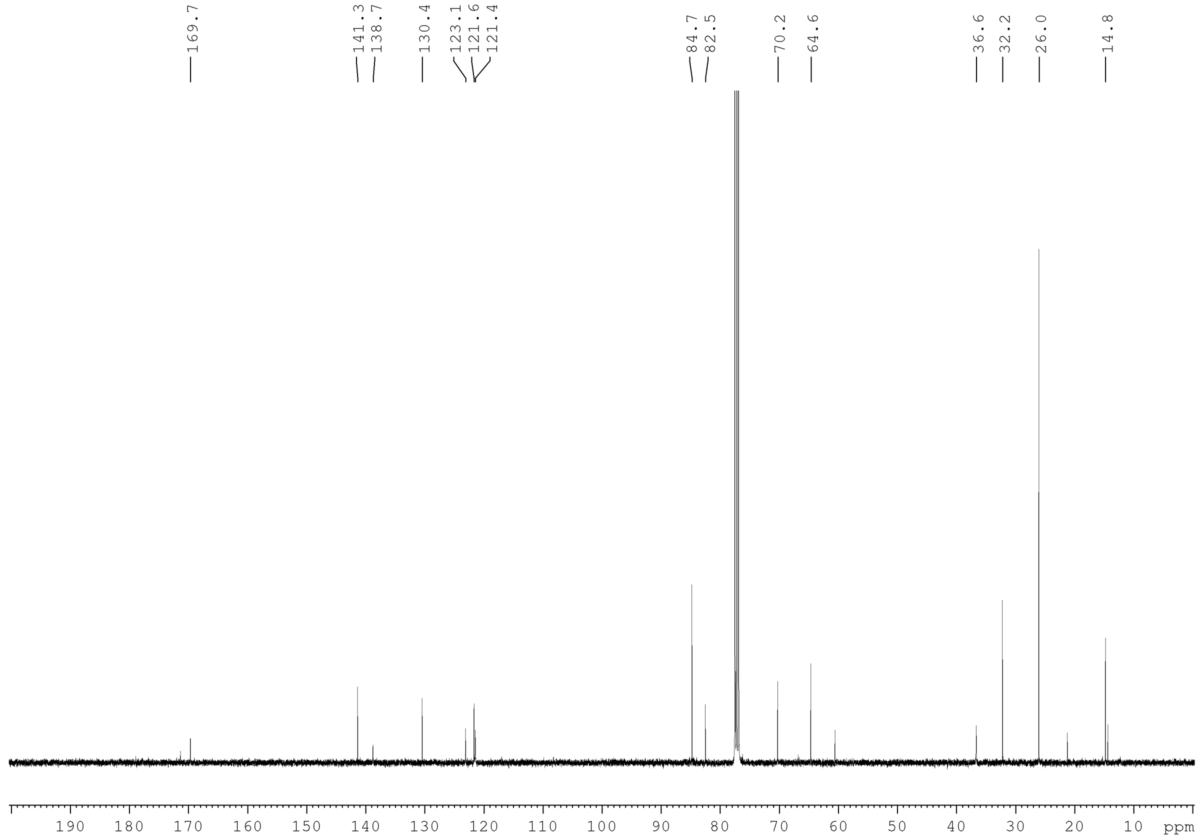

4-((((4-methyl-2-oxo-2*H*-chromen-7-yl)carbamoyl)oxy)methyl)-2-(pent-4-ynamido)phenyl (4-nitrophenyl) sulfate (6)

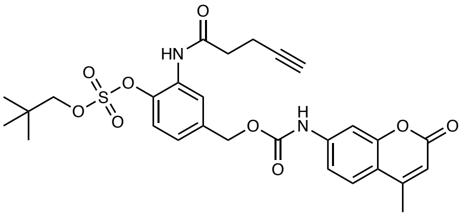

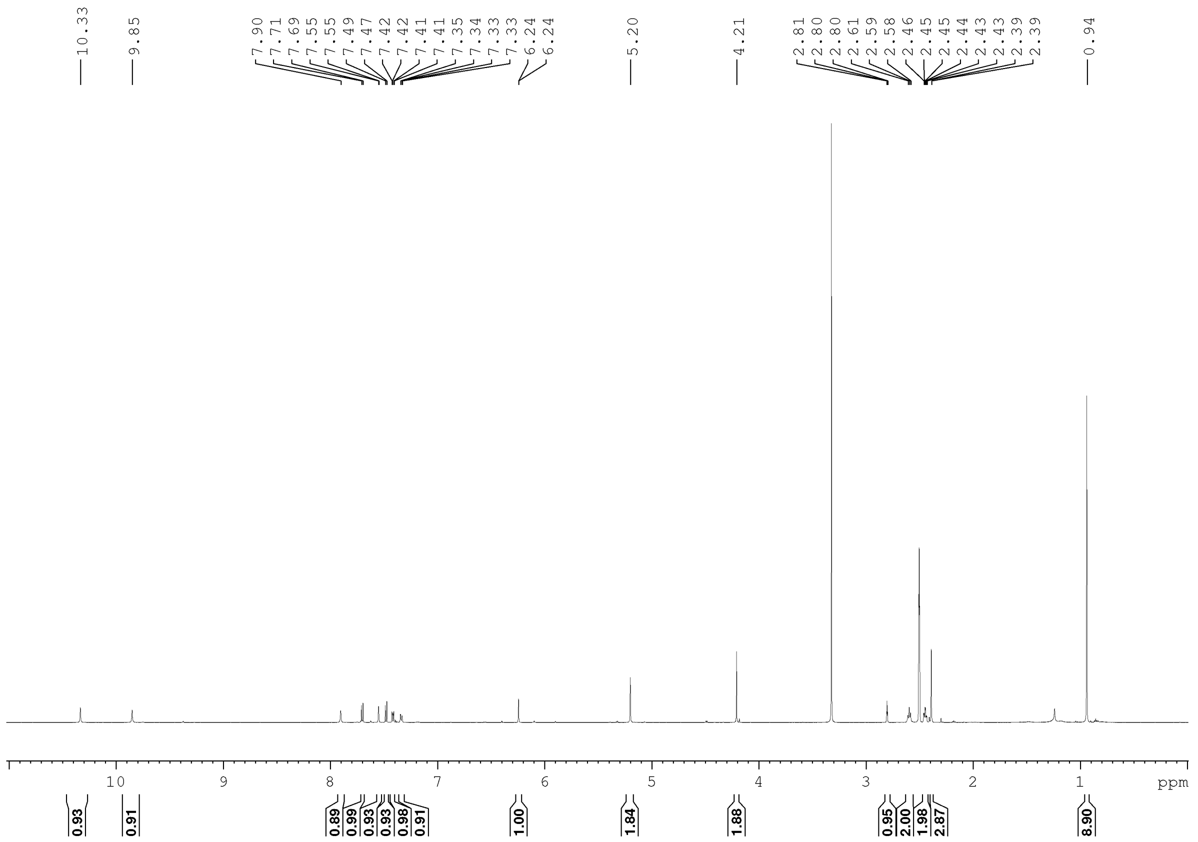

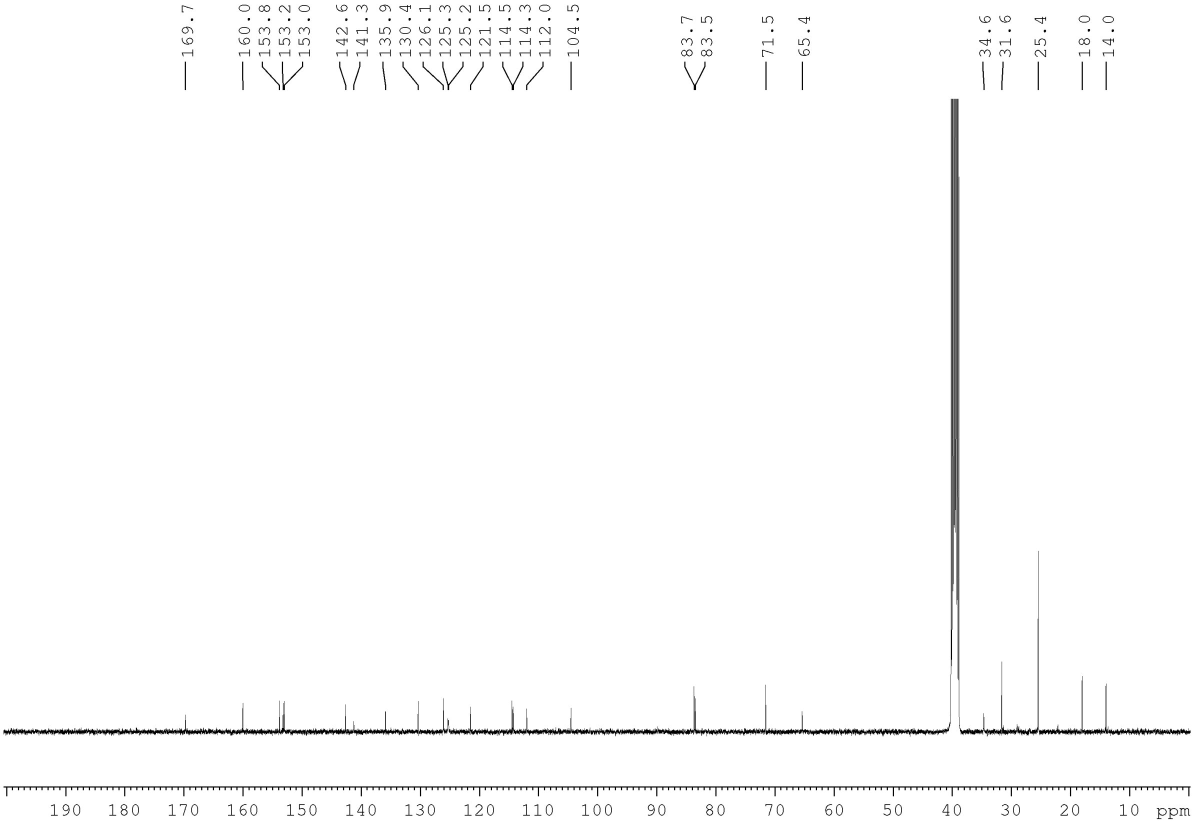

Ammonium 4-((((4-methyl-2-oxo-2*H*-chromen-7-yl)carbamoyl)oxy)methyl)-2-(pent-4-ynamido)phenyl sulfate (7)

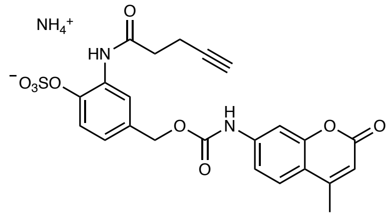

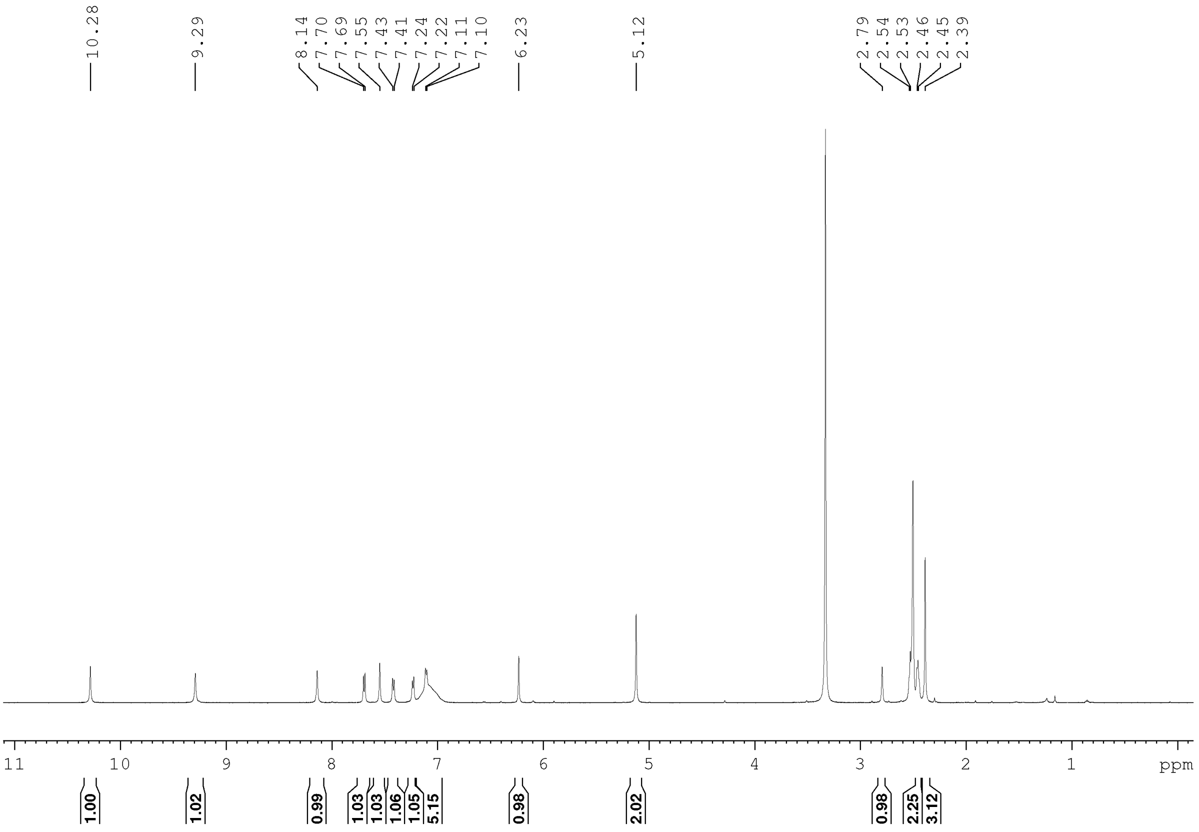

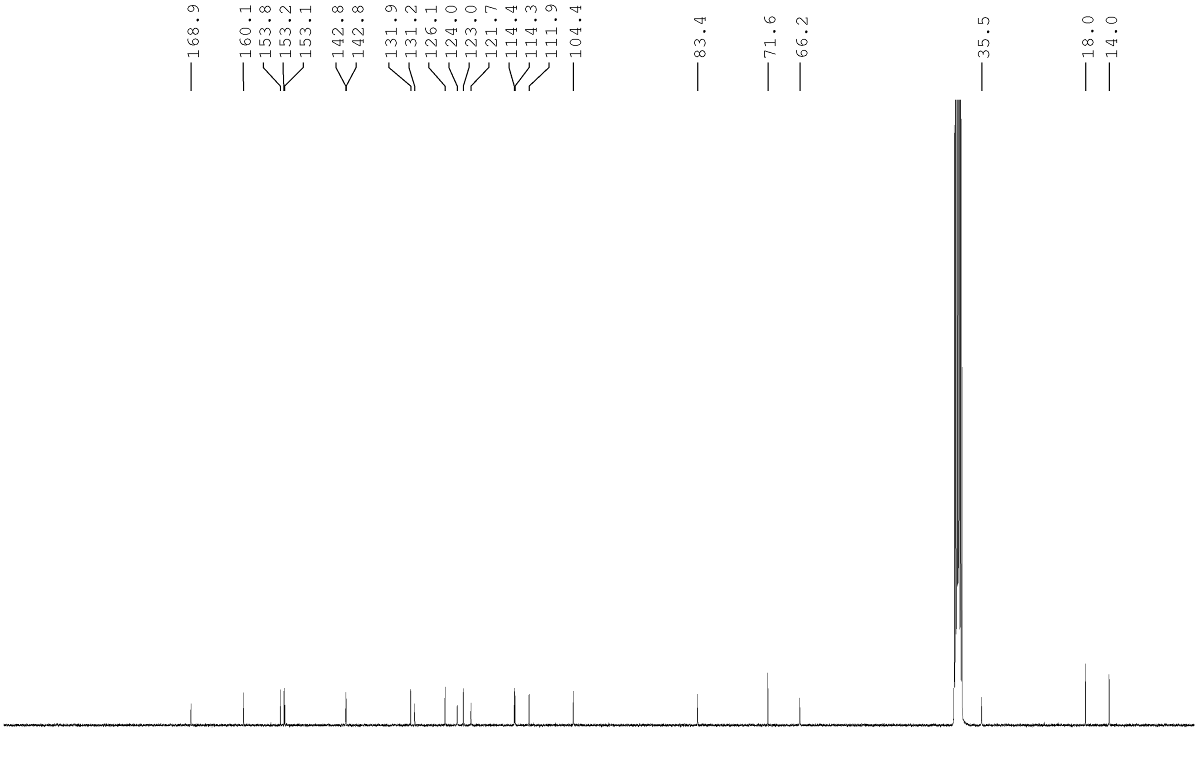

4-formylphenyl neopentyl sulfate (9)

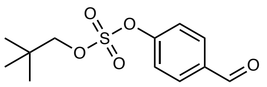

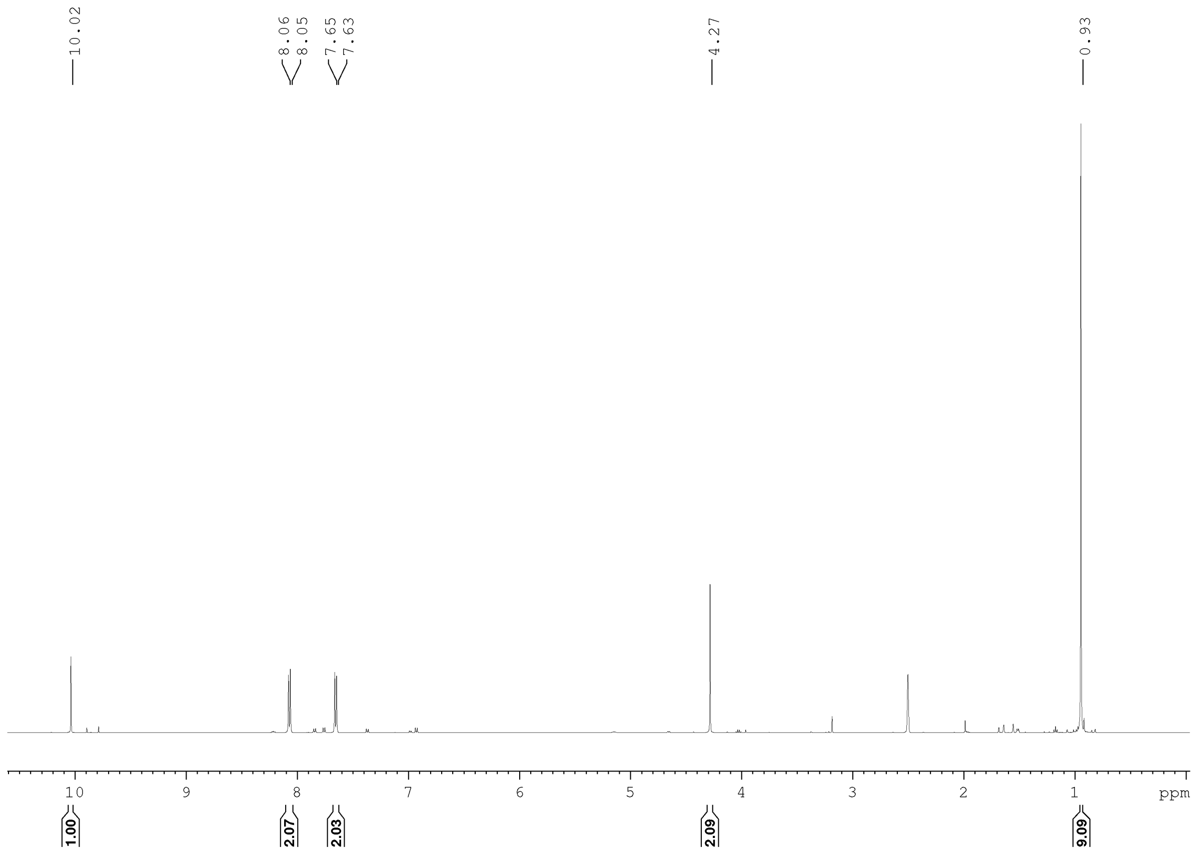

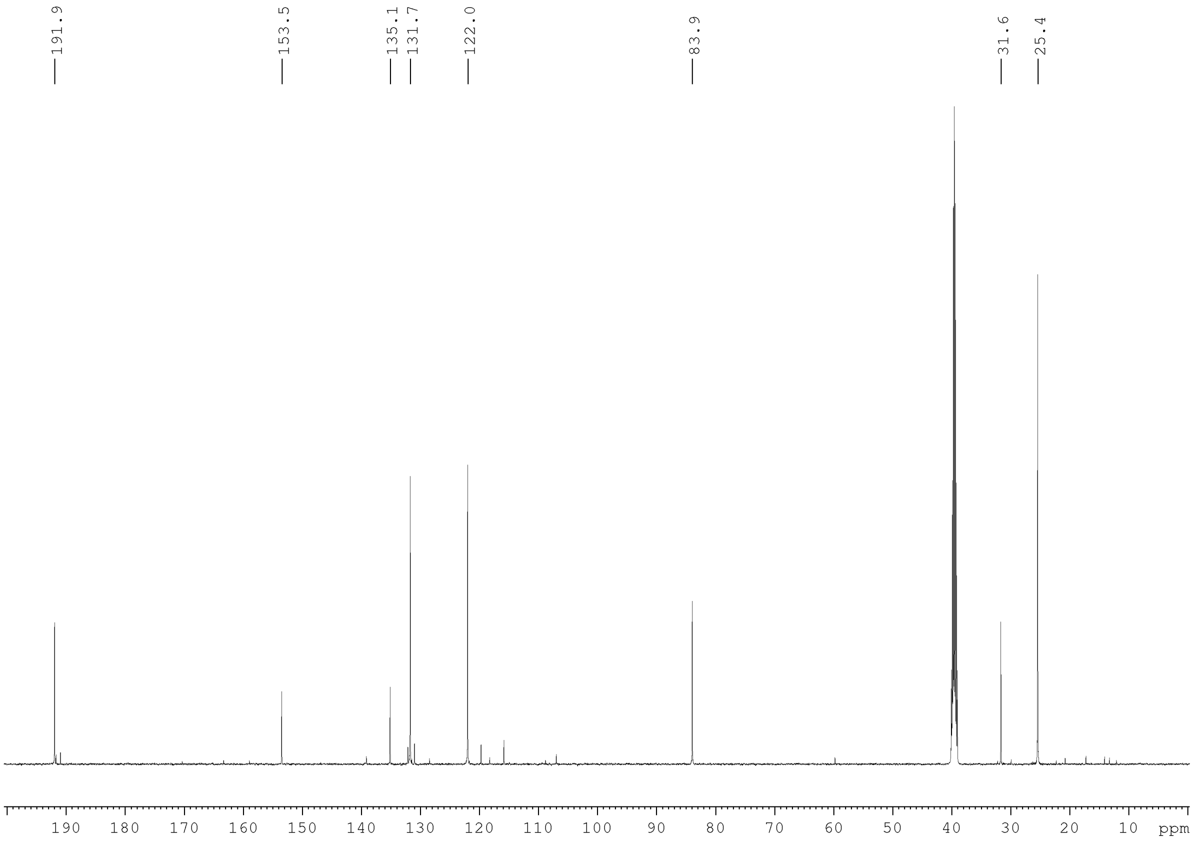

4-(1-hydroxybut-3-yn-1-yl)phenyl neopentyl sulfate (10)

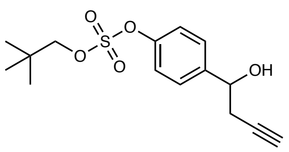

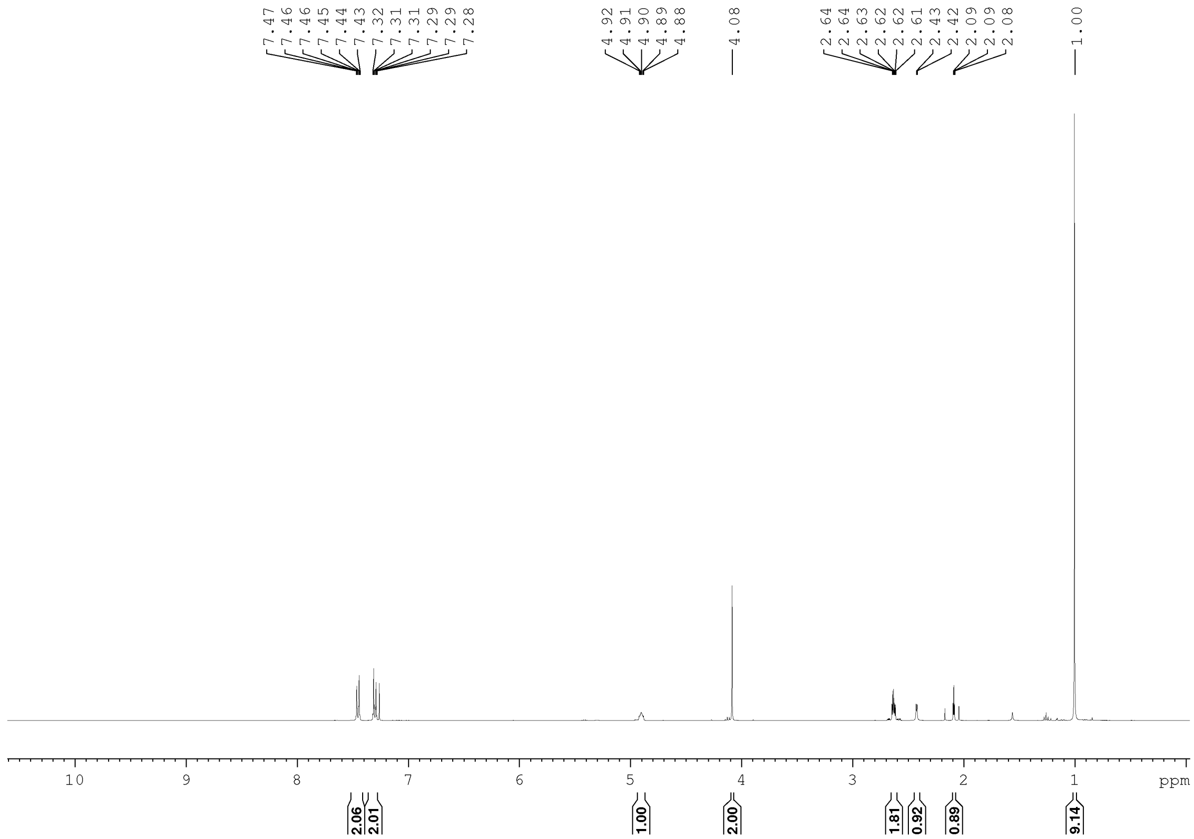

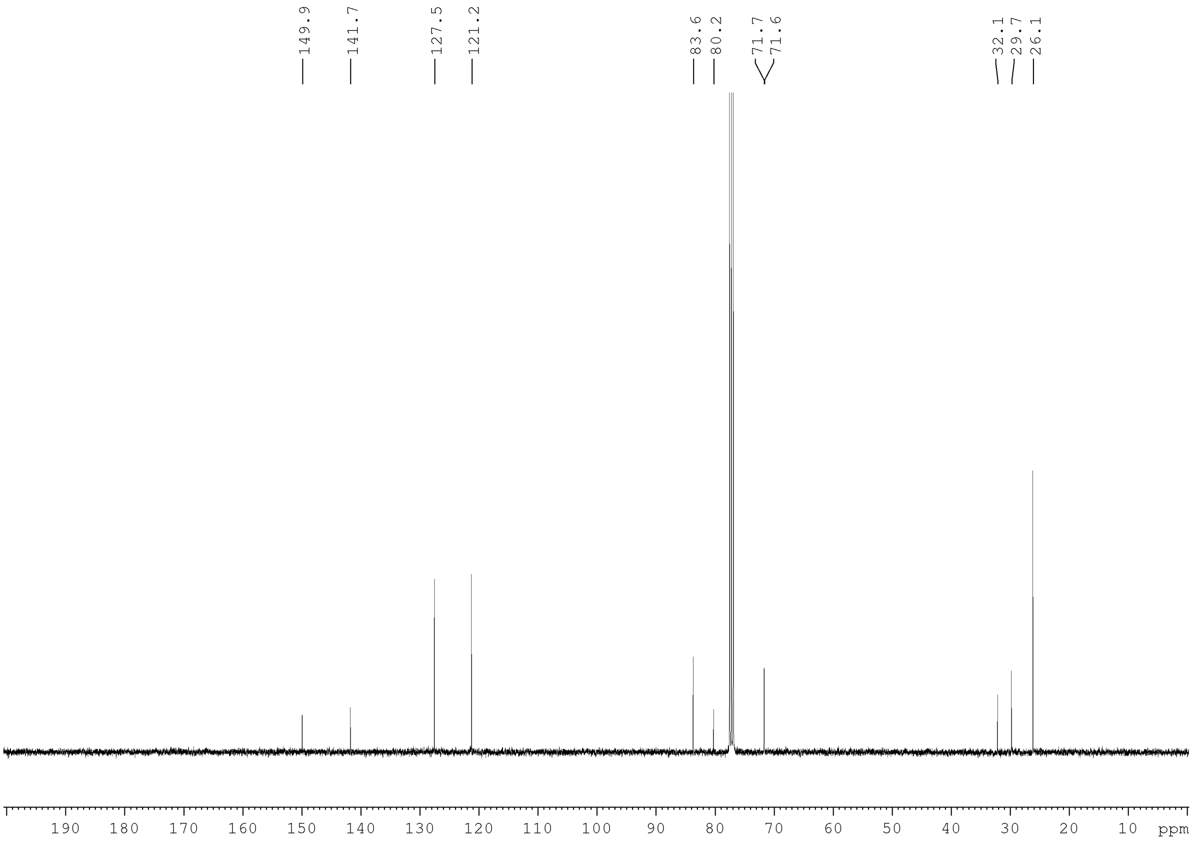

4-(1-(((4-methyl-2-oxo-2*H*-chromen-7-yl)carbamoyl)oxy)but-3-yn-1-yl)phenyl neopentyl sulfate (11)

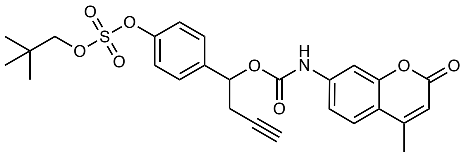

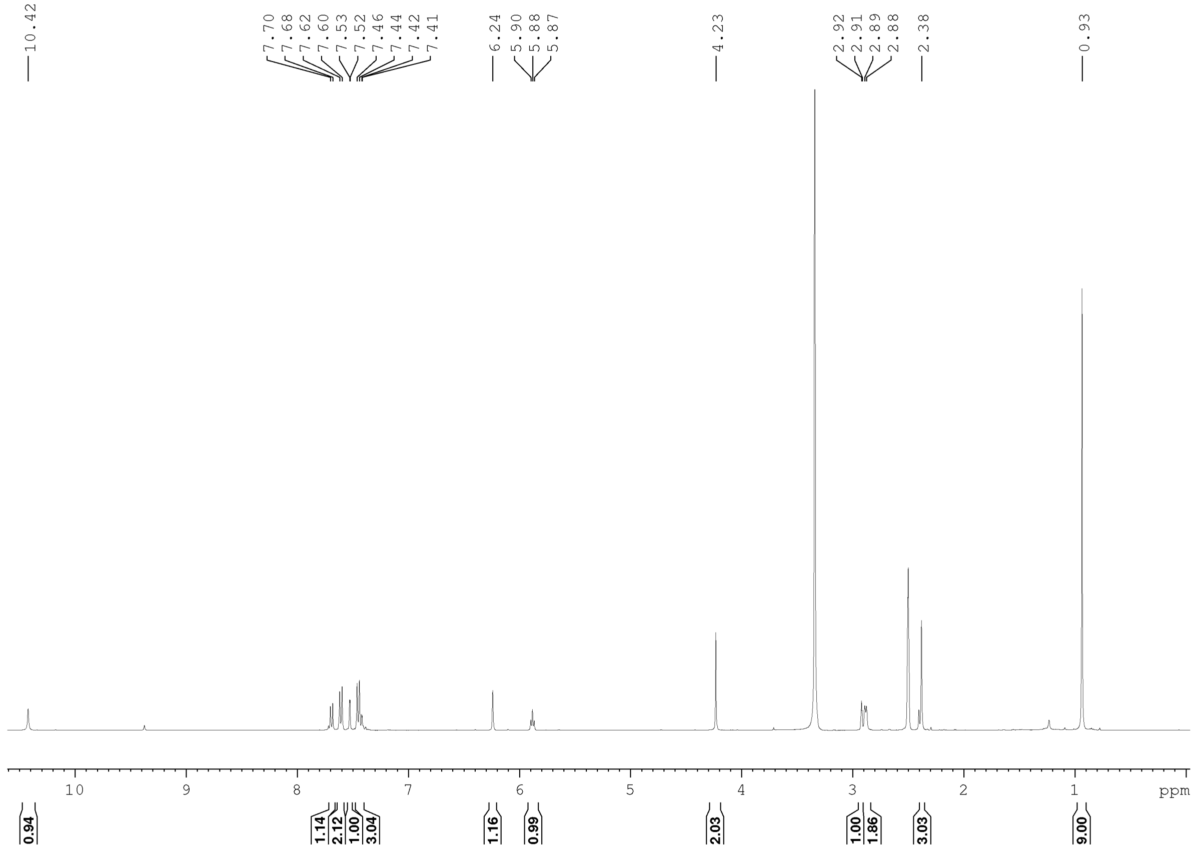

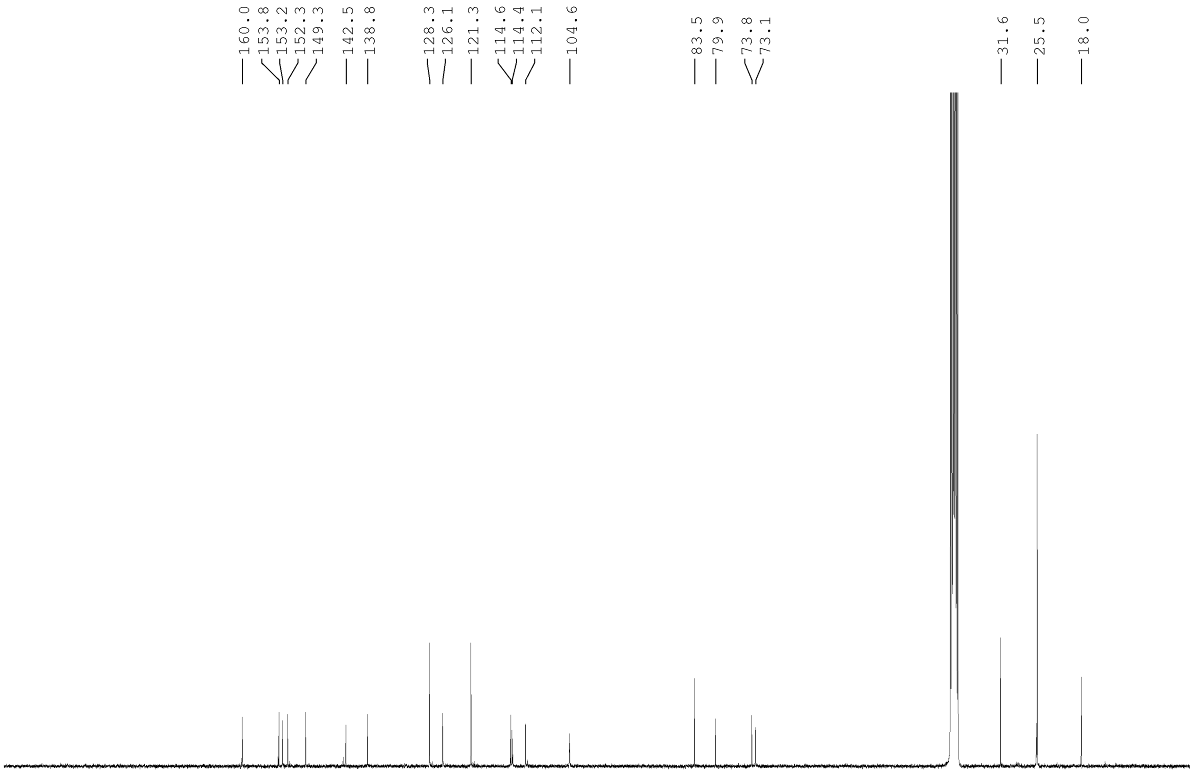

Ammonium 4-(1-(((4-methyl-2-oxo-2*H*-chromen-7-yl)carbamoyl)oxy)but-3-yn-1-yl)phenyl sulfate (12)

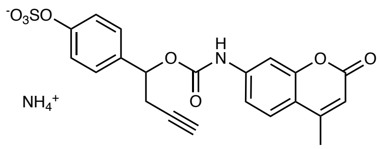

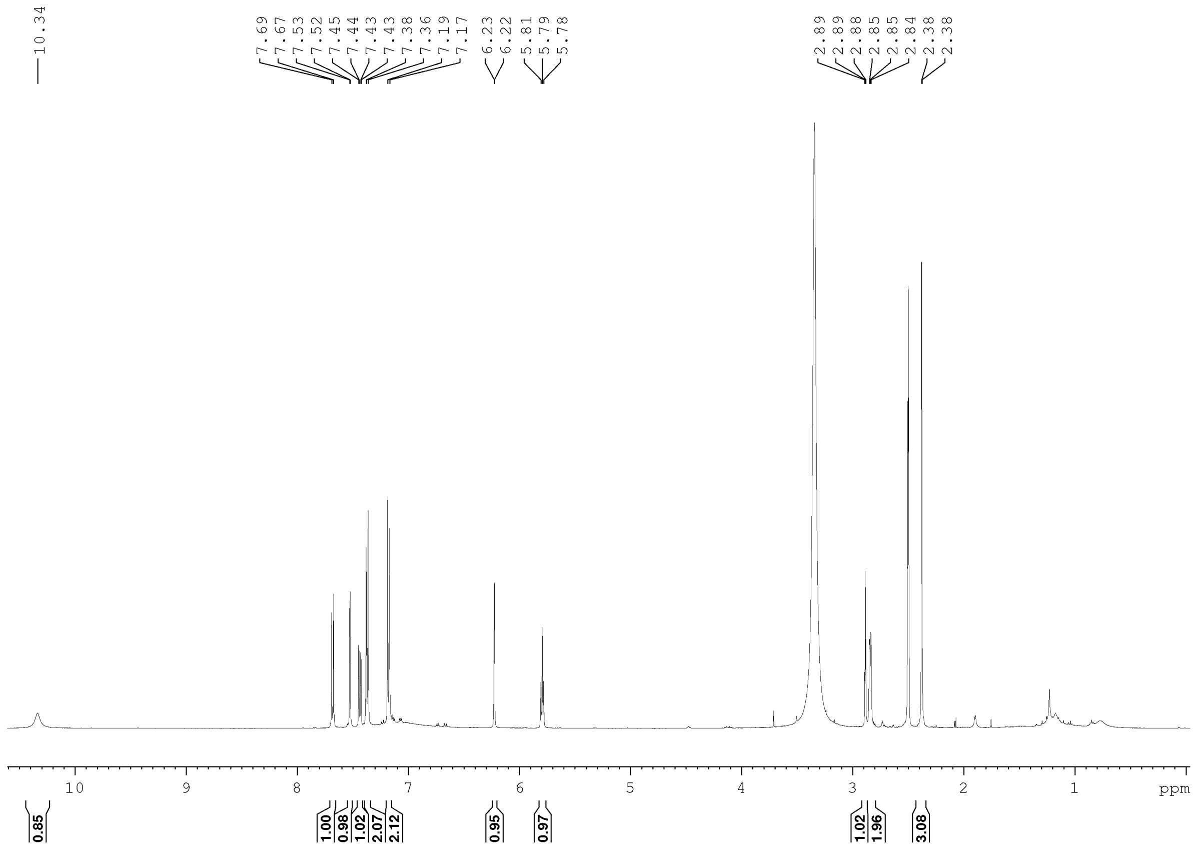

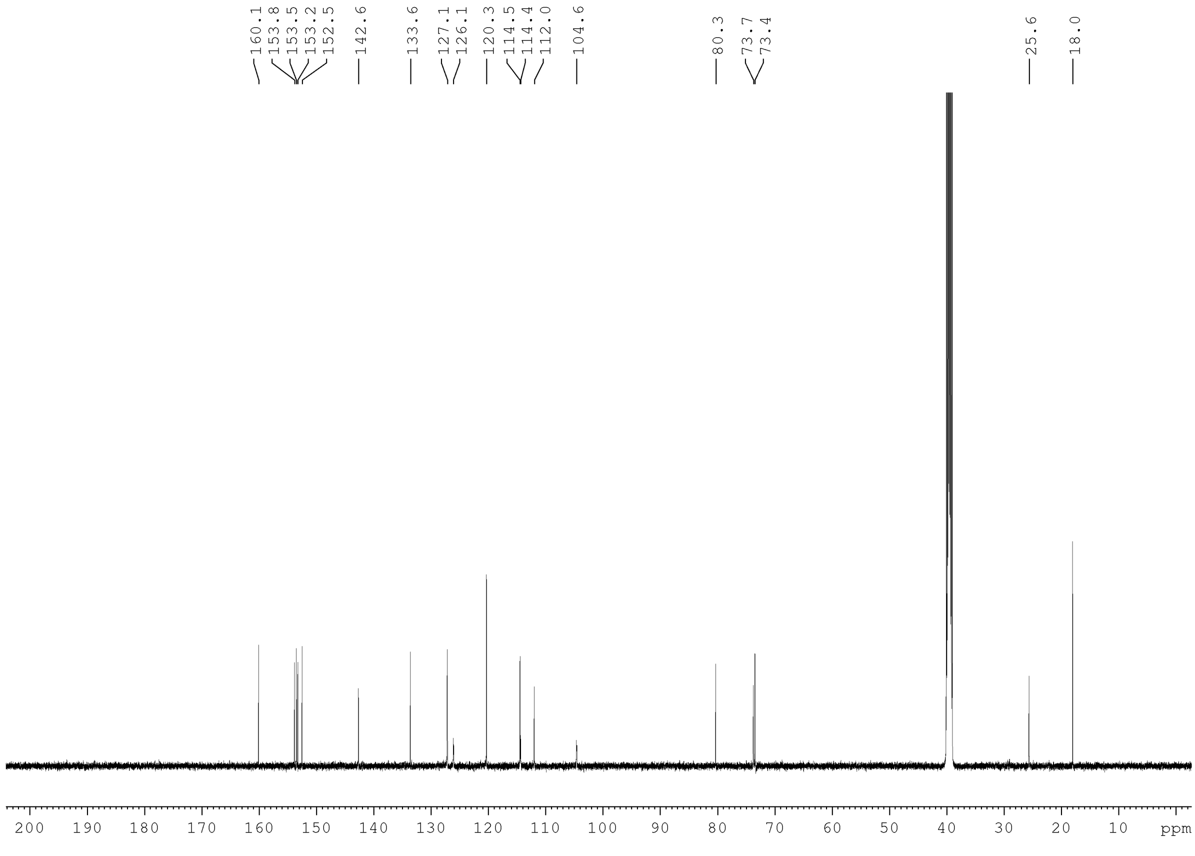

Neopentyl (4-((((4-nitrophenoxy)carbonyl)oxy)methyl)-2-(pent-4-ynamido)phenyl) sulfate (S1)

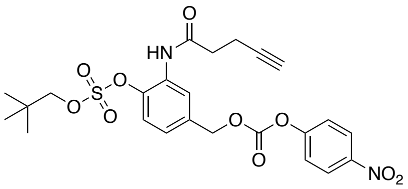

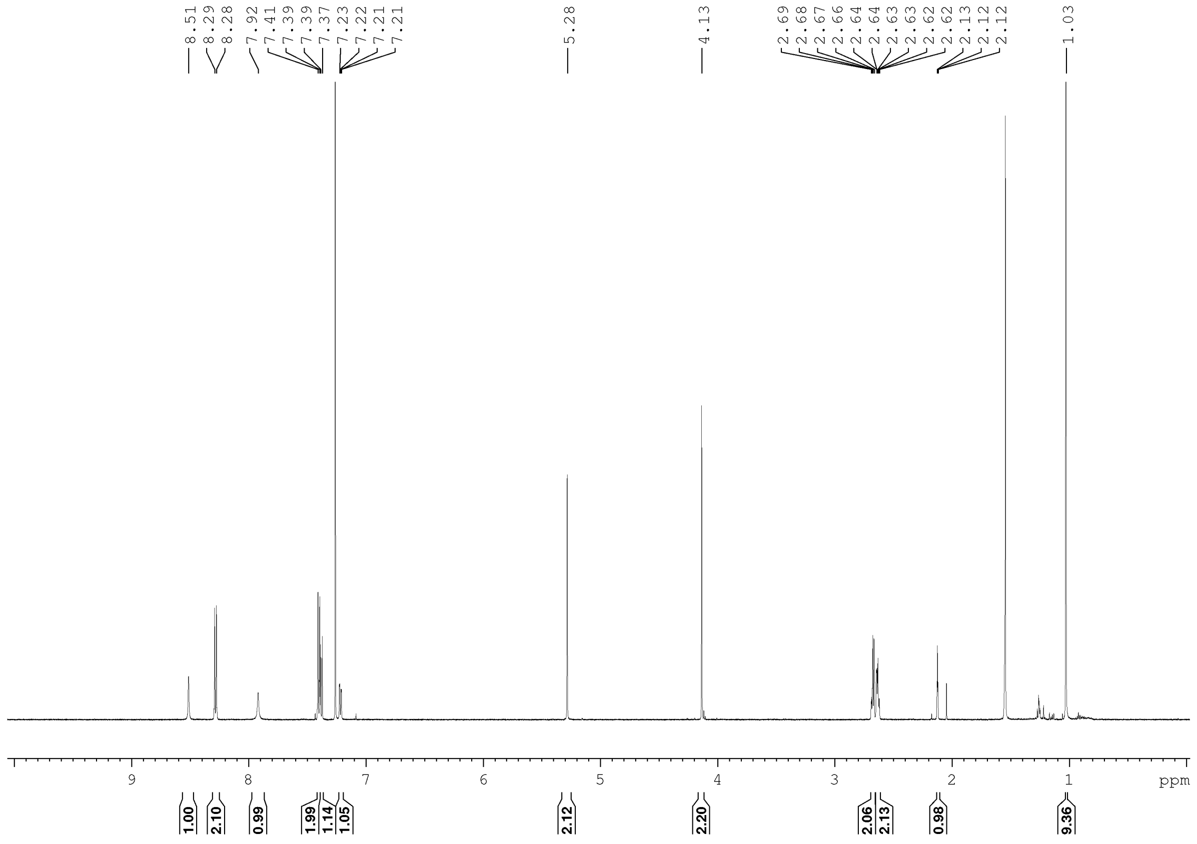

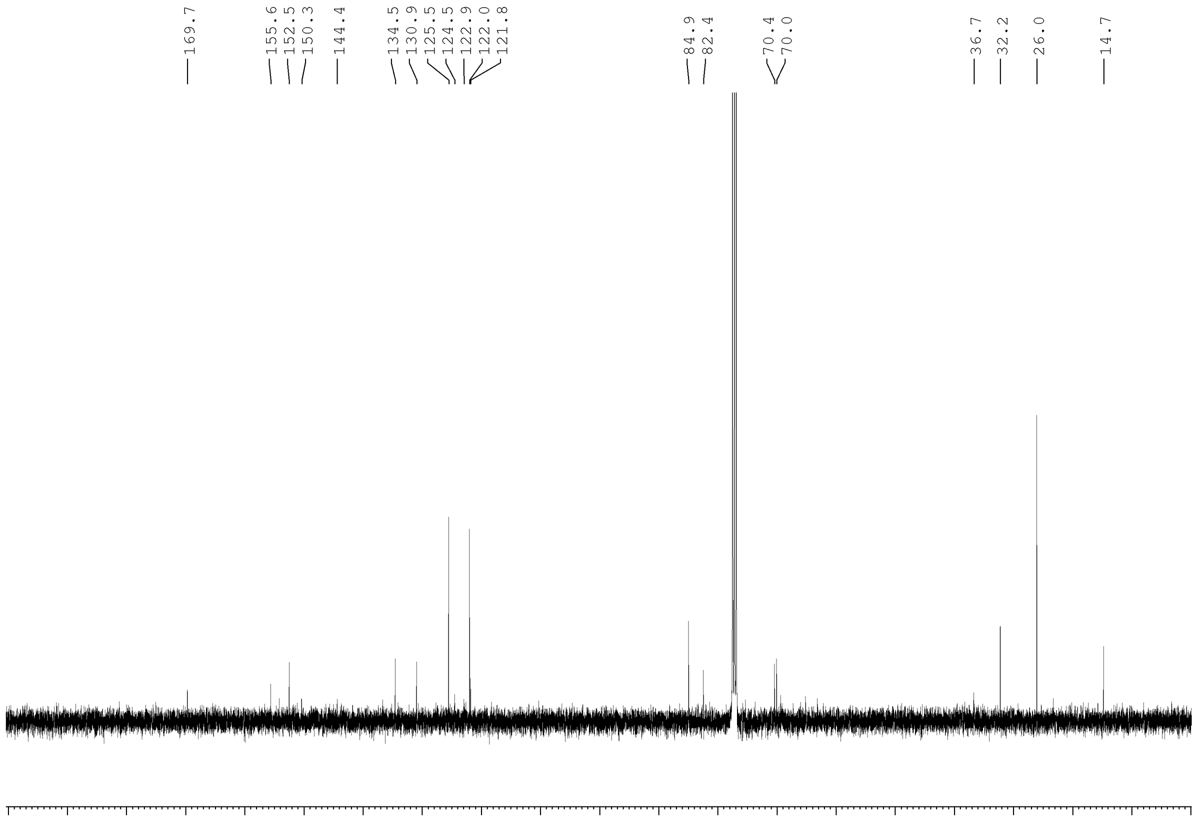

Neopentyl arylsulfate-2-amide-MMAE (S2)

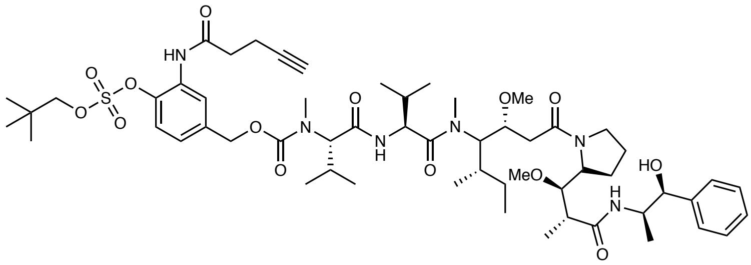

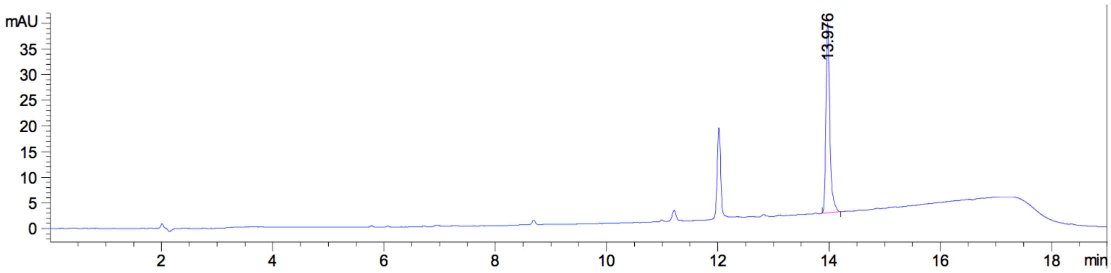

Ammonium arylsulfate-2-amide-MMAE (S3)

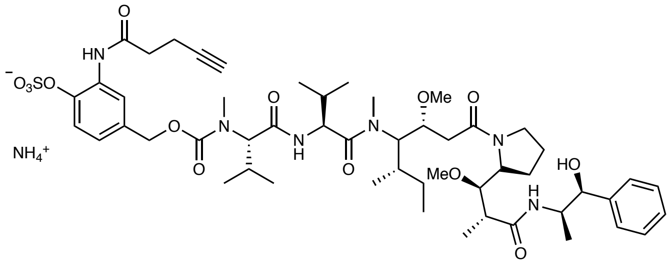

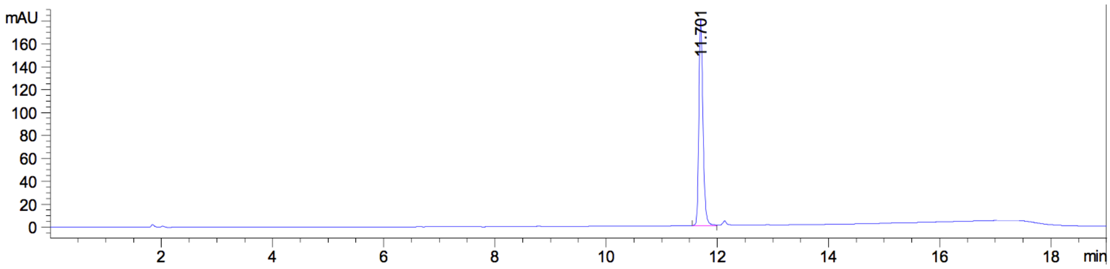

Ammonium DVP-PEG$_3$-arylsulfate-2-amide-MMAE (15)

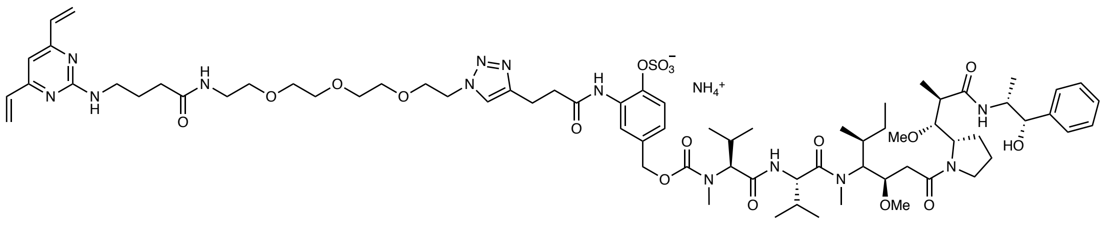

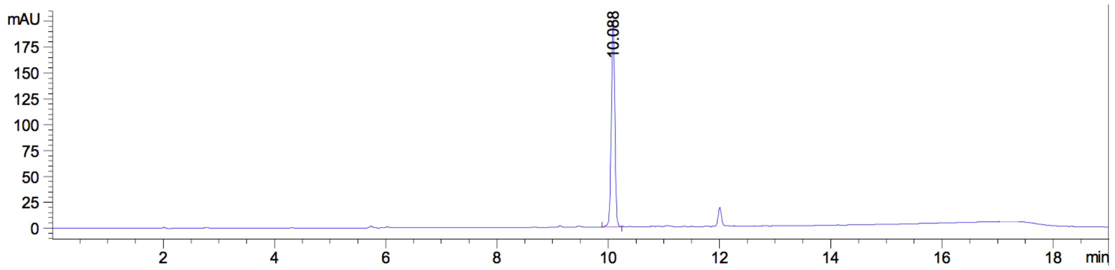

Neopentyl (4-(1-(((4-nitrophenoxy)carbonyl)oxy)but-3-yn-1-yl)phenyl) sulfate (S4)

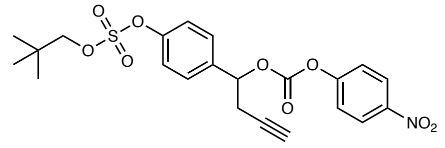

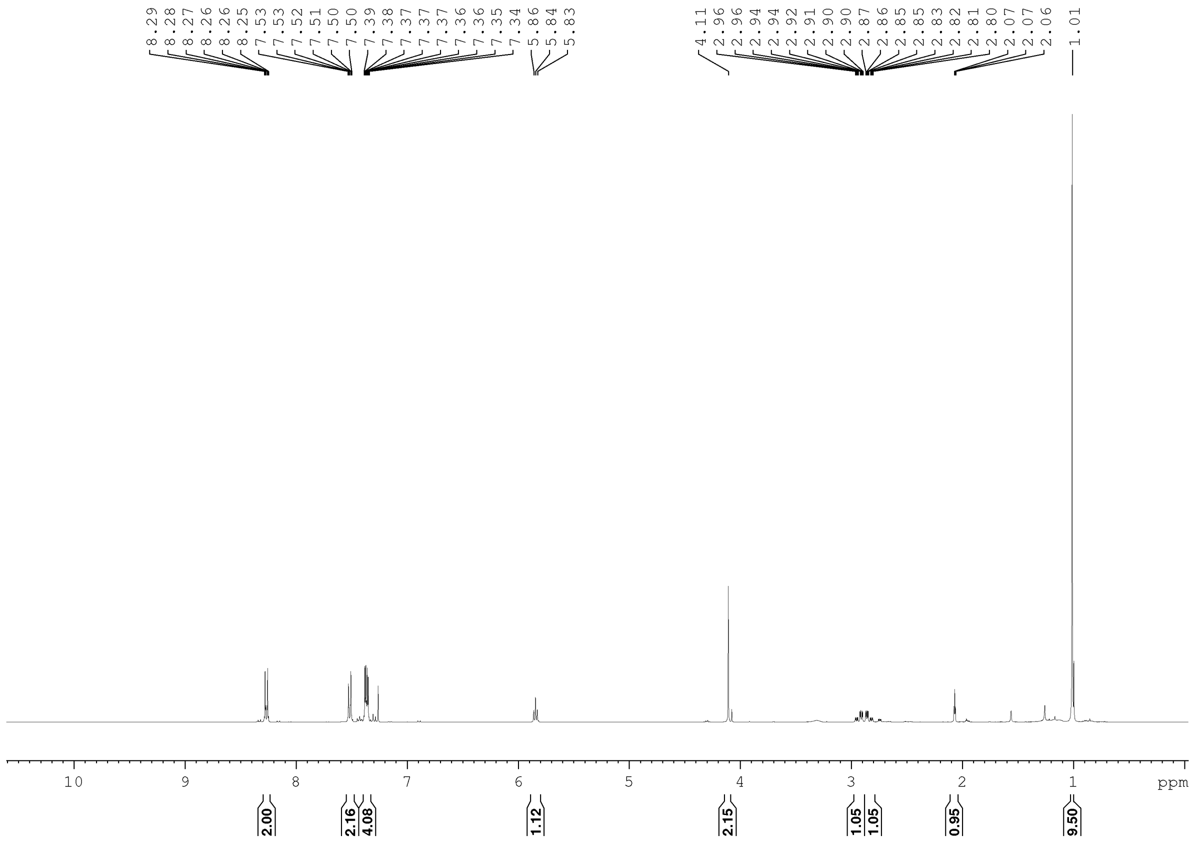

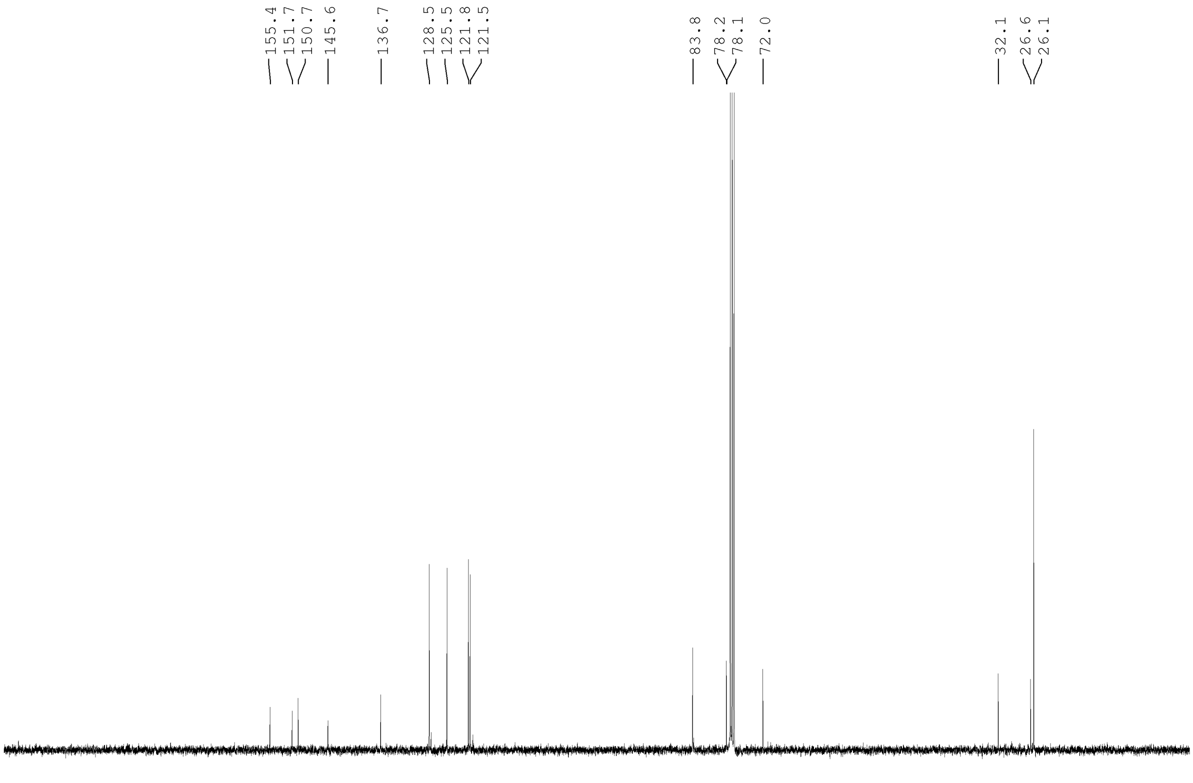

Neopentyl arylsulfate-4-alkyl-MMAE (S5)

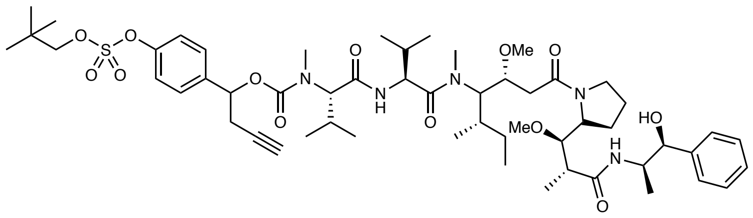

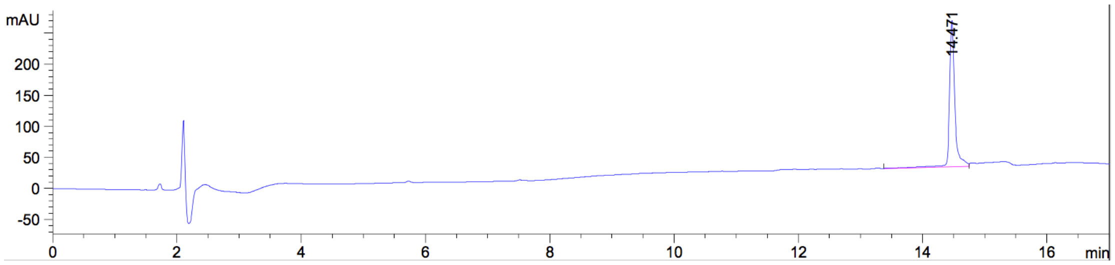

Ammonium arylsulfate-4-alkyl-MMAE (S6)

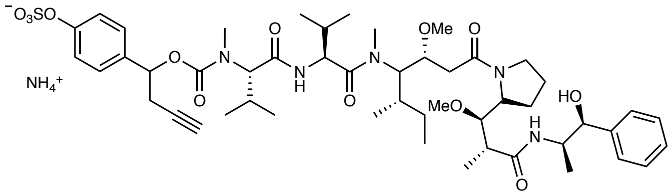

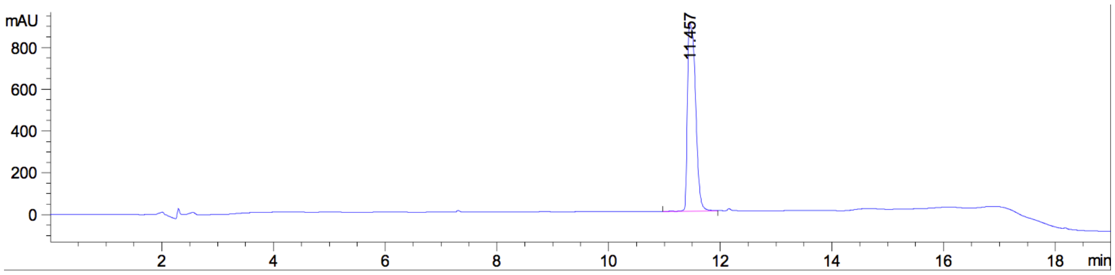

Ammonium DVP-PEG$_3$-arylsulfate-4-alkyl-MMAE (16a)

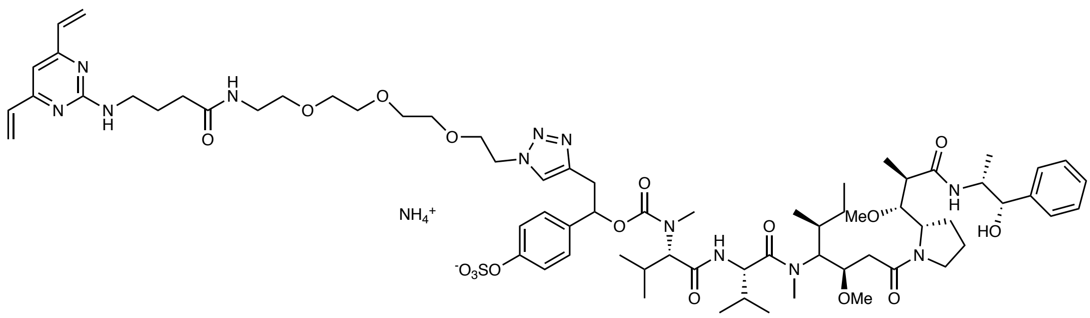

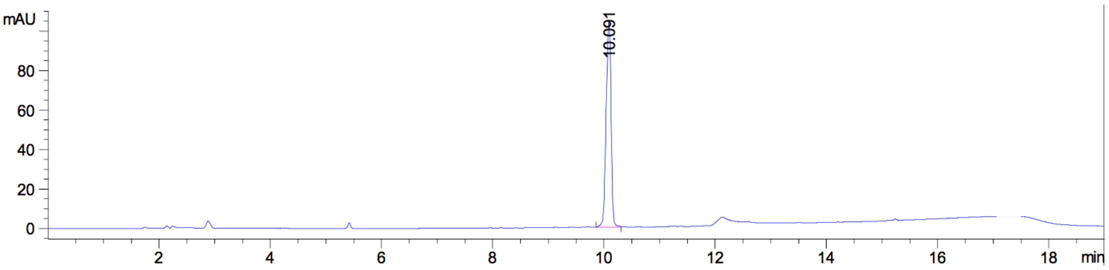

4-(1-hydroxybut-3-yn-1-yl)-2-nitrophenol (S7)

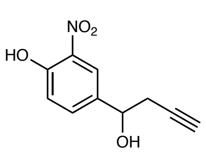

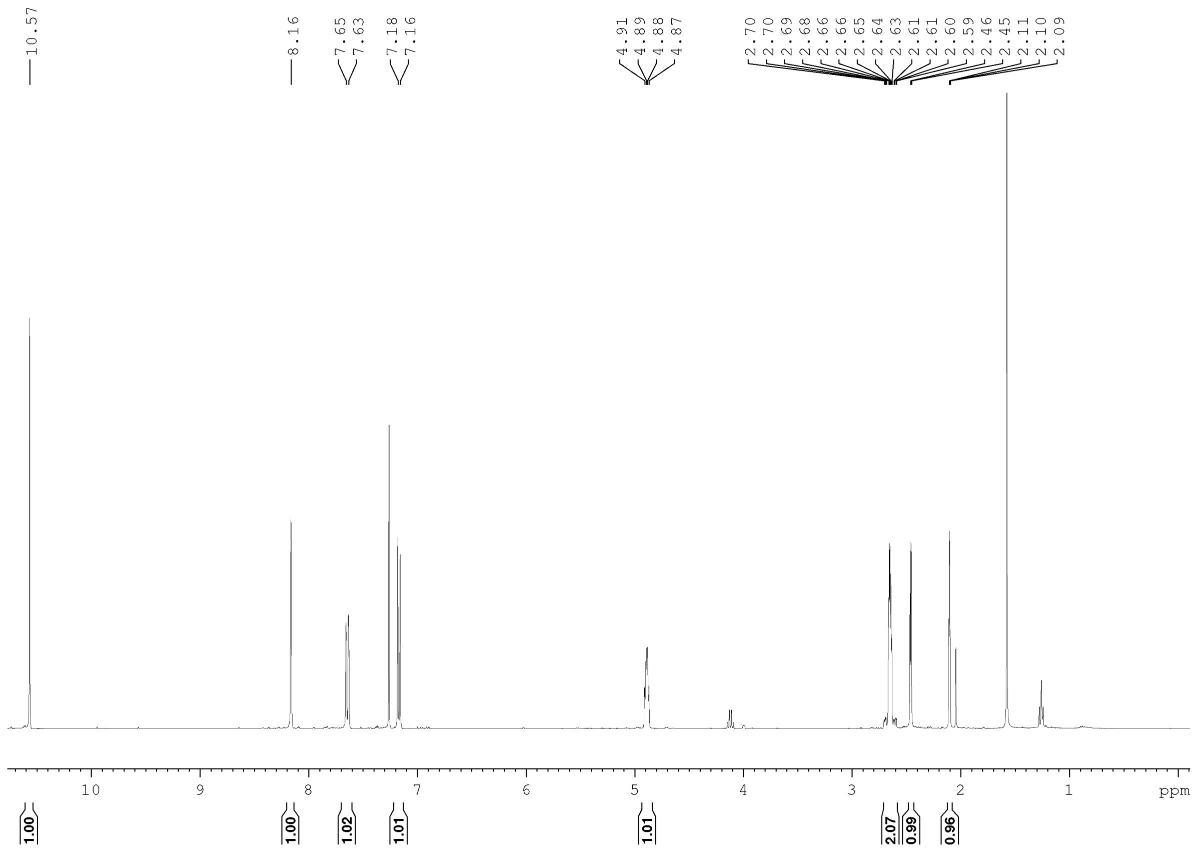

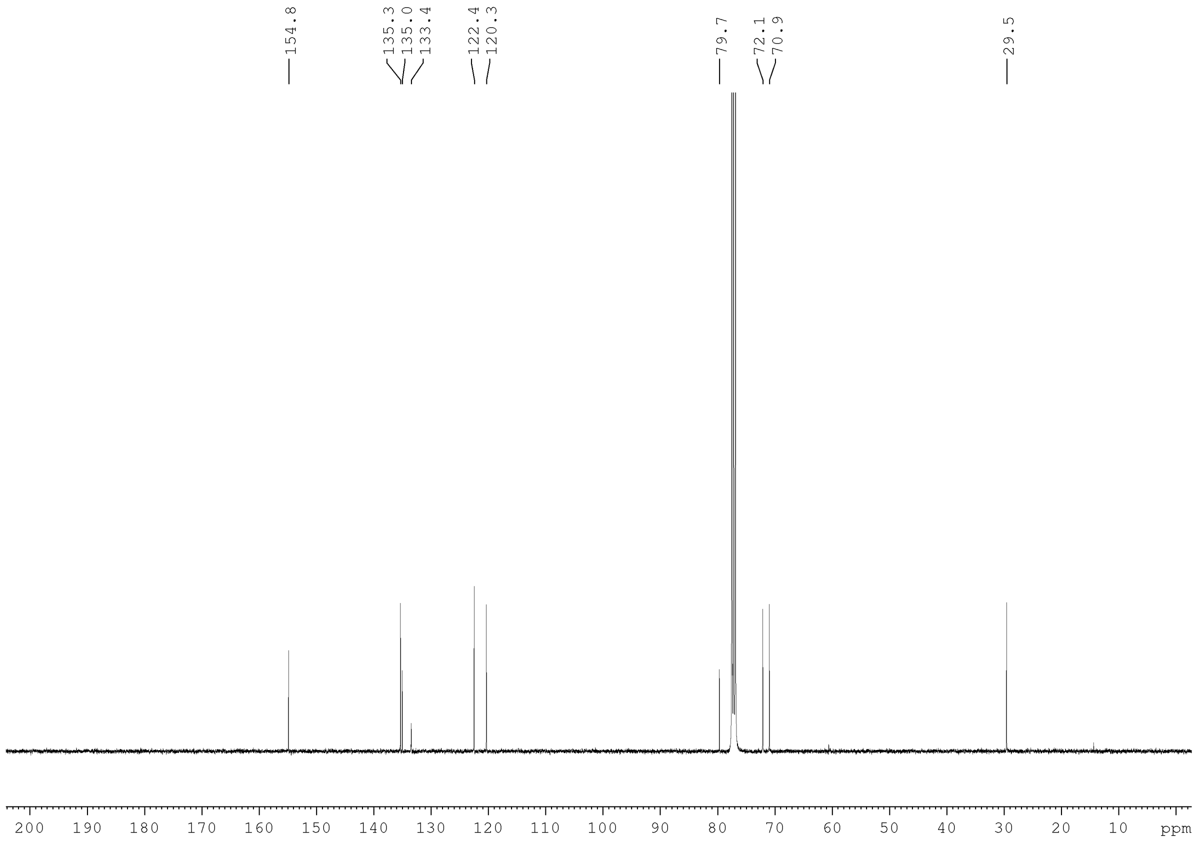

4-(1-hydroxybut-3-yn-1-yl)-2-nitrophenyl neopentyl sulfate (S8)

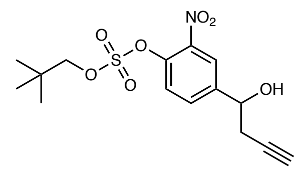

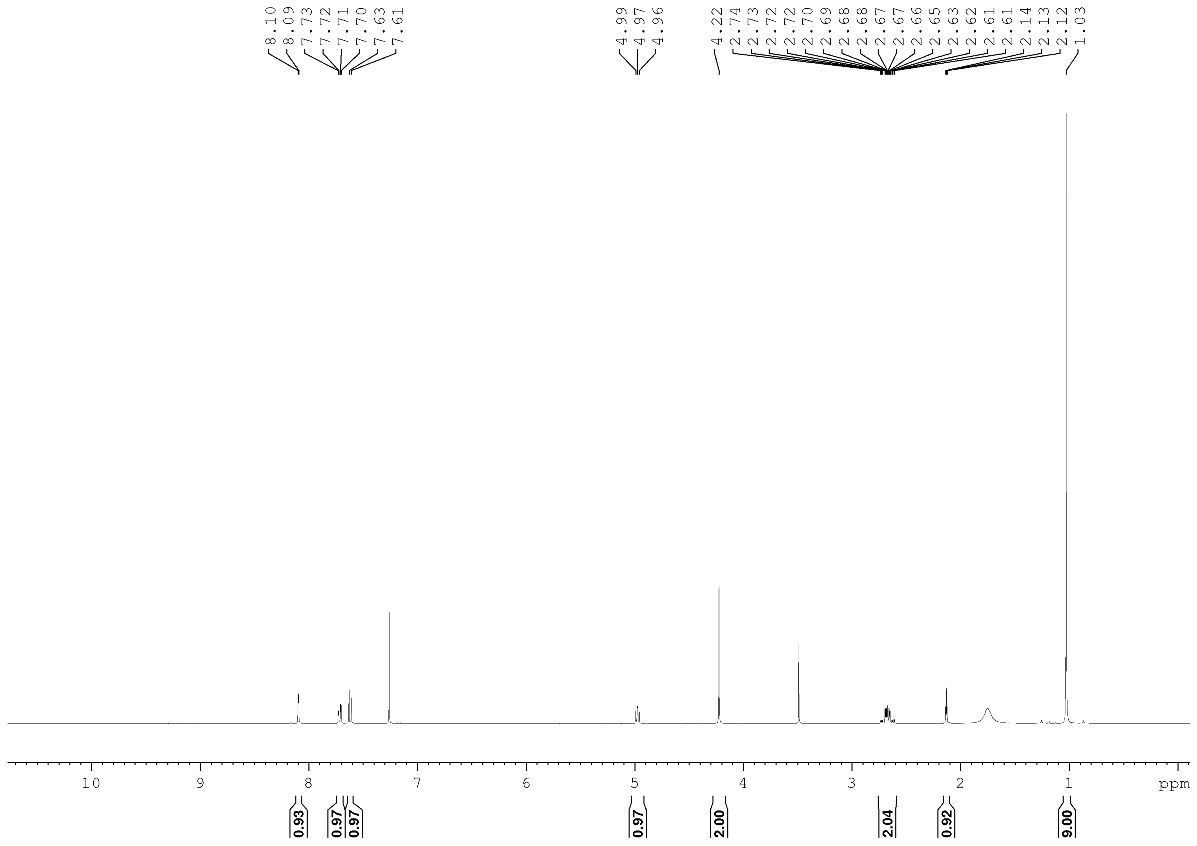

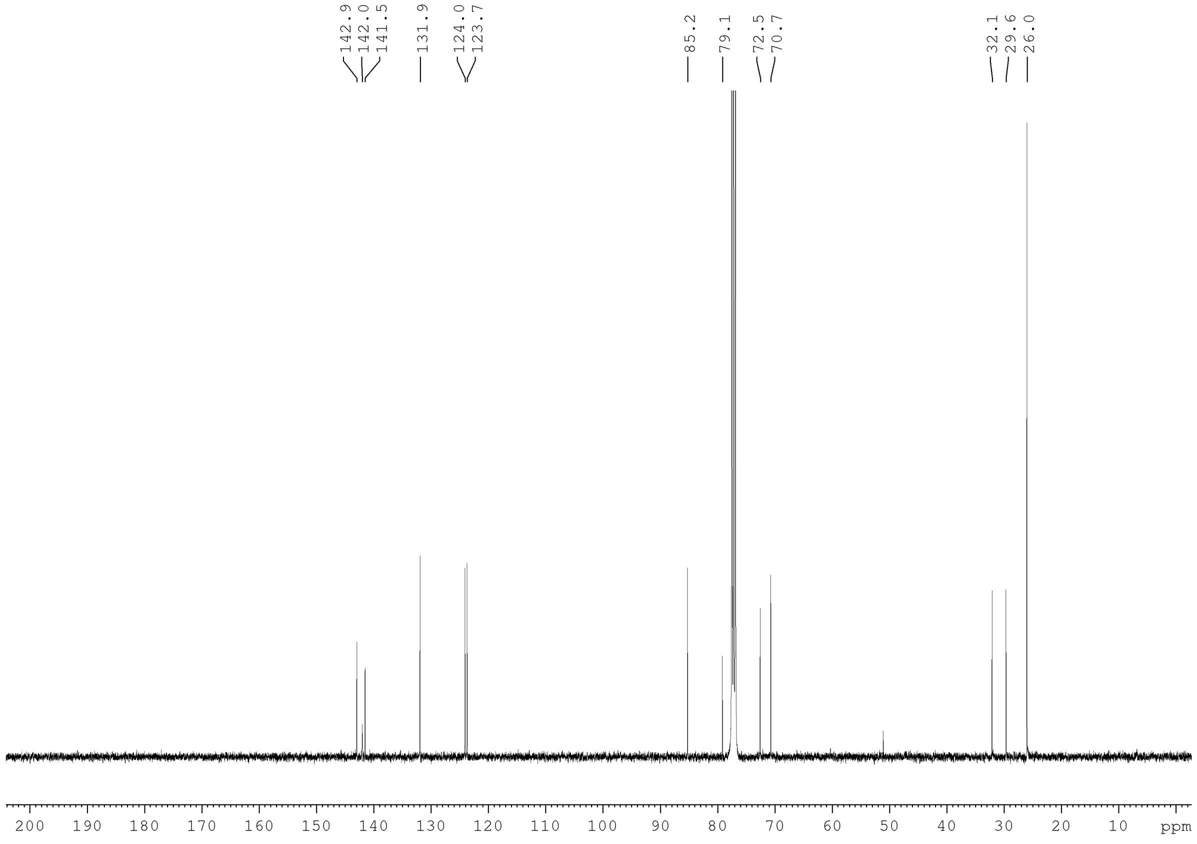

Neopentyl (2-nitro-4-(1-(((4-nitrophenoxy)carbonyl)oxy)but-3-yn-1-yl)phenyl) sulfate (S9)

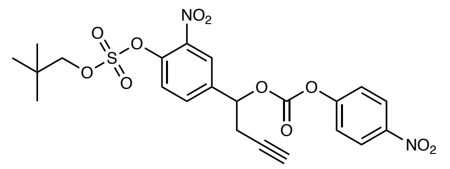

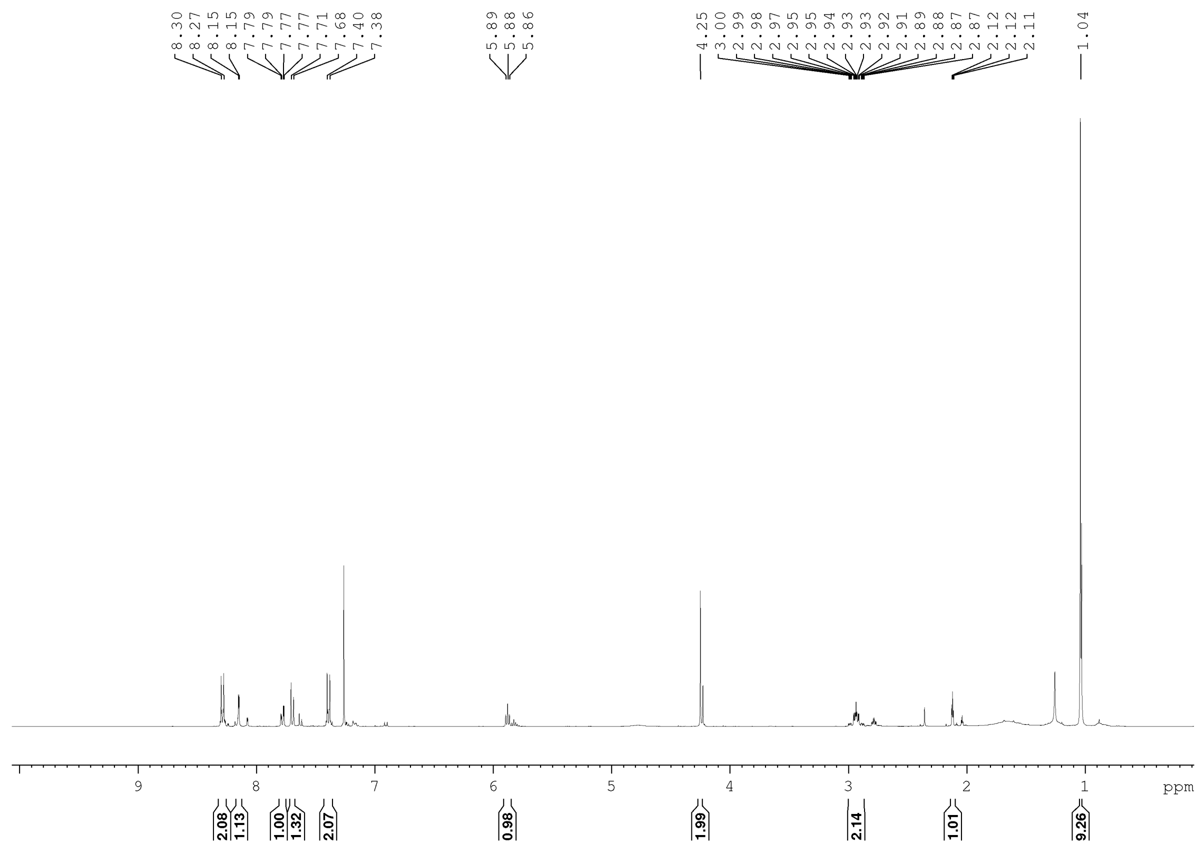

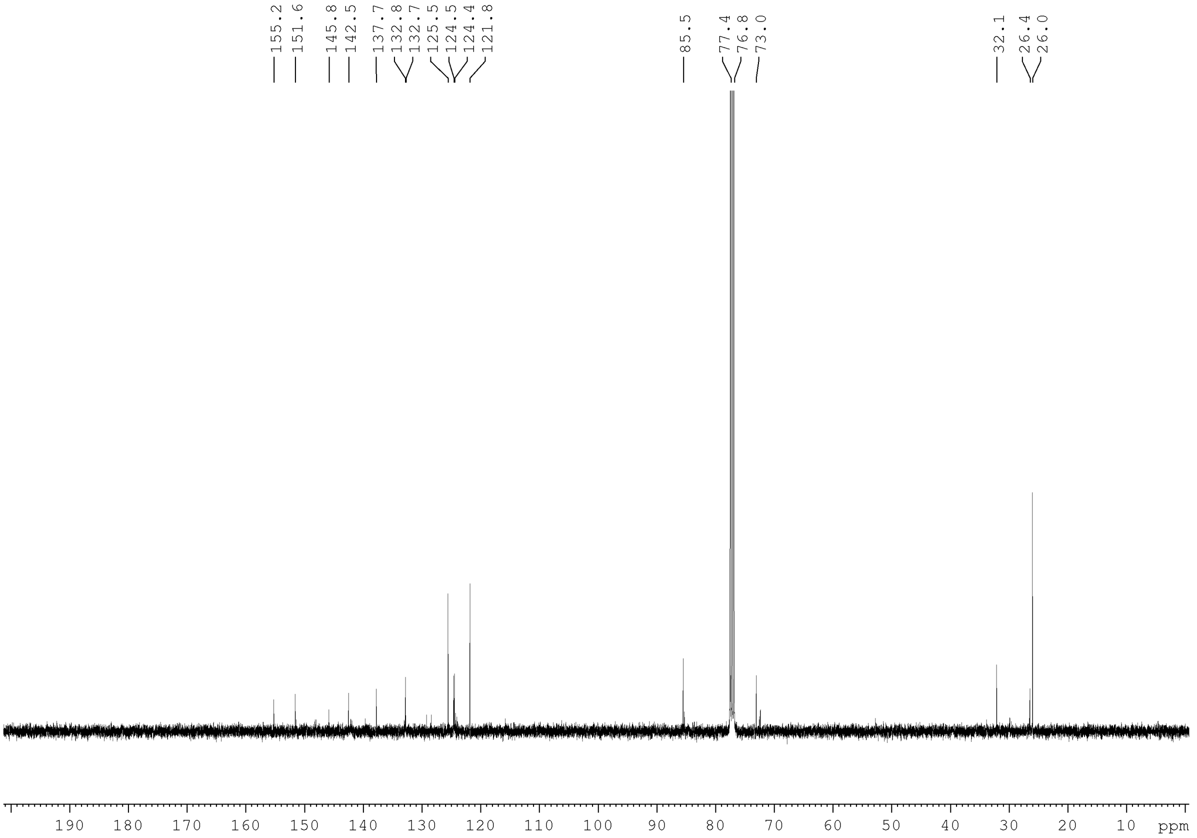

Neopentyl-nitroarylsulfate-MMAE (S10)

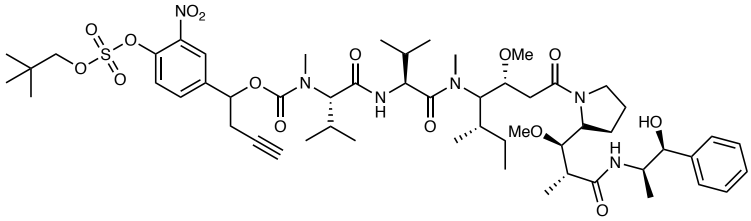

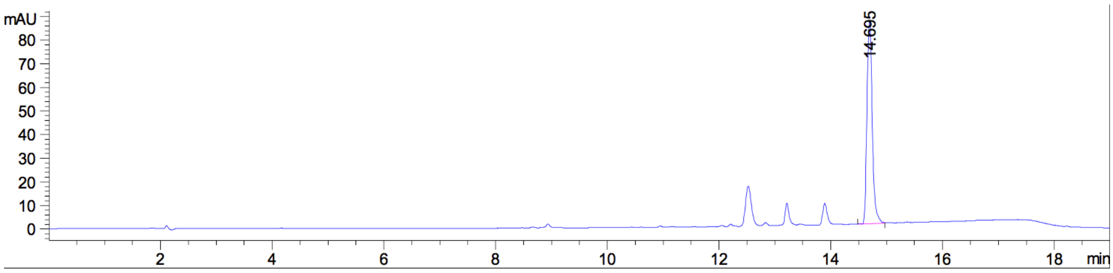

Ammonium nitroarylsulfate-MMAE (S11)

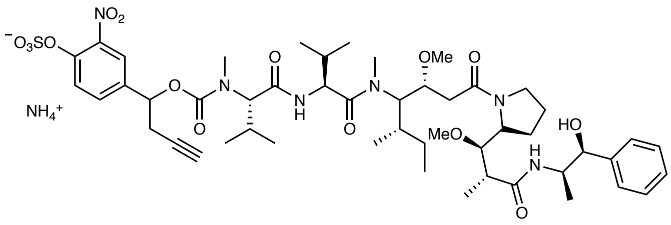

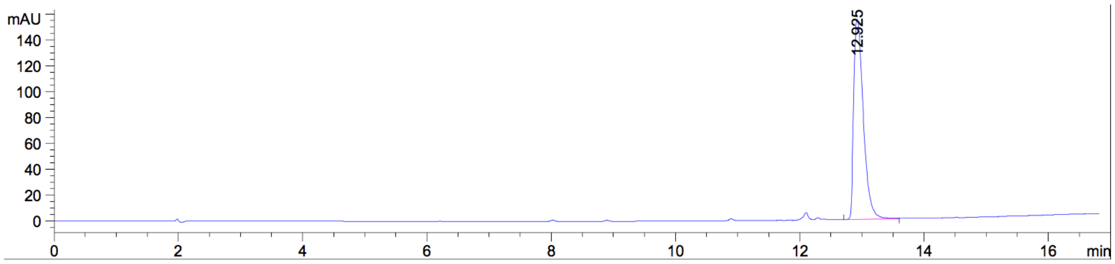

Ammonium DVP-PEG$_3$-nitroarylsulfate-MMAE (16b)

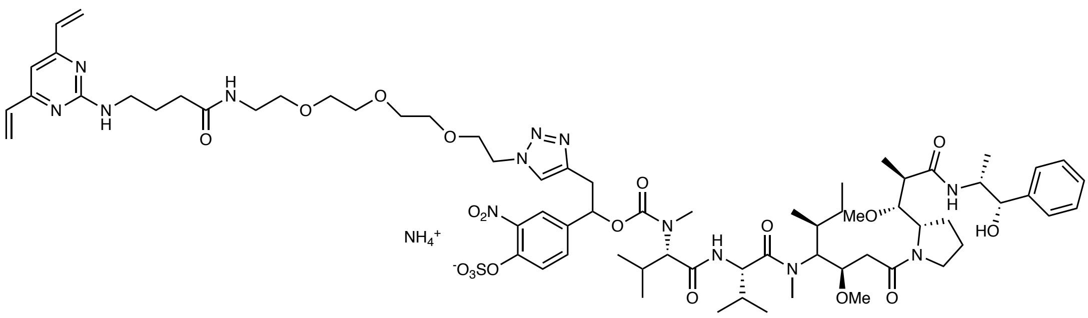

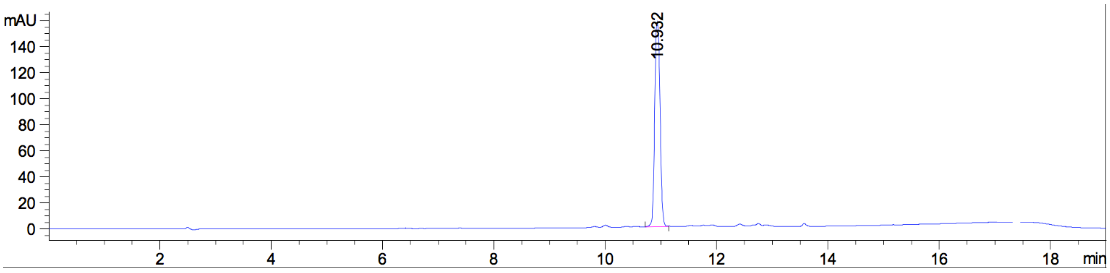

Alkyne-MMAE (S12)

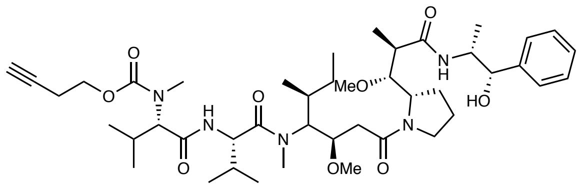

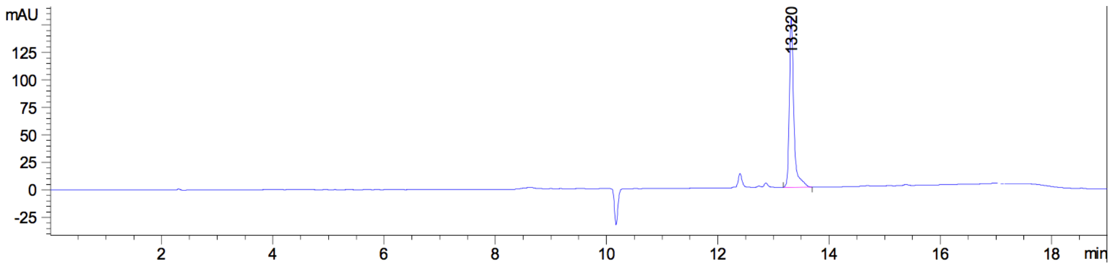

DVP-PEG$_3$-MMAE (17)

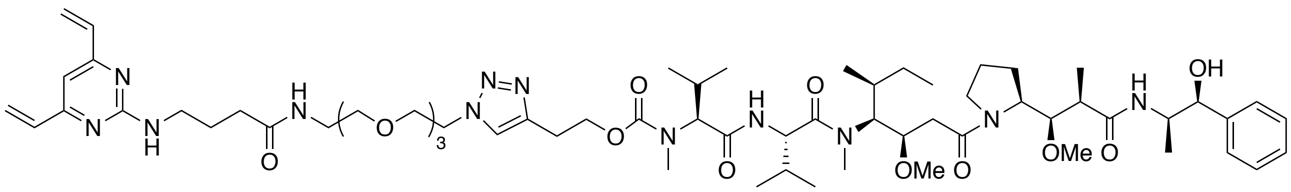

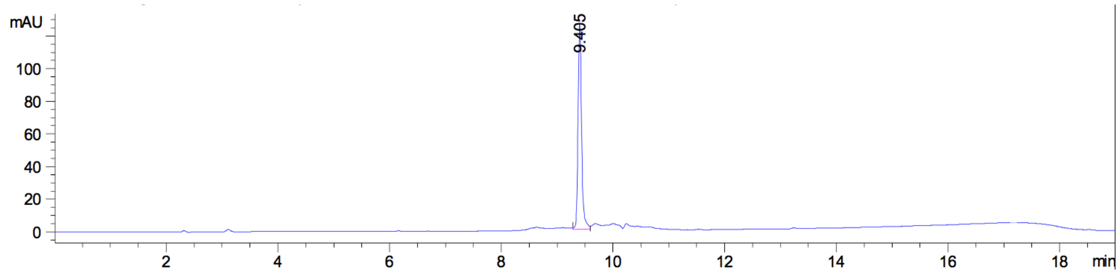

Ethyl 4-((4,6-dichloropyrimidin-2-yl)amino)butanoate (S13)

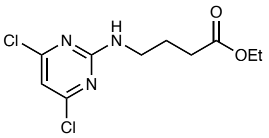

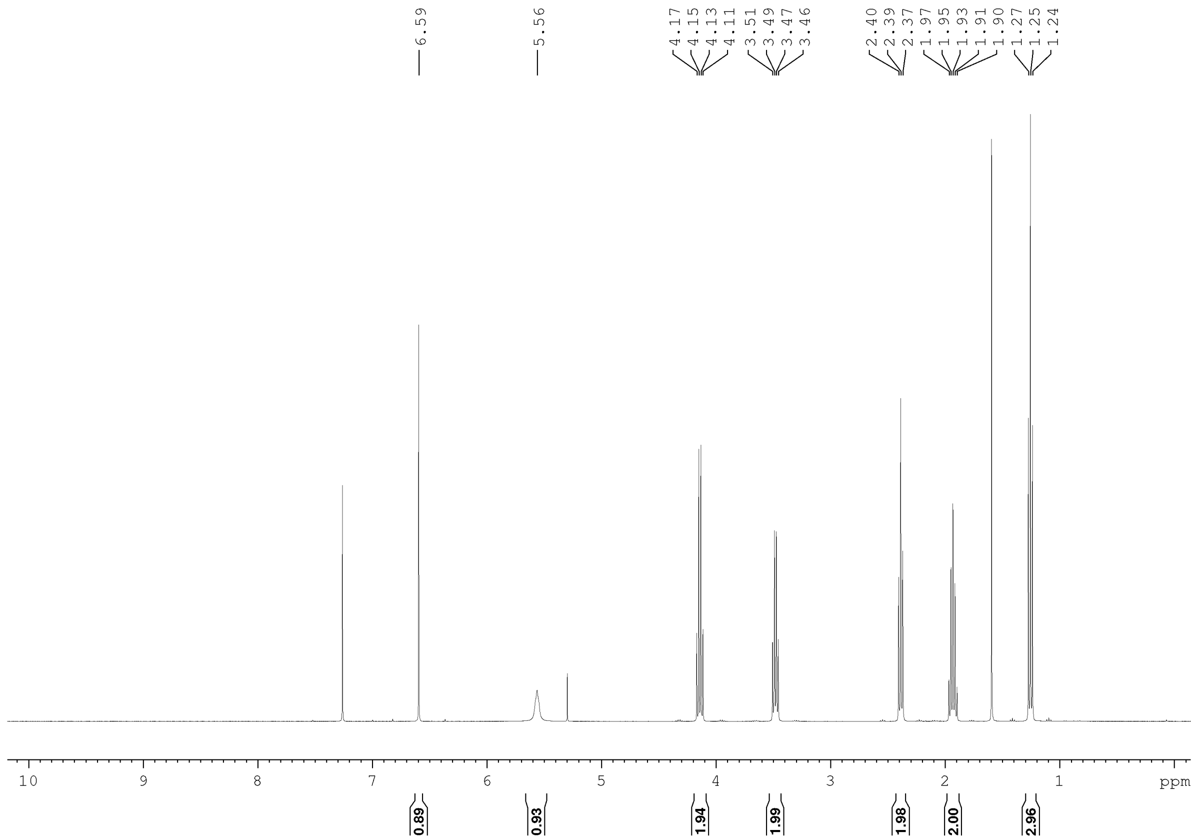

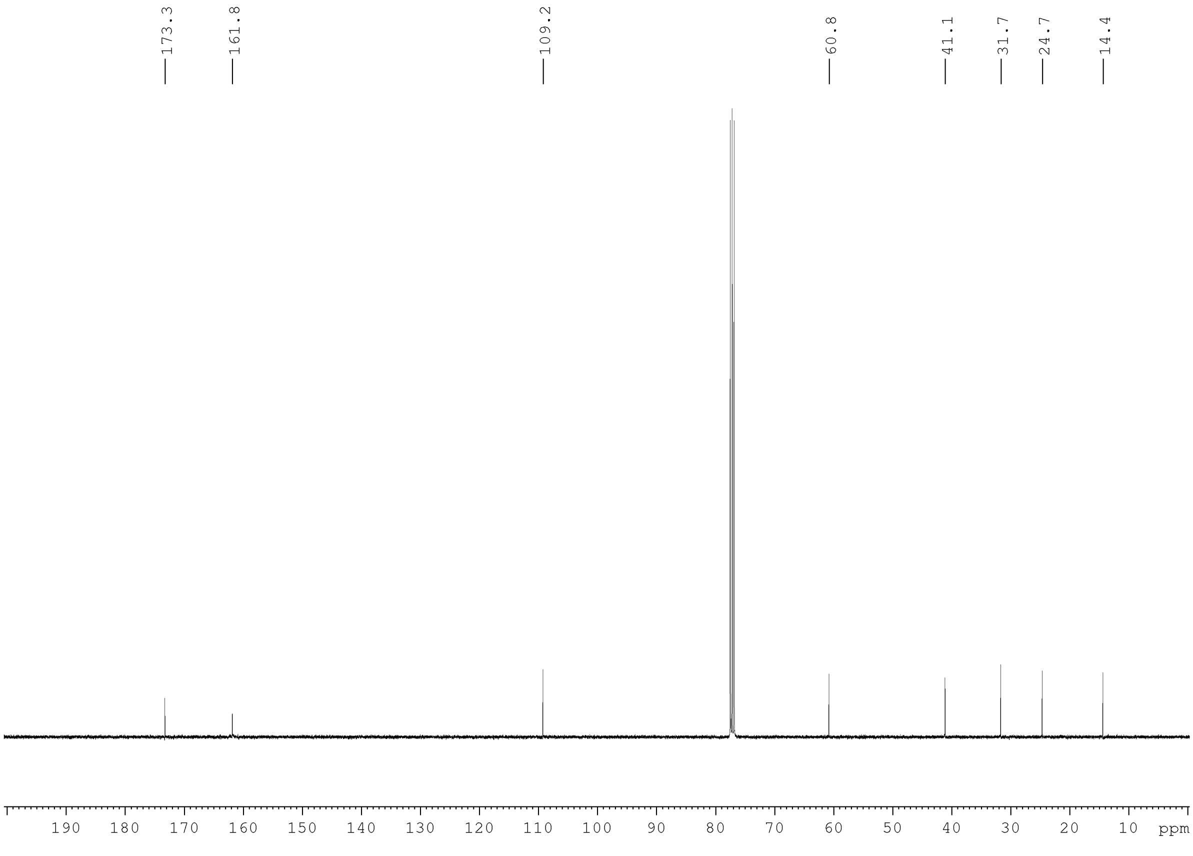

Ethyl 4-((4,6-divinylpyrimidin-2-yl)amino)butanoate (S14)

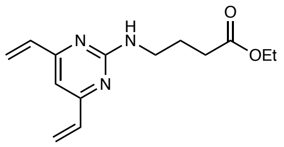

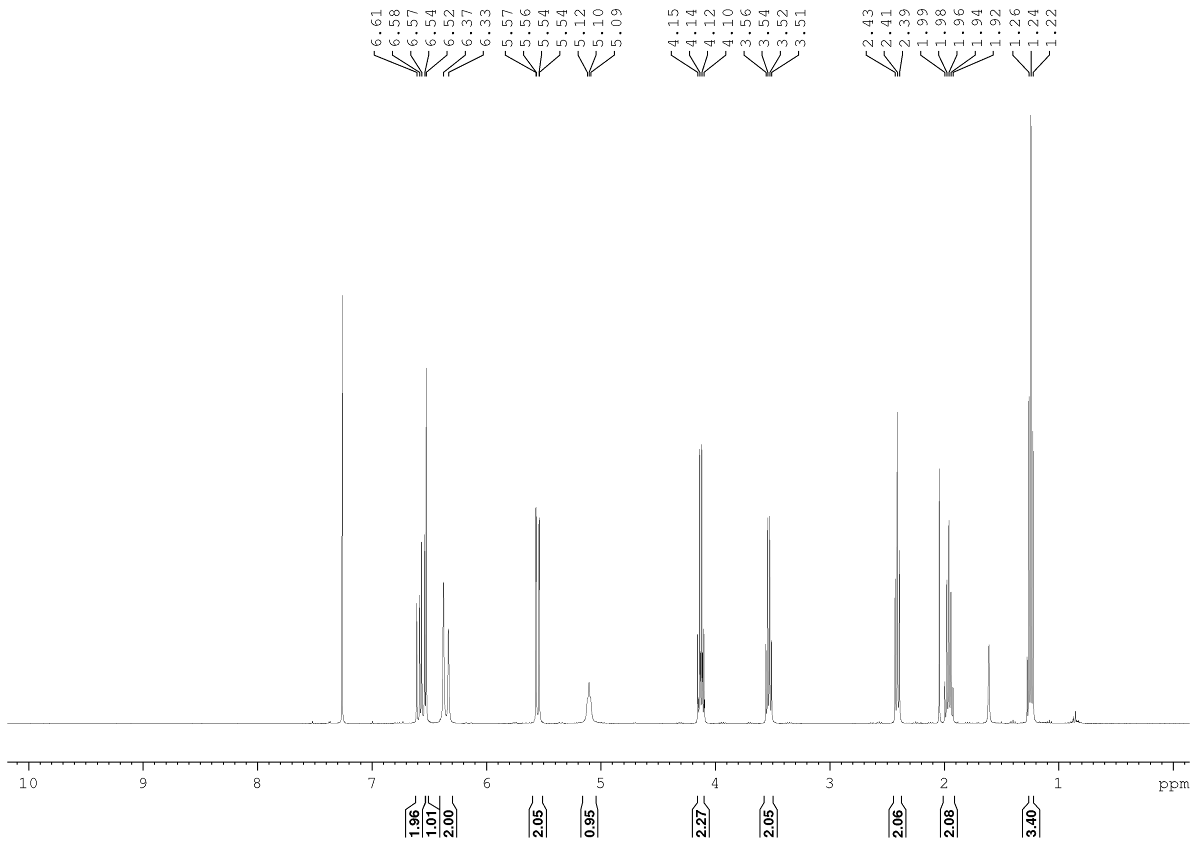

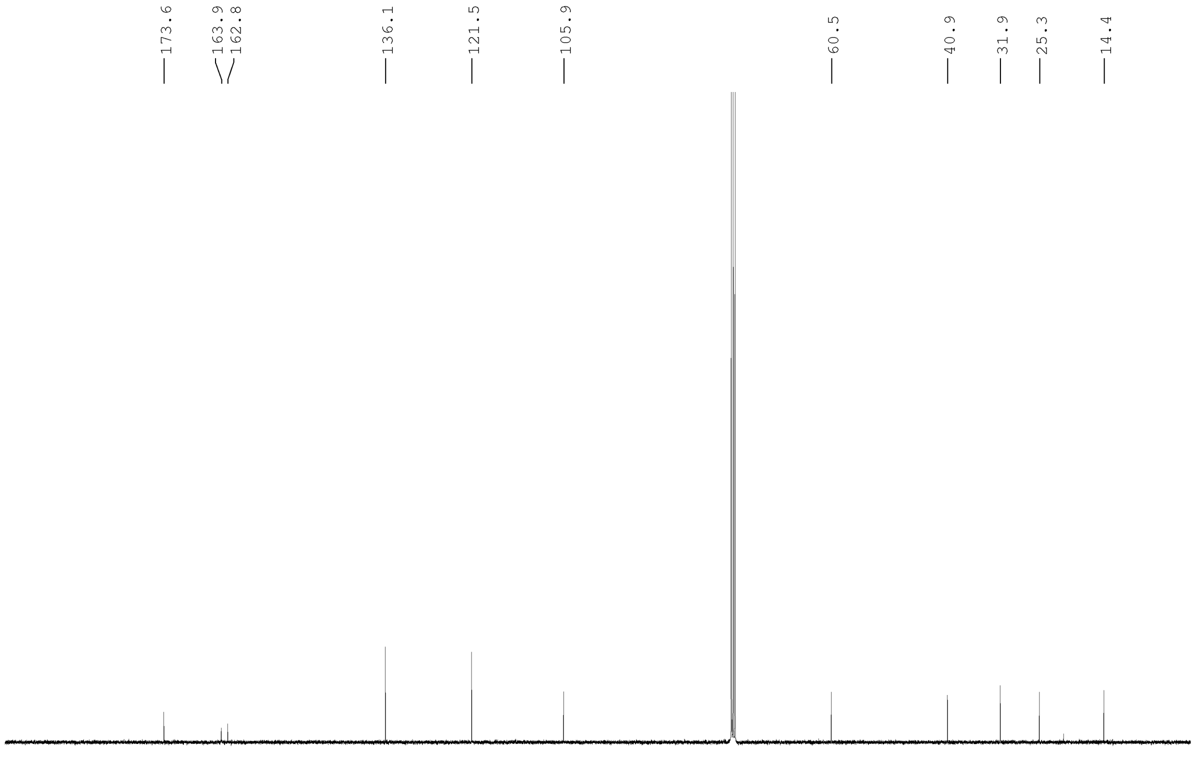

4-((4,6-divinylpyrimidin-2-yl)amino)butanoic acid (S15)

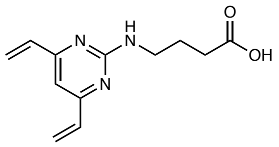

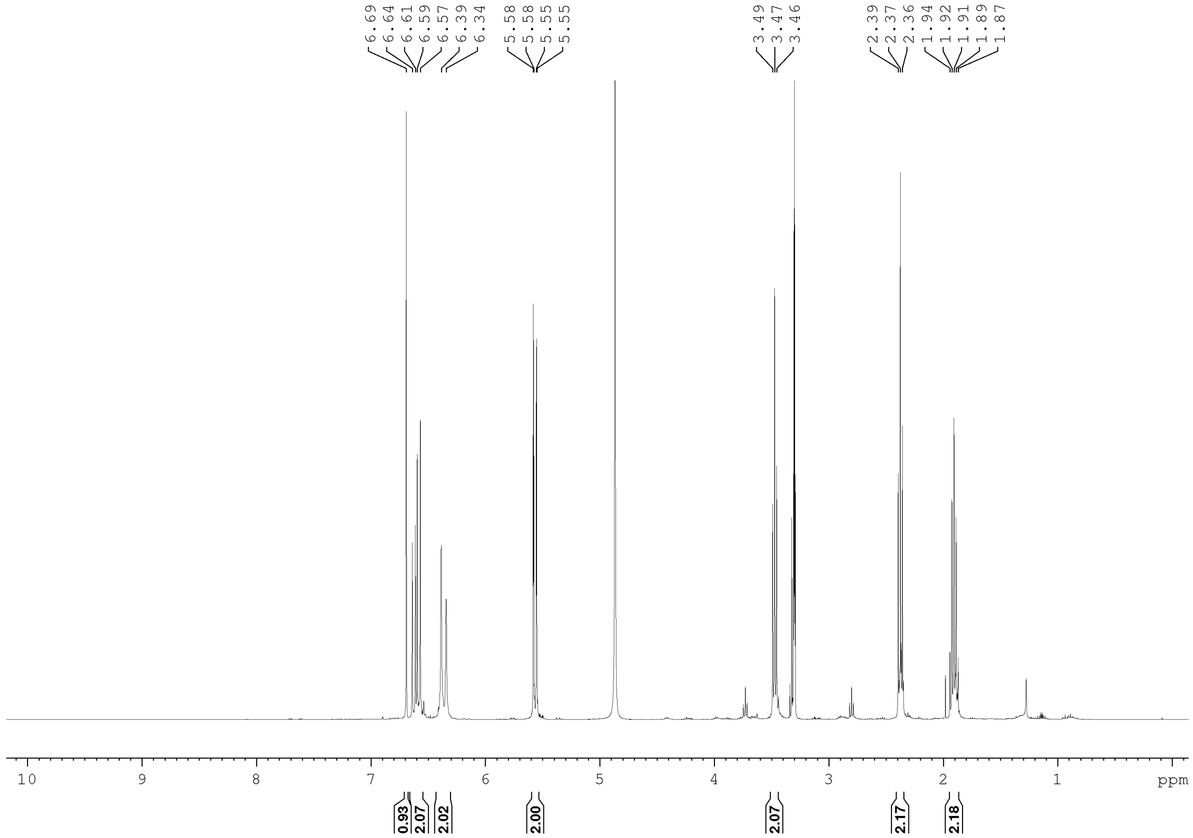

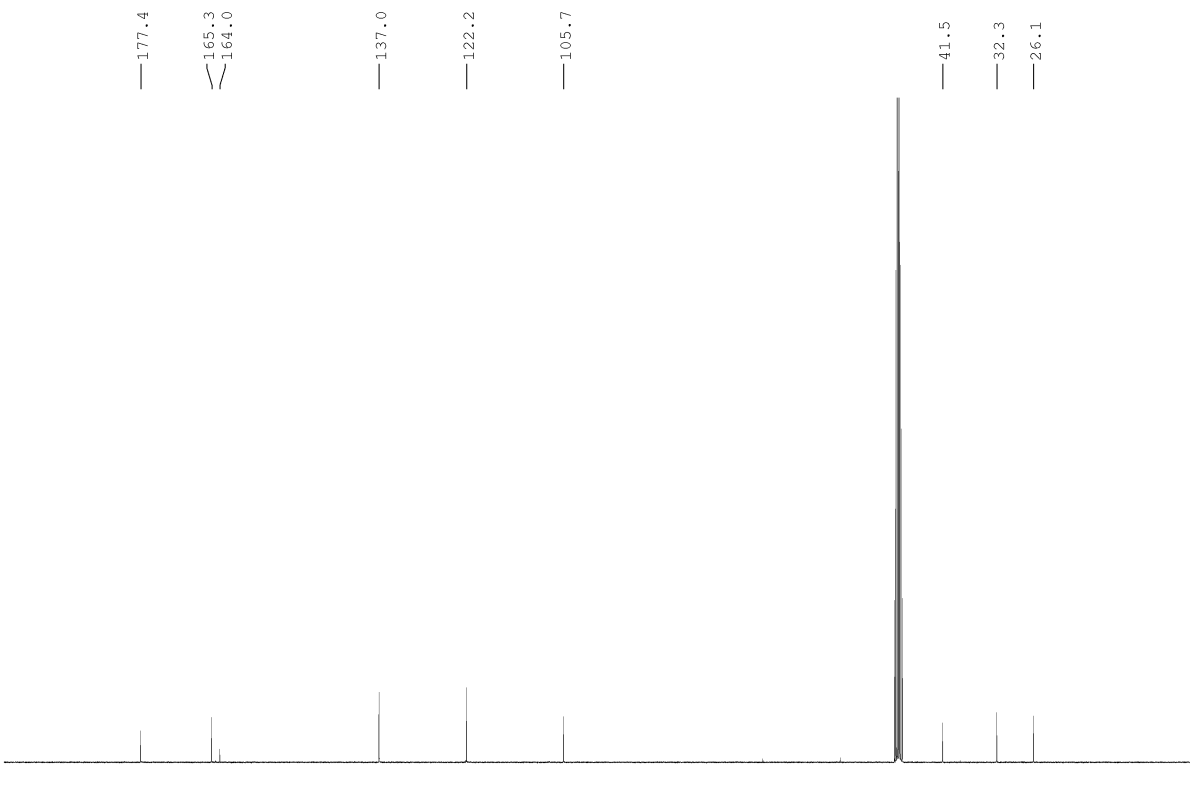

_N_-(2-(2-(2-(2-azidoethoxy)ethoxy)ethoxy)ethyl)-4-((4,6-divinylpyrimidin-2-yl)amino)butanamide (S16)

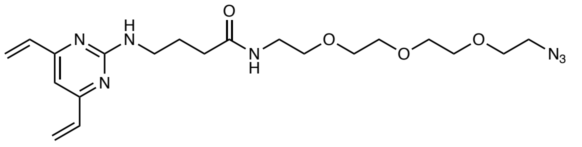

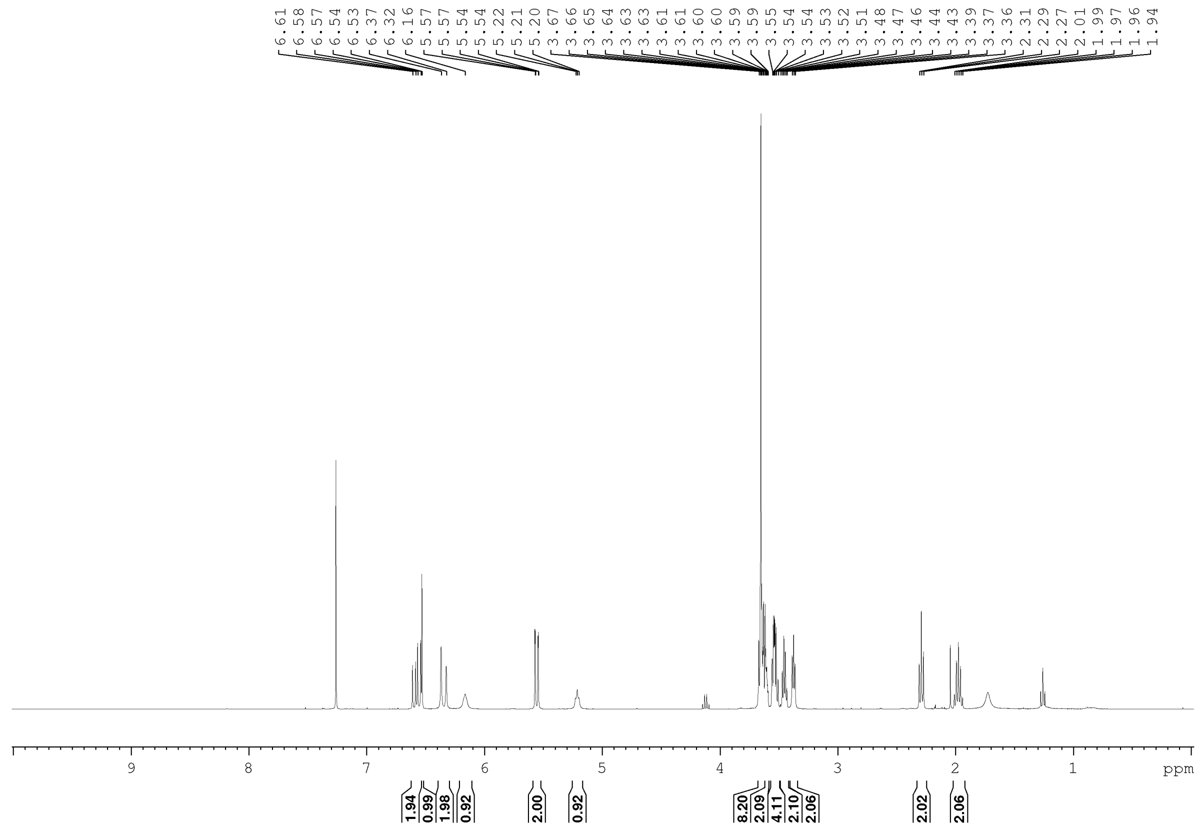

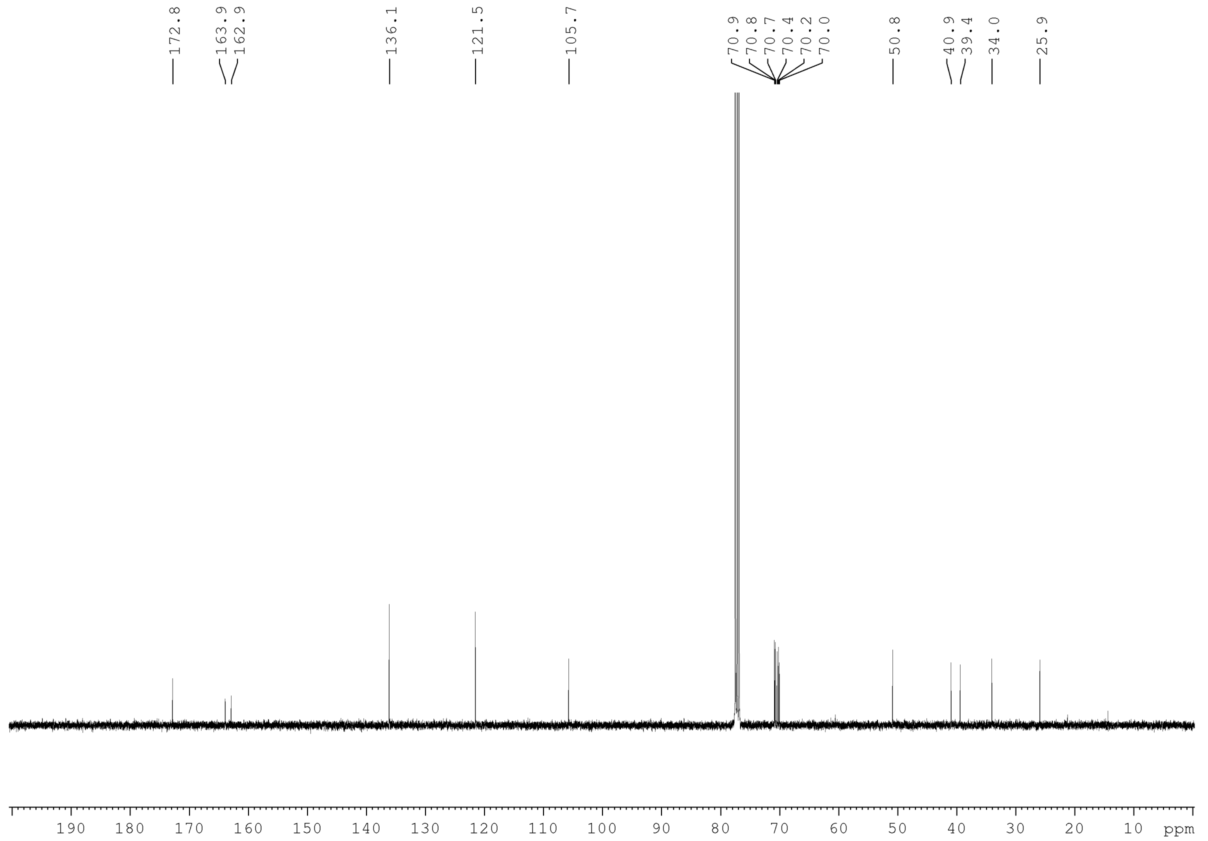

Alloc-Val-Ala-PABC-AMC (S17)

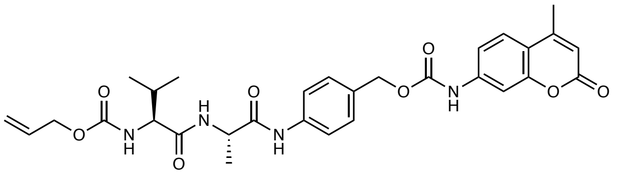

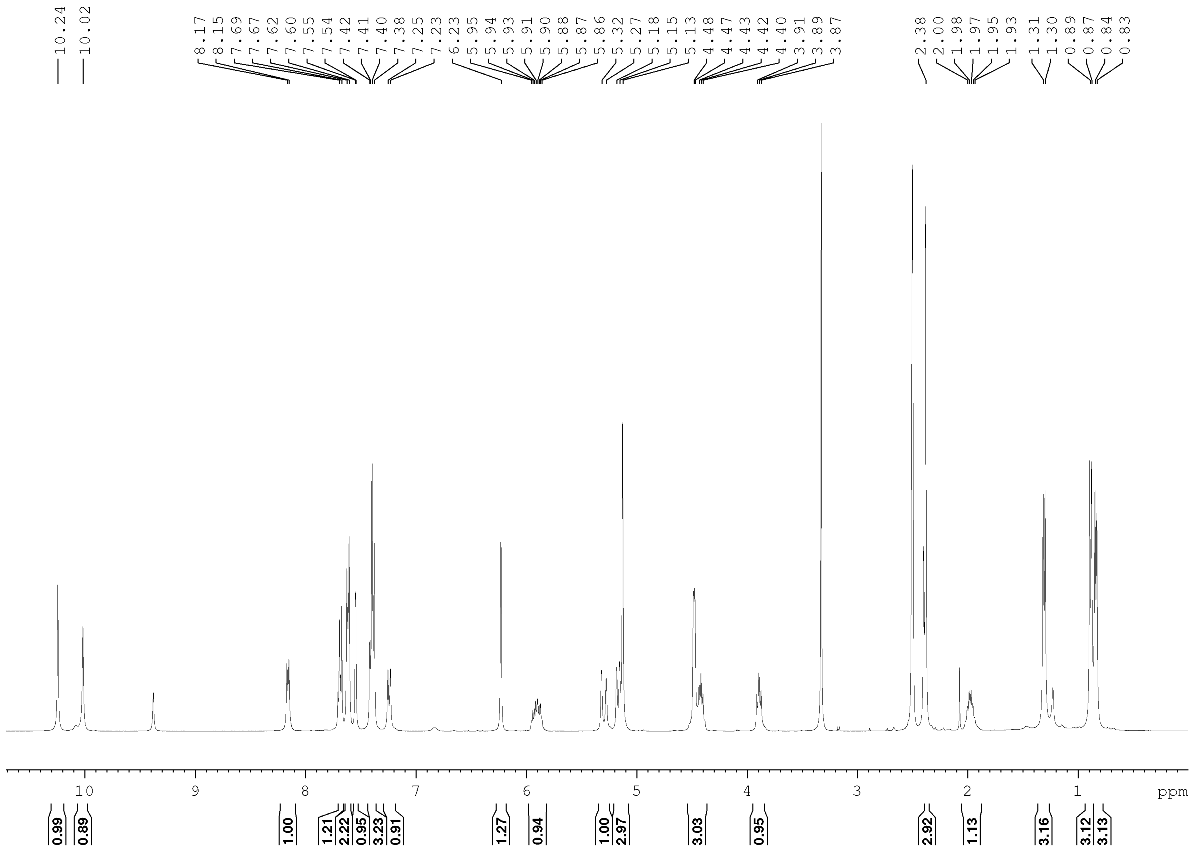

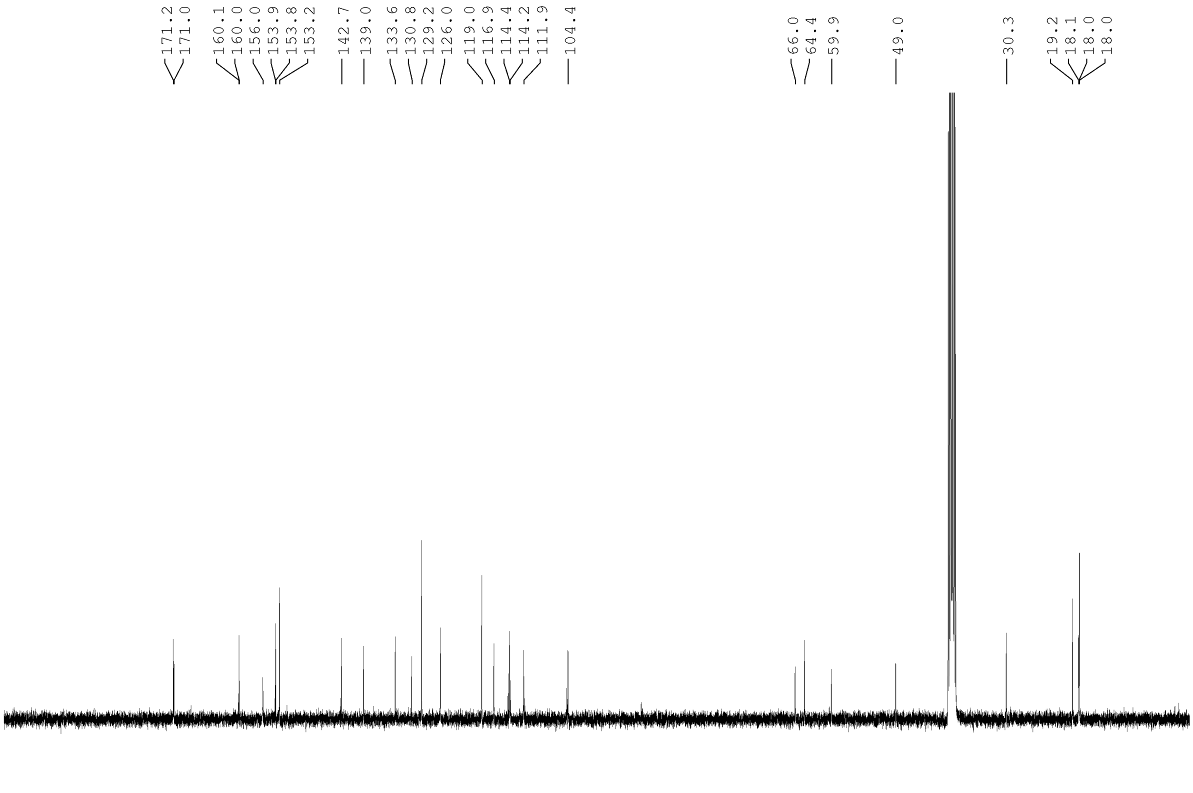

H_2N-Val-Ala-PABC-AMC (S18)

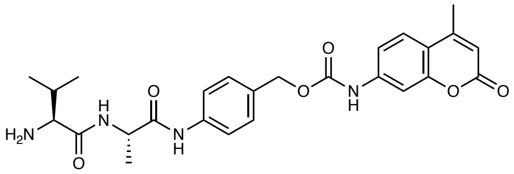

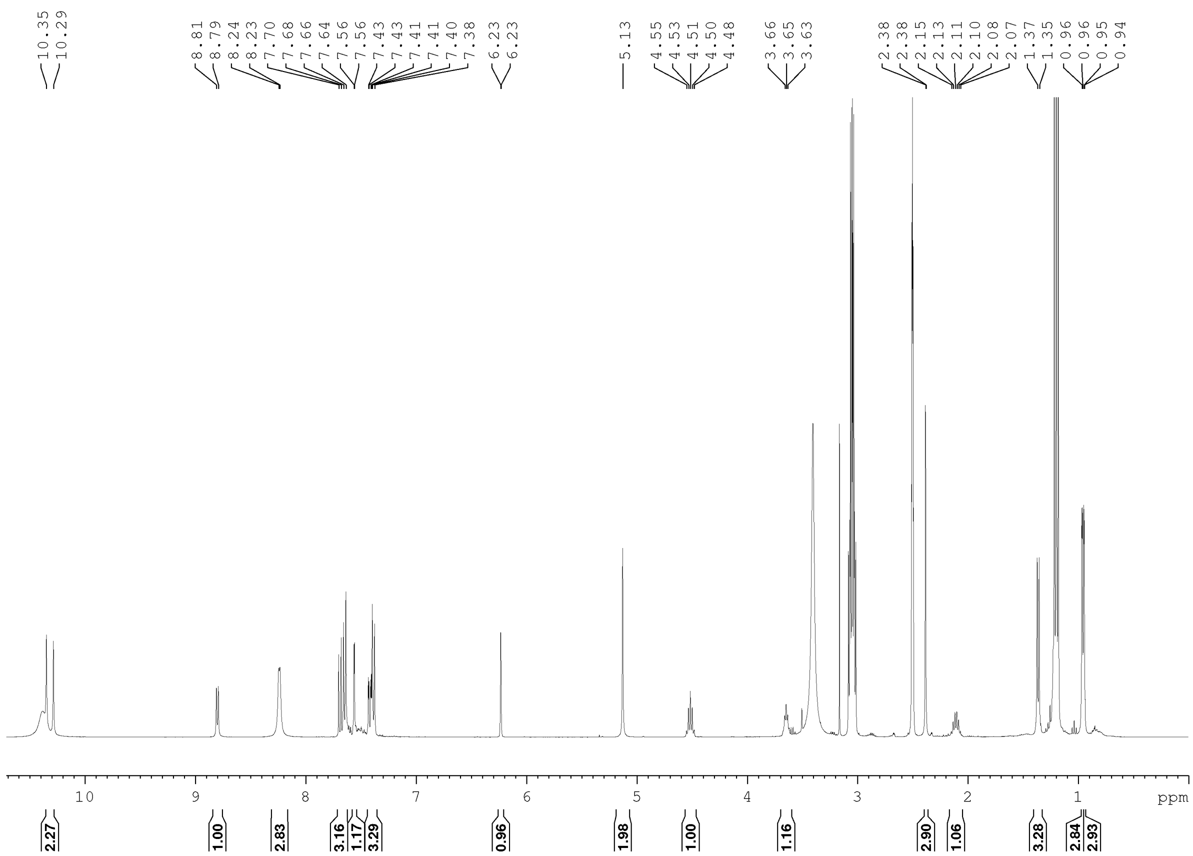

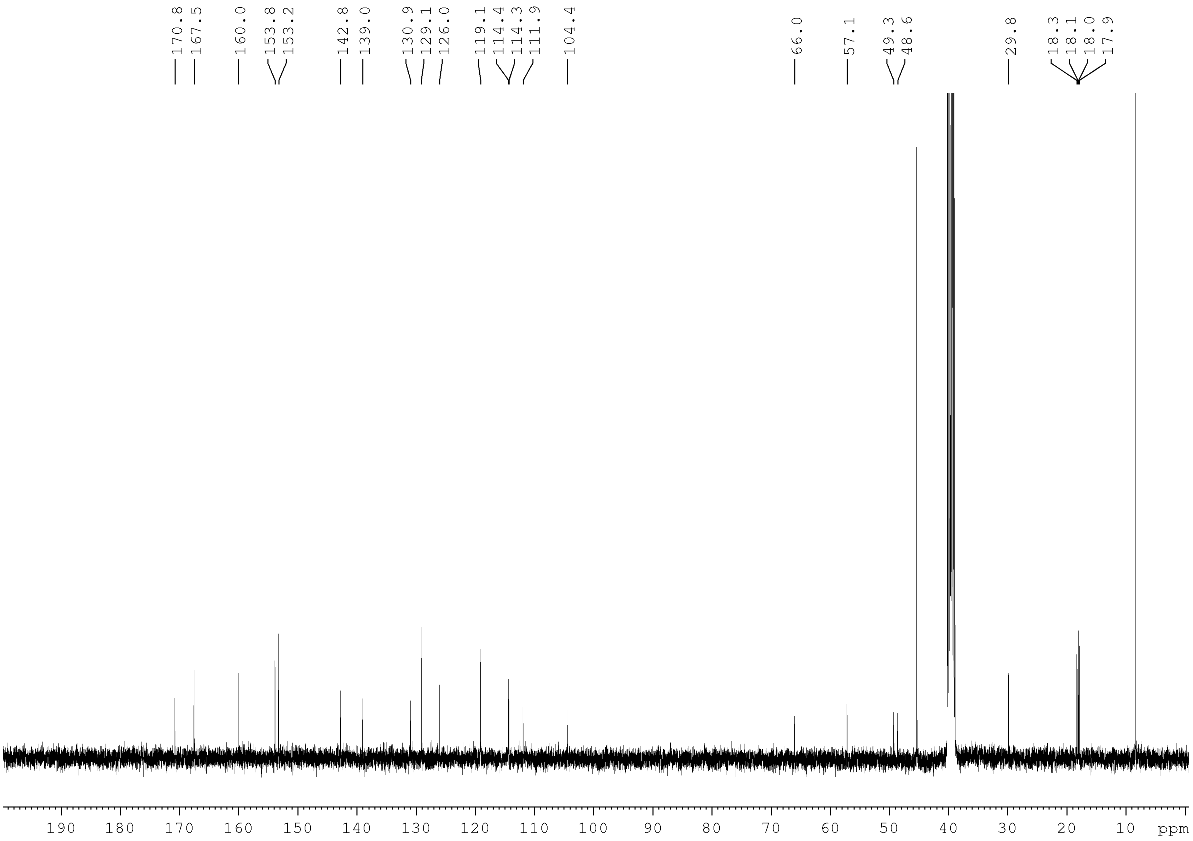

4-((*S*)-2-((*S*)-2-(2-methoxyacetamido)-3-methylbutanamido)propanamido)benzyl (4-methyl-2-oxo-2*H*-chromen-7-yl)carbamate (13)

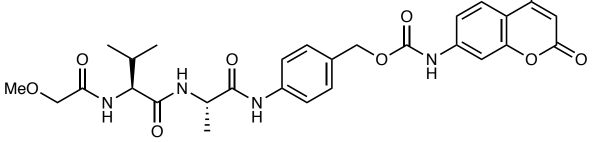

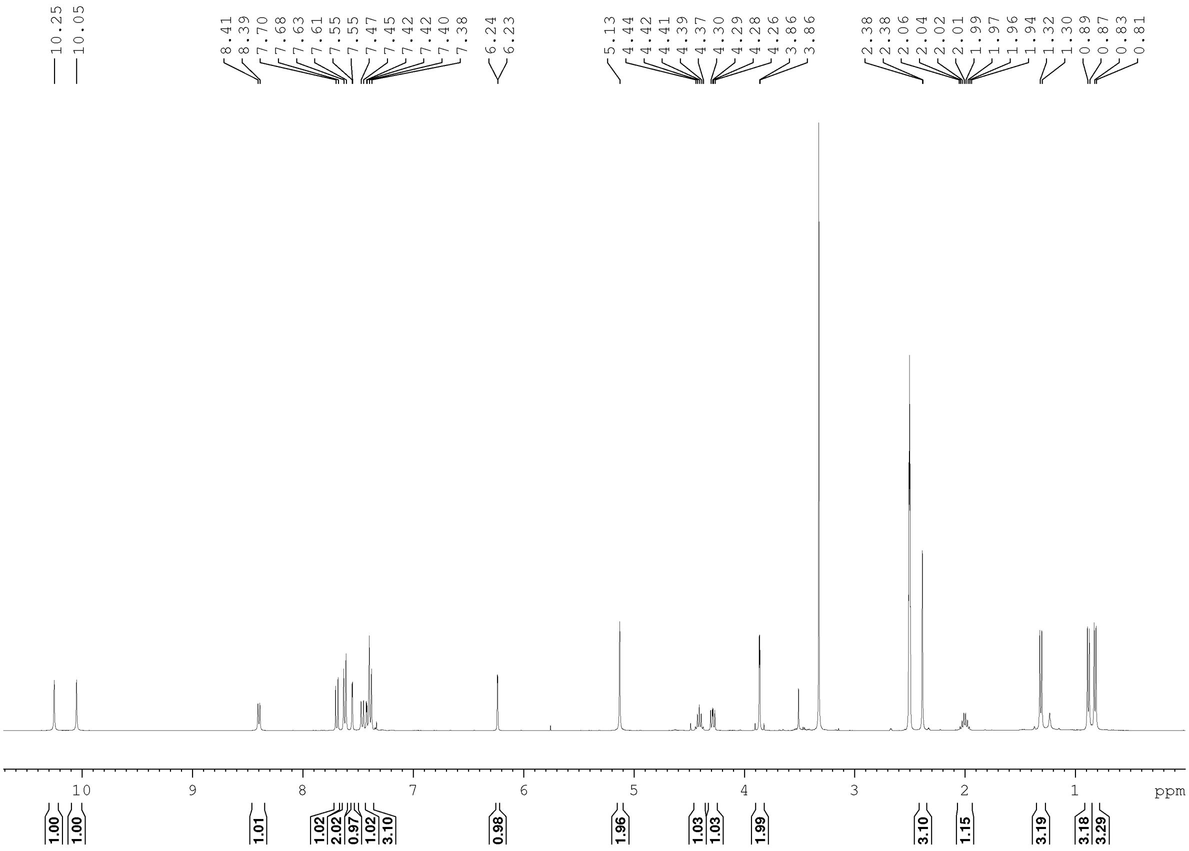

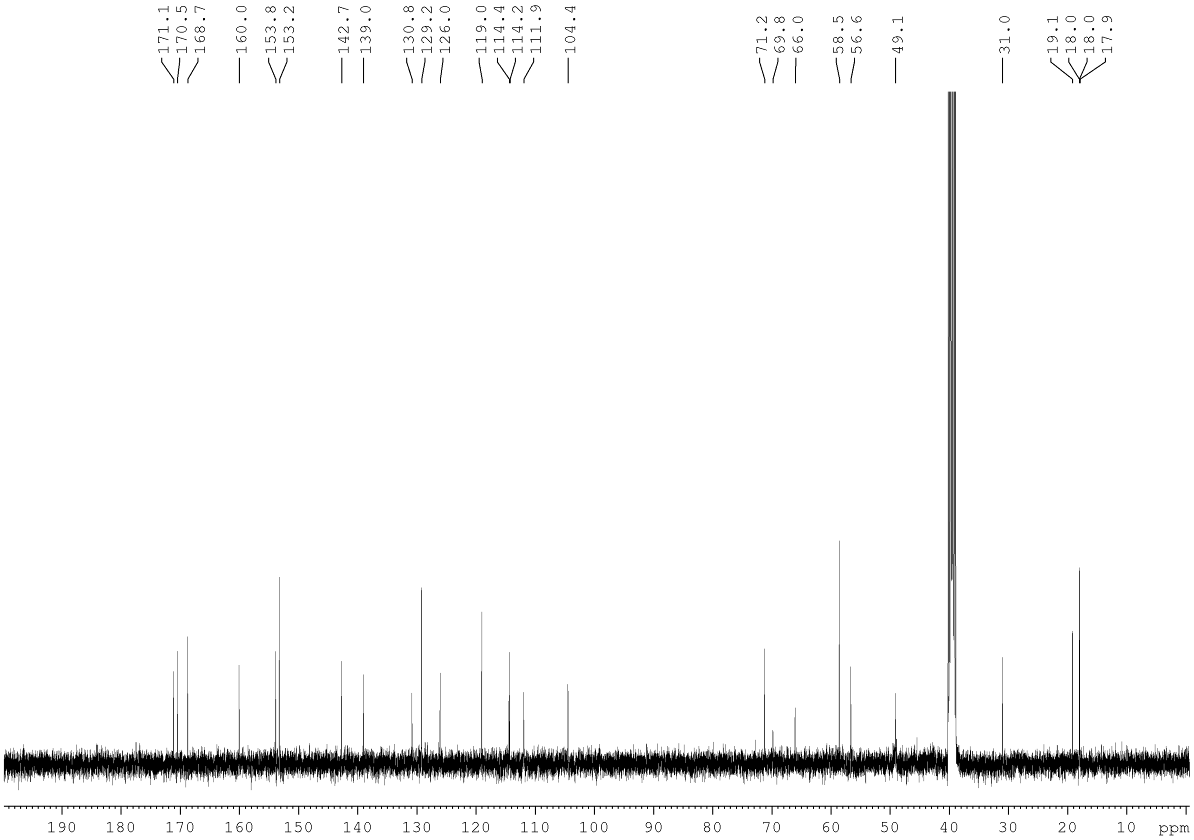

Fmoc-Val-Cit-OH (S19)

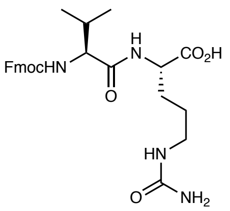

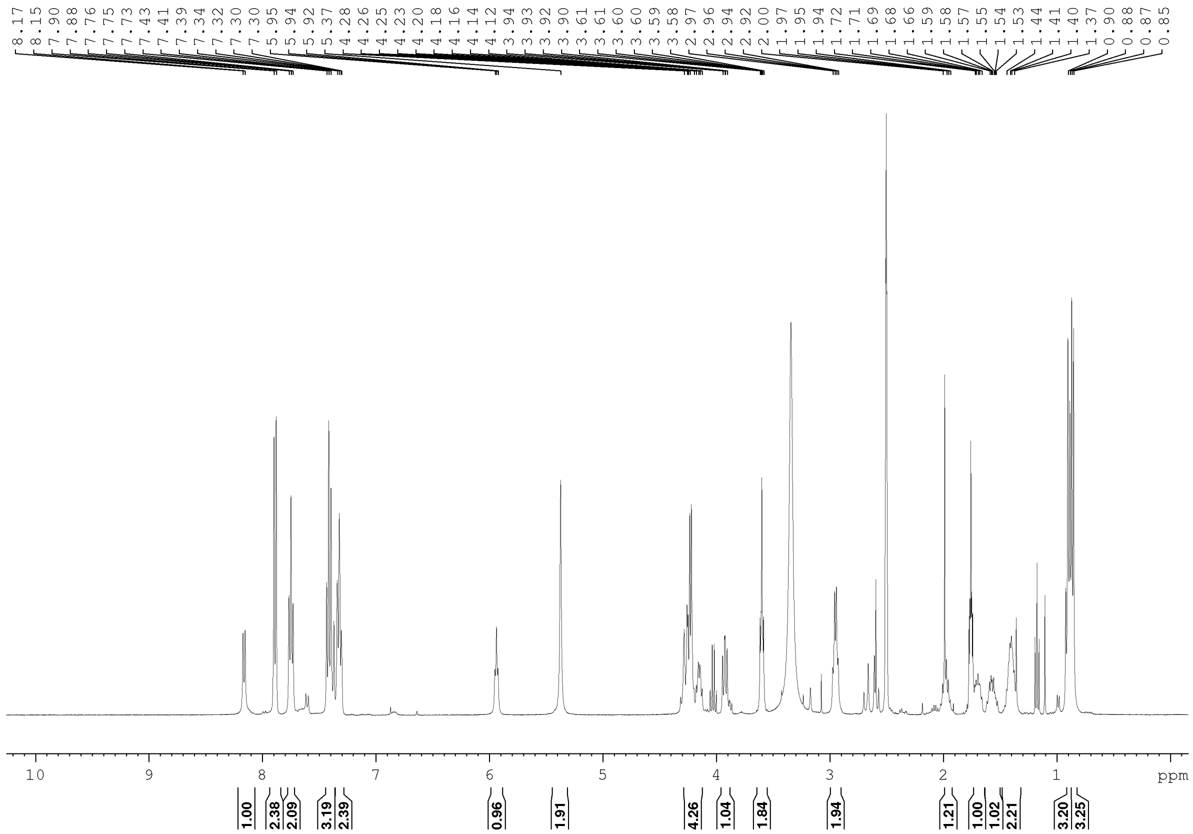

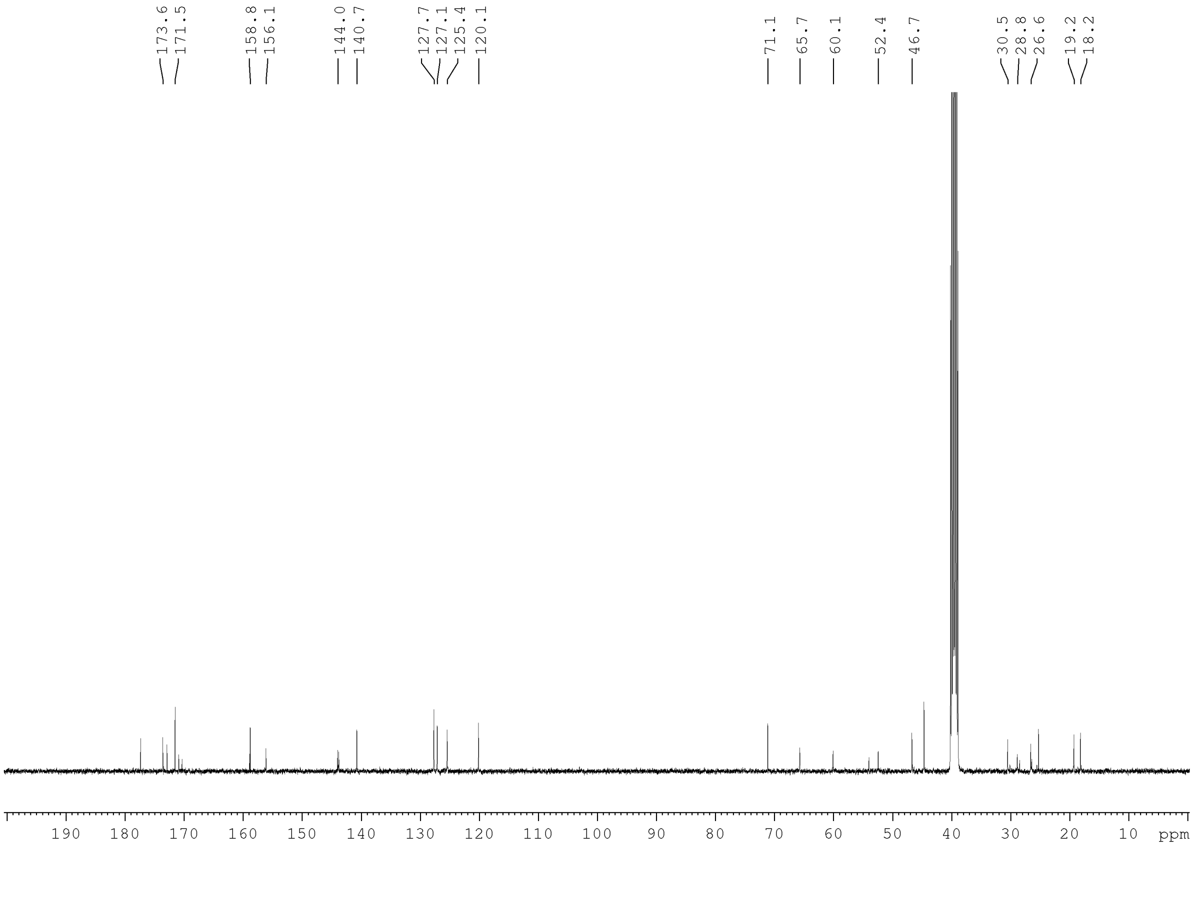

Fmoc-Val-Cit-PABOH (S20)

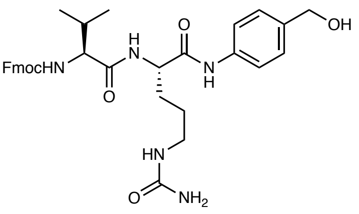

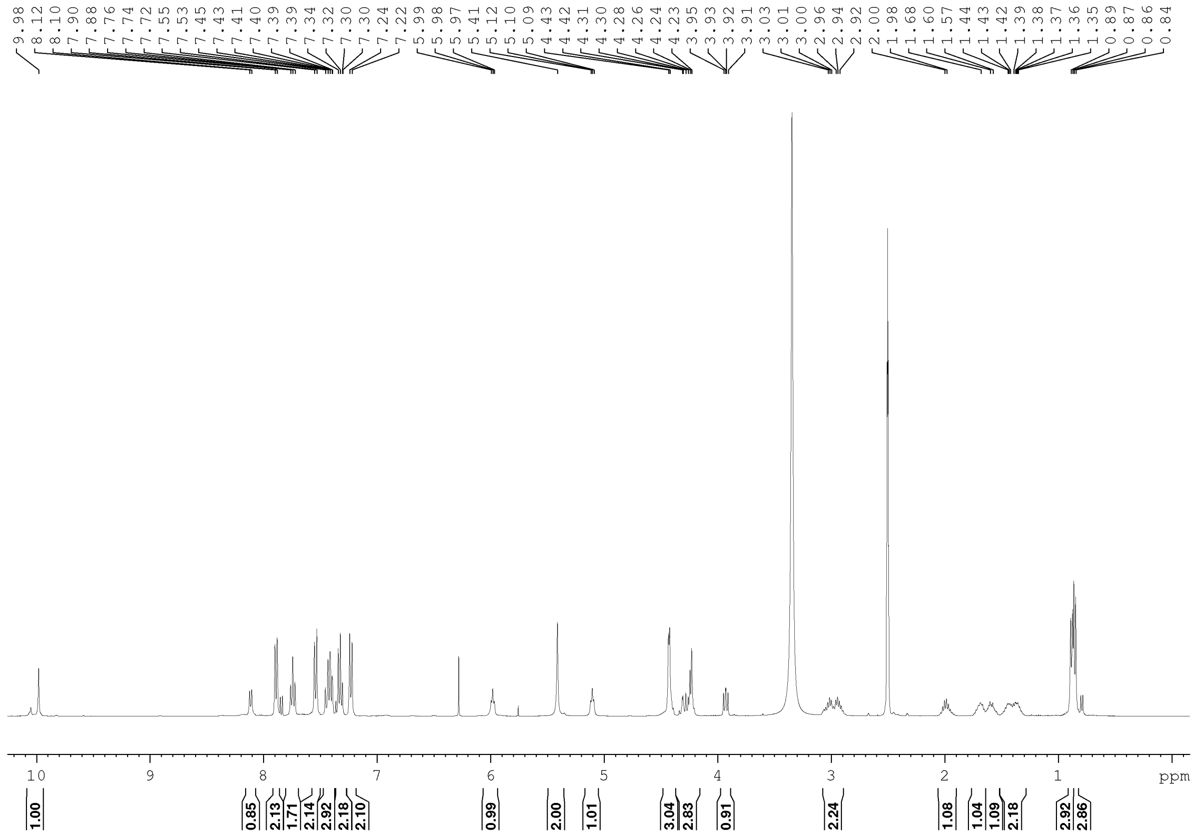

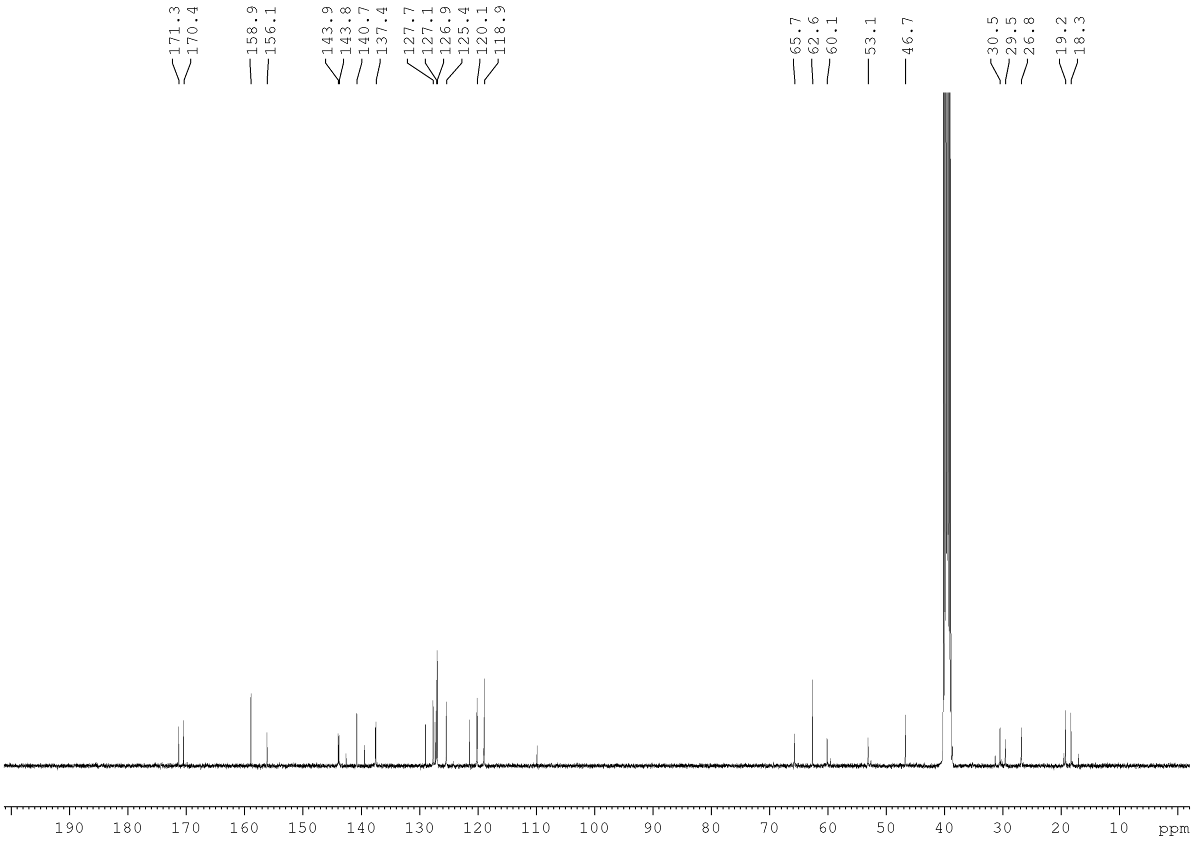

(*S*)-*N*-(4-(hydroxymethyl)phenyl)-2-((*S*)-2-(2-methoxyacetamido)-3-methylbutanamido)-5-ureidopentanamide (S21)

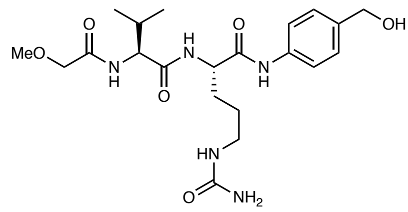

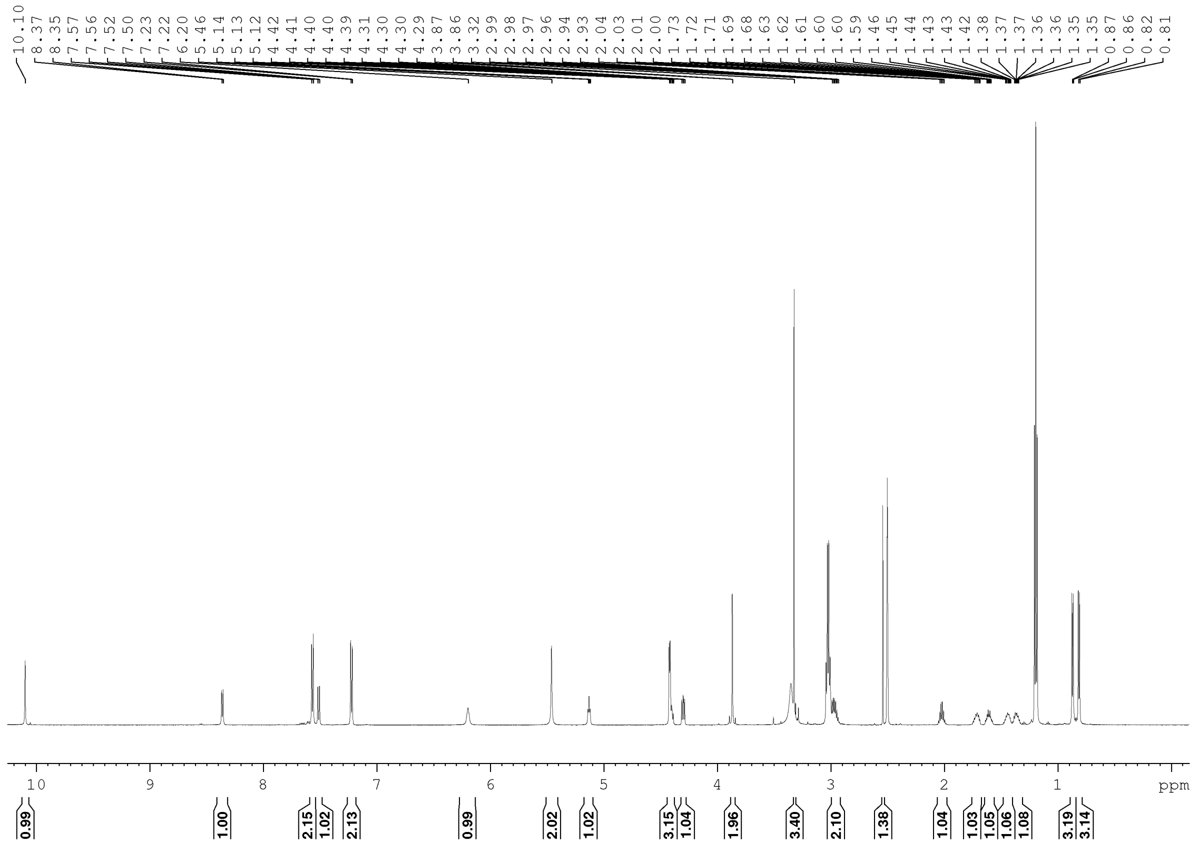

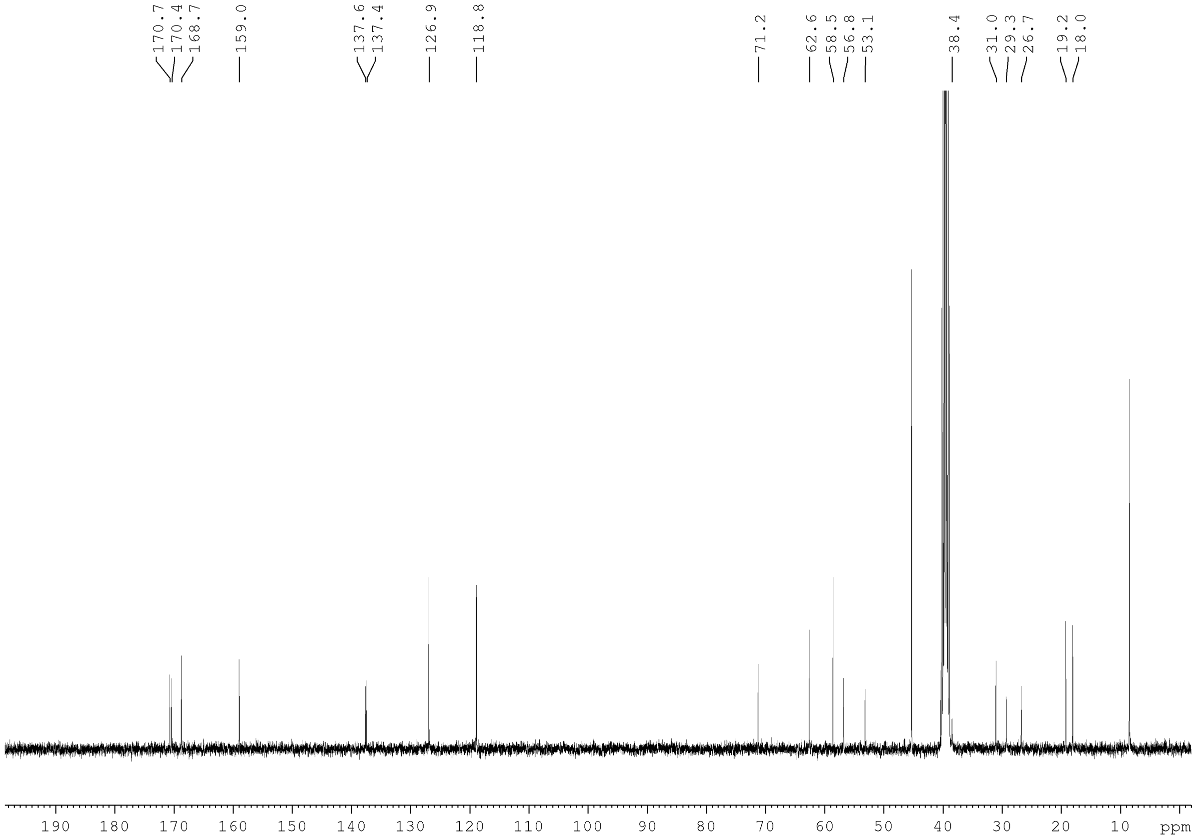

4-((*S*)-2-((*S*)-2-(2-methoxyacetamido)-3-methylbutanamido)-5-ureidopentanamido)benzyl (4-methyl-2-oxo-2*H*-chromen-7-yl)carbamate (14)

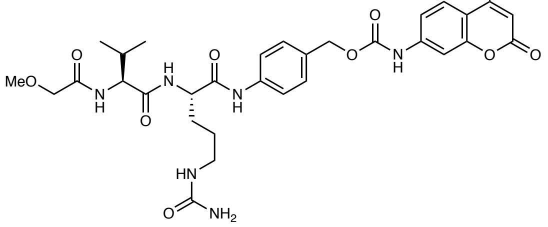

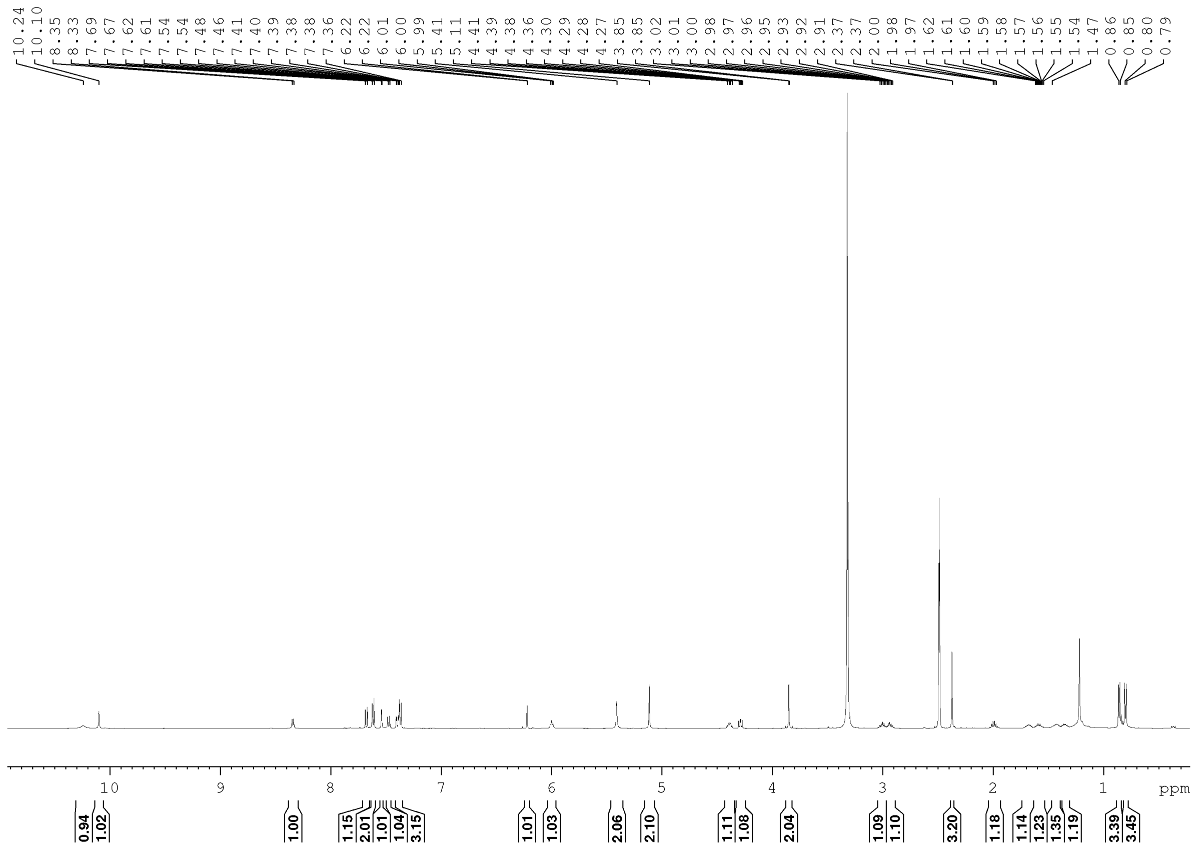

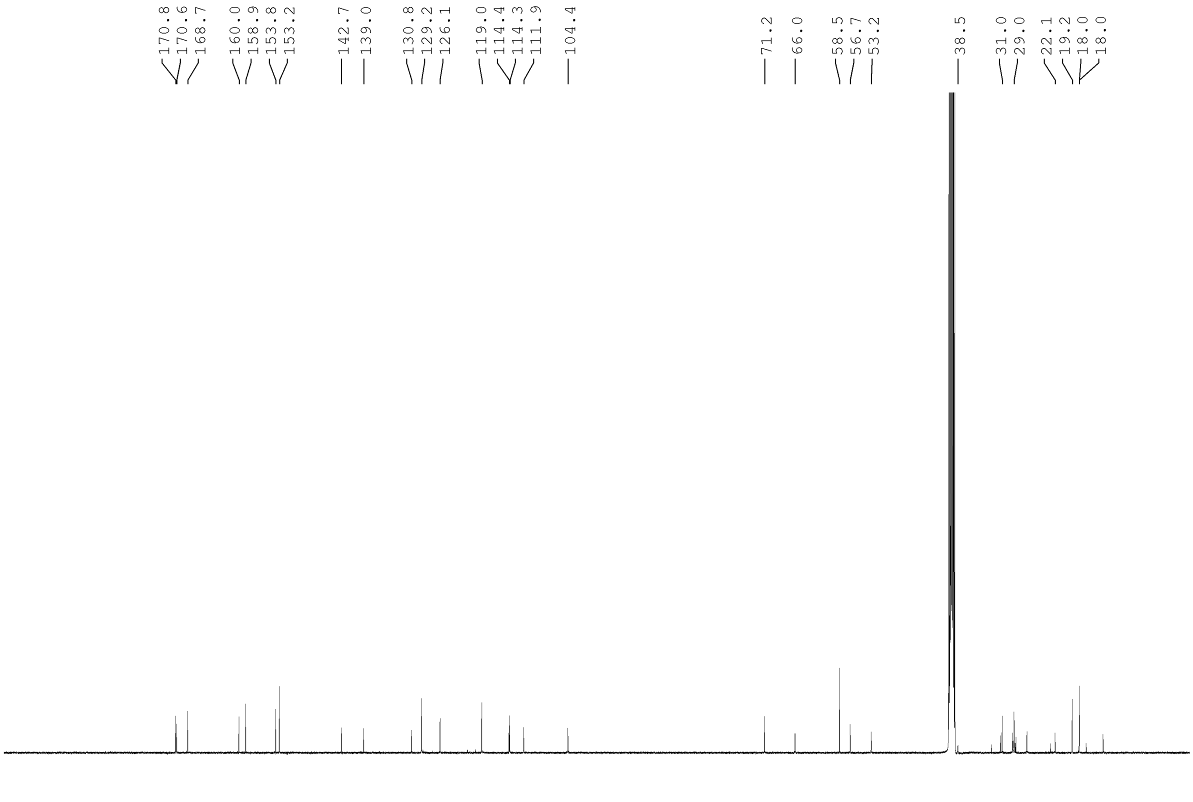

N_3-PEG$_4$-Val-Ala-PABC-MMAE (S22)

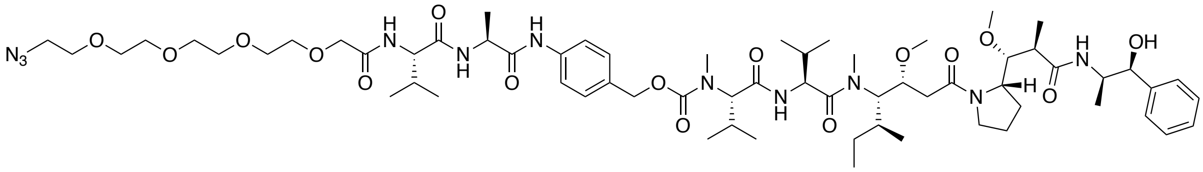

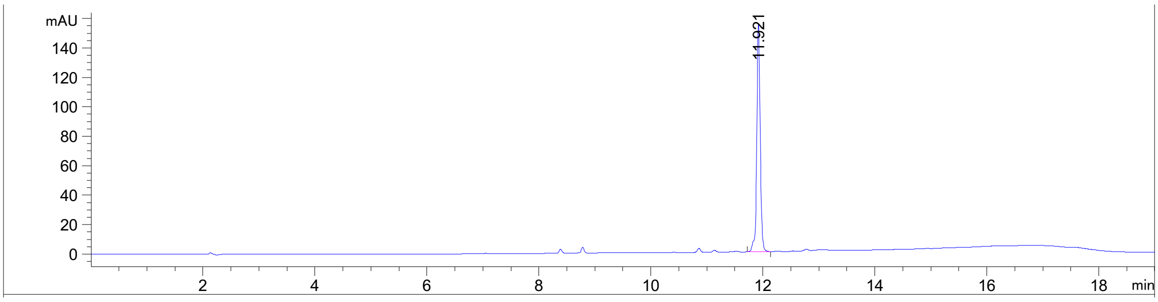

DVP-PEG$_4$-Val-Ala-PABC-MMAE (18)

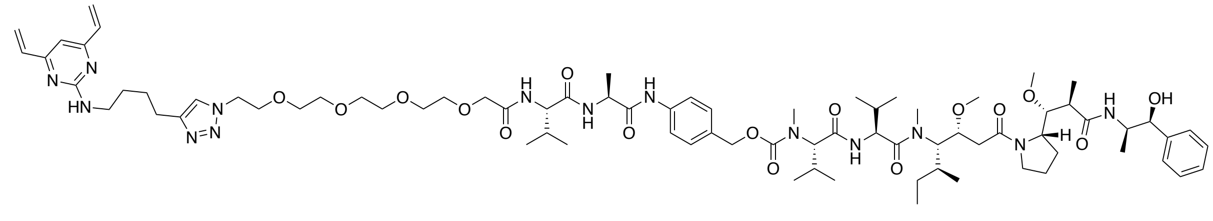

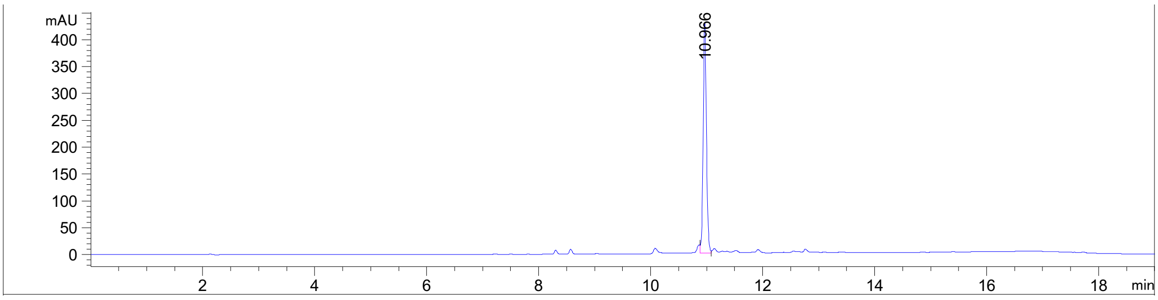

Phenyl sulfamate (S24)

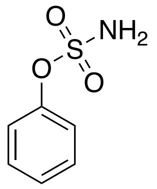

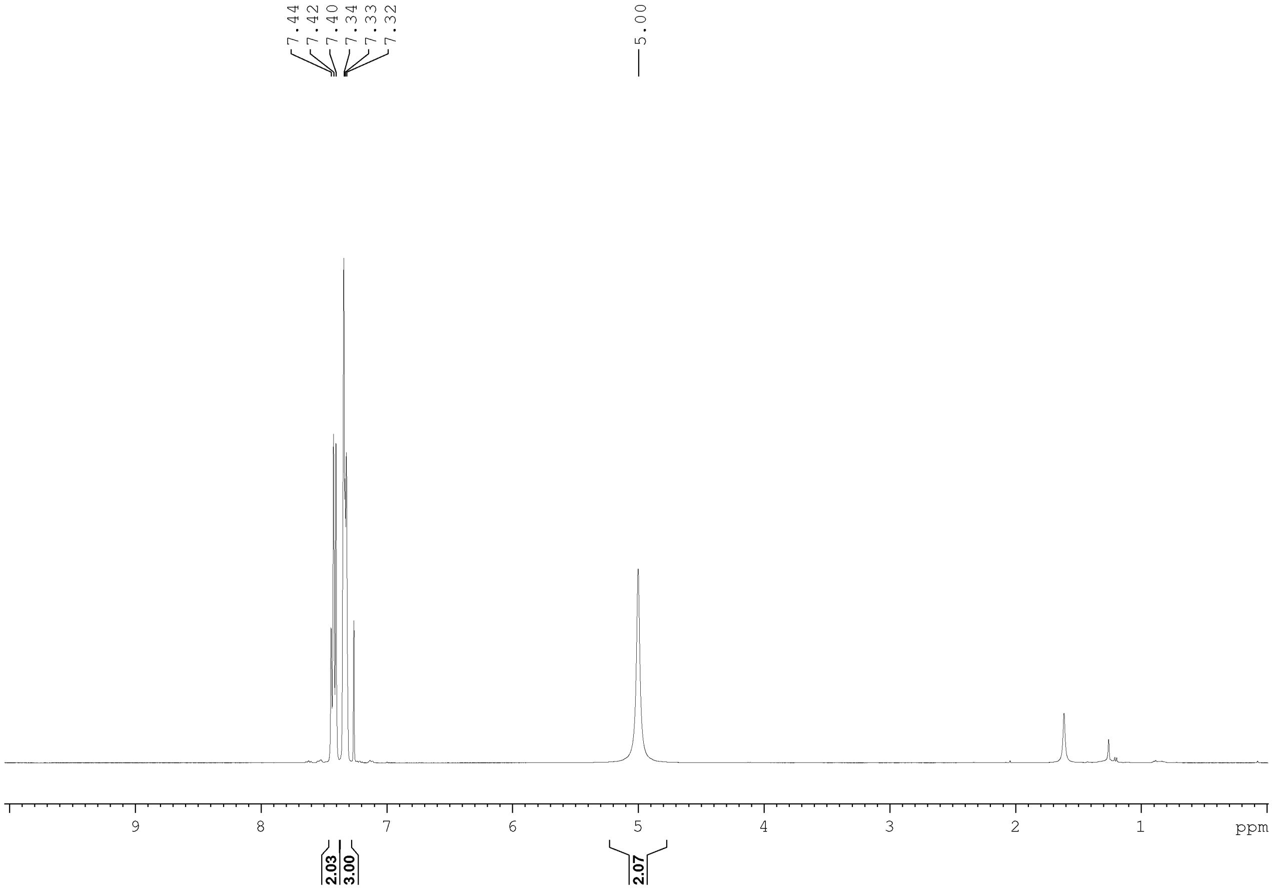

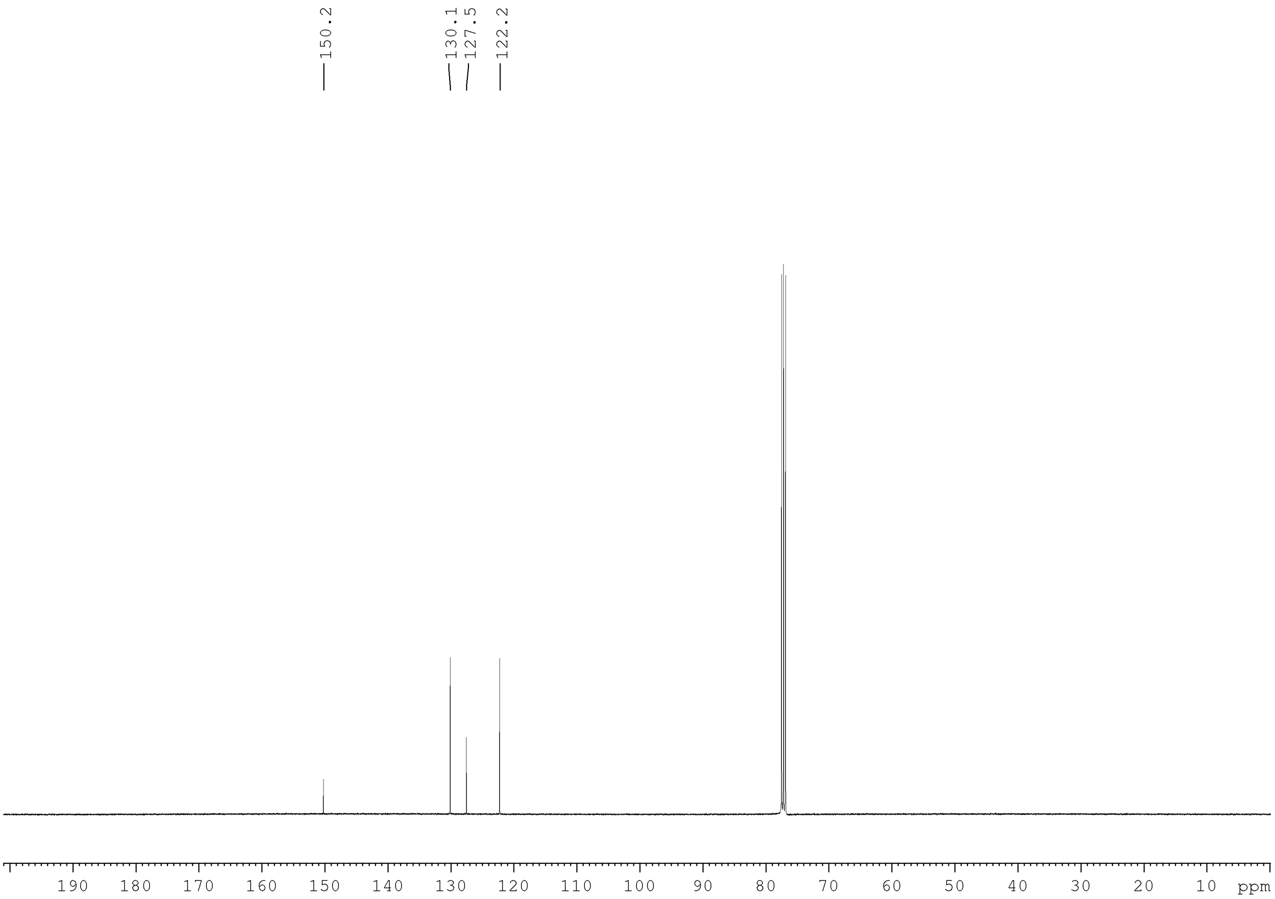

Supplement: SC-011-C9SC06410A-s001 [file SC-011-C9SC06410A-s001.pdf]
